# Design, synthesis, and antiproliferative activity of new 5-ethylsulfonyl-indazole-3-carbohydrazides as dual EGFR/VEGFR-2 kinases inhibitors

**DOI:** 10.1080/14756366.2025.2516075

**Published:** 2025-06-24

**Authors:** Lamya H. Al-Wahaibi, Hesham A. Abou-Zied, Mohamed A. Mahmoud, Bahaa G. M. Youssif, Stefan Bräse, Safwat M. Rabea

**Affiliations:** a Department of Chemistry, College of Sciences, Princess Nourah Bint Abdulrahman University, Riyadh, Saudi Arabia; b Medicinal Chemistry Department, Faculty of Pharmacy, Deraya University, Minia, Egypt; c Pharmaceutical Organic Chemistry Department, Faculty of Pharmacy, Assiut University, Assiut, Egypt; d Institute of Biological and Chemical Systems, IBCS-FMS, Karlsruhe Institute of Technology, Karlsruhe, Germany; e Medicinal Chemistry Department, Faculty of Pharmacy, Minia University, Minia, Egypt; f Apogee Pharmaceuticals, Burnaby, BC, Canada

**Keywords:** Apoptosis, DFT, cancer, EGFR, VEGFR-2

## Abstract

A novel series of 5-ethylsulfonyl-indazole-3-carbohydrazides **7a–o**, serving as dual inhibitors of EGFR and VEGFR-2 was developed. The antiproliferative effects of compounds **7a–o** were assessed against four cancer cell lines via the MTT assay. Compounds **7g**, **7i–7l**, and **7o** emerged as the most efficient six derivatives, with GI_50_ values ranging from 25 nM to 42 nM. Compounds **7j**, **7k**, and **7o** (GI_50_ values of 27, 25, and 30, respectively) demonstrated greater potency than erlotinib (GI_50_ value of 33 nM), particularly against breast (MCF-7) cancer cell lines, and were identified as the most potent dual EGFR/VEGFR-2 inhibitors. Apoptotic markers assay results showed that increased levels of p53 and Bax proteins, along with lower levels of antiapoptotic Bcl-2, govern the apoptosis process in these new compounds. Computational analyses, encompassing molecular docking, molecular dynamics (MD) simulations, and density functional theory (DFT) computations, elucidated the binding interactions of these drugs with EGFR and VEGFR-2.

## 1. Introduction

Cancer ranks among the foremost causes of global human death; thus, there has been significant focus on its treatment worldwide^1^. In comparison to radiotherapy and biological therapy, chemotherapy remains as the cornerstone of contemporary treatment. Nonetheless, a wide range of these medications is constrained by a limited therapeutic index and often encounters the development of resistance^2^^,^^3^. Therefore, the urgent necessity for the discovery of innovative anticancer agents characterised by high efficacy and low toxicity persists.

Nitrogen-containing heterocycles are pharmacologically significant frameworks and are prevalent in many commercially available pharmaceuticals^4^^,^^5^. Indazole analogs, a significant family of nitrogen-containing heterocycles, have garnered substantial attention historically and in recent years due to their diverse biological properties, including anticancer^6^^,^^7^, anti-inflammatory^8^, antimicrobial^9^, and antihypertensive^10^ activities. Some indazole-based therapeutic drugs, including pazopanib, axitinib, and entrectinib, which are receptor tyrosine kinase inhibitors, are approved for treatment of cancer^11–13^.

**Figure 1.**
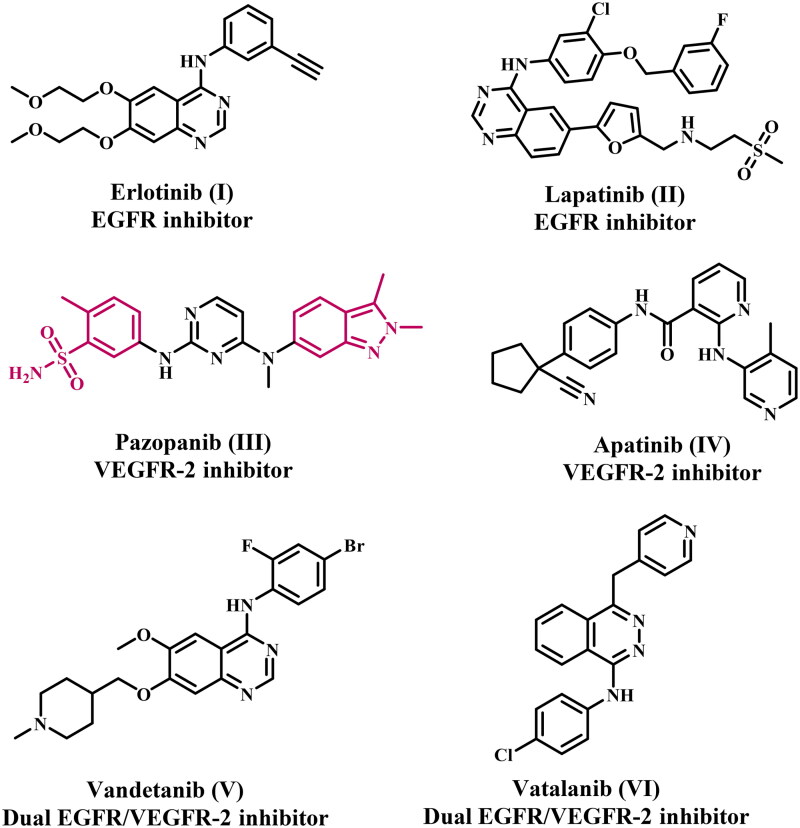
Representative examples of FDA-approved EGFR and/or VEGFR-2 inhibitors.

The human receptor tyrosine kinase (RTK) family comprises 58 proteins categorised into 20 subfamilies^14^. These RTKs play a crucial role in regulating cell proliferation, differentiation, apoptosis, adhesion, and migration. Nevertheless, hyperactivation of RTKs may lead to the onset of various cancers^15–17^. As a result, the suppression of RTK activity is increasingly recognised as a prevalent approach in cancer treatment. The epidermal growth factor receptor (EGFR) and vascular endothelial growth factor receptor (VEGFR-2) are prevalent RTKs^18^^,^^19^. EGFR plays a crucial role in regulating various biological activities, including cell survival, proliferation, and migration^20^. Conversely, VEGFs are recognised as one of the most specific and essential pro-angiogenic signalling factors implicated in angiogenesis across diverse human malignancies^21^. Additionally, a wide range of human malignancies have been found to overexpress these two kinases^22^. A multitude of clinically approved anticancer drugs, such as Erlotinib, Lapatinib, Pazopanib, Apatinib, Vandetanib, and Vatalanib (Figure 1), exhibit potent inhibitory effects on EGFR and/or VEGFR-2^23^^,^^24^. Consequently, inhibiting the EGFR and VEGFR-2 signalling pathways is now recognised as a promising strategy for developing new antiproliferative drugs.

This interrelationship highlights the value of designing dual-target inhibitors to overcome such resistance mechanisms. Krisyanti Budipramana et al. reviewed the crosstalk between EGFR and VEGFR-2 pathways and the structural considerations essential for designing selective dual inhibitors, emphasising the importance of molecular docking in understanding inhibitor selectivity^25^. Additionally, authors underscored the utility of integrated CADD approaches such as pharmacophore modelling, virtual screening, docking, MD simulations, and MM/PB(GB)SA energy calculations in identifying VEGFR-2 inhibitors with enhanced pharmacokinetic and toxicity profiles^26^. These studies strongly support the application of an integrated *in silico* and experimental framework in the discovery of multi-target kinase inhibitors.

**Figure 2.**
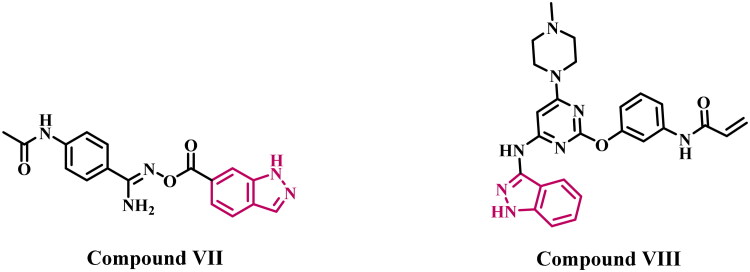
Structure of some indazole-based derivatives (**VII** and **VIII**) as EGFR inhibitors.

Recent reports have extensively documented the development of indazole derivatives that target the EGFR and VEGFRs. In 2022^27^, we present the design, synthesis, and antiproliferative efficacy of new indazole-based derivatives. The newly synthesised compounds were assessed for their antitumor efficacy against a panel of four different cancer cell lines. The most effective compounds were further examined as EGFR inhibitors. Compound **VII** (Figure 2) exhibited the highest potency as an EGFR inhibitor, with an IC_50_ value of 85 ± 5 nM, comparable to the reference erlotinib, which has an IC_50_ value of 80 ± 5 nM. Furthermore, compound **I** triggered apoptosis through the overexpression of cytochrome *c*, activation of caspases 3, 8, and 9, as well as the activation of Bax and the downregulation of the antiapoptotic protein Bcl-2.

Engel et al.^28^ introduced an indazole-based analog **VIII** (Figure 2), which was found to be the most effective EGFR inhibitor among a group of indazole derivatives. Compound **VIII** had IC_50_ values of 0.07, 0.50, and 1.70 µM for EGFR^T790M^, EGFR^L858R^, and wild-type EGFR, respectively. Compound **II** had a substantially stronger inhibitory effect on the drug-resistant EGFR mutation. Docking research demonstrated that the indazole scaffold fills the gap between the hinge region and the gatekeeper residues within the EGFR binding site without encountering steric issues with the methionine side chain. The indazole moiety made a significant contribution to the formation of hydrogen bonds with the residues Glu339 and Met341. The phenyl ring of the indazole facilitated favourable hydrophobic interactions, leading to enhanced protein–ligand interactions.

**Figure 3.**
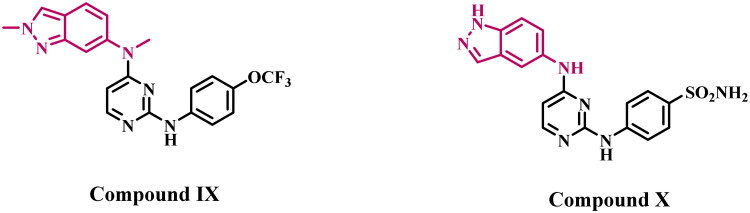
Structure of some indazole-based derivatives (**IX** and **X**) as VEGFR-2 inhibitors.

Qi et al.^29^ reported a study in which a series of pazopanib-based derivatives had been substituted with an indazole ring to examine the electronic and steric effects of substituents. Compound **IX** (Figure 3) exhibited the highest activity among the synthesised compounds, demonstrating enhanced efficacy against VEGFR-2 kinase with an IC_50_ value of 12 nM, compared to pazopanib, which has an IC_50_ value of 30 nM. In another study^30^, the authors identified compound **X** (Figure 3), which features a sulphonamide on the aniline ring at the C4 position, as the most effective derivative within a novel series of indazole-based VEGFR-2 inhibitors. Compound **X** exhibited an IC_50_ value of 24.5 nM, comparable to that of the reference pazopanib (IC_50_ = 25 nM). Molecular modelling studies have demonstrated that the 2-aminopyrimidine moiety forms two hydrogen bonds with Cys919 in the hinge region. In contrast, the indazole ring engages in hydrophobic interactions with residues Leu840, Val848, Ala866, and Leu1035, as well as hydrogen-bonding interactions with Gly843.

**Figure 4.**
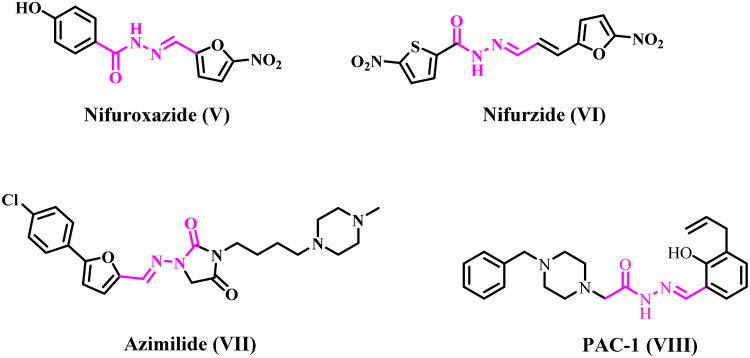
Structures of some NAH-based clinically approved drugs **V–VII** and **PAC-1 (VIII)**.

The bioactive N-acylhydrazone (NAH) core has emerged as one of the most prevalent functional groups in medicinal chemistry, discovered in numerous hit and lead compounds that interact with diverse molecular targets^31–33^. Medicinal chemists have undertaken extensive endeavours to develop novel favoured small-molecule scaffolds through the development of innovative synthetic transformations utilising the potent NAH core. The usefulness of N-acylhydrazones in medicinal chemistry stems from their facile synthesis, typically achieved through a condensation reaction involving aldehydes or ketones and hydrazides^34^. Several research have been published about the chemistry and bioactive lead scaffolds of N-acylhydrazones, and throughout time, this topic has gained in importance for the development of new, therapeutically relevant bioactive NAH candidates. Several derivatives featuring the acylhydrazone moiety are utilised therapeutically, including nitrofurazone (antimicrobial), nifuroxazide (intestinal antibacterial), nitrofurantoin (antibacterial), nifurzide (intestinal anti-infective), azimilide (anti-arrhythmic), and zorubicin (cytotoxic antibiotic)^35^. Figure 4 illustrates the structures of some representative pharmacologically active drugs featuring the acylhydrazone scaffold. In addition, a NAH small-molecule pro-caspase activator, **PAC-1** (**VIII**) (Figure 4), is entering phase 1 clinical trials (NCT02355535) in 2015^36^. **PAC-1**’s development began with the discovery of the NAH small scaffold procaspase activator, which showed promise anticancer activity in both *in vitro* and *in vivo* models^37^. Botham and co-workers found that **PAC-1** promotes apoptosis when combined with 15 different FDA-approved chemotherapeutics across a wide range of cancer types and targets^38^.

**Figure 5.**
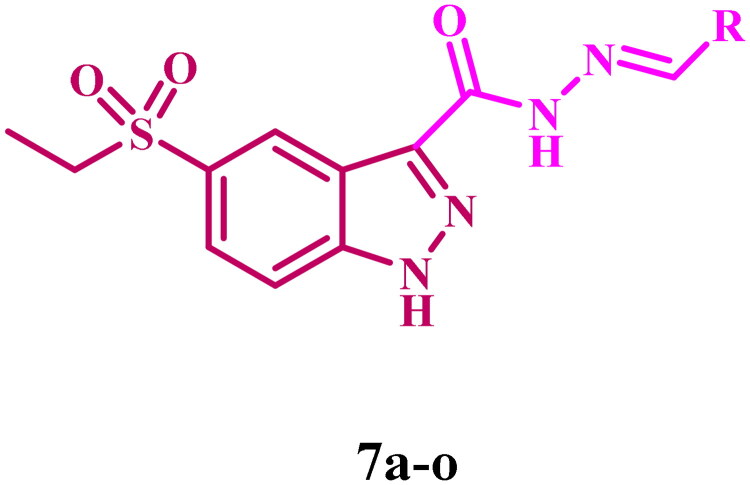
Structures of 5-(ethylsulfonyl)-1*H*-indazole-3-carbohydrazides **(7a–o)**.

In the quest for improved anticancer agents and as part of our ongoing efforts to discover dual- or multi-targeted antiproliferative agents^39–44^, we introduce the design, synthesis, and antiproliferative activity of a novel series of 5-ethylsulfonyl-indazole-3-carbohydrazide (**7a–o**, Figure 5) as dual inhibitors of EGFR and VEGFR-2. We considered the biological significance of indazole-based derivatives as inhibitors of EGFR and VEGFR-2, which are known for their anticancer properties, as well as N-acylhydrazones, which exhibit anticancer action against many cell lines and molecular targets. The novel compounds are synthesised by sulphonating the indazole moiety at C-5, followed by the synthesis of Schiff bases from the parent 5-ethylsulfonyl-indazole-3-carbohydrazide, potentially increasing the antiproliferative activity and pharmacokinetics of the new **7a–o** derivatives.

All newly synthesised compounds were validated using ^1^H NMR,^13^C NMR, and elemental microanalysis. The newly synthesised compounds were evaluated for their safety profile against a normal cell line and for antiproliferative efficacy against four cancer cell lines. The most effective antiproliferative derivatives were then evaluated for their inhibitory activities against EGFR and VEGFR-2. Furthermore, the apoptotic efficiency of the most potent derivatives against Bax, p53, and Bcl-2 was evaluated. Finally, *in silico* investigations were performed to examine the binding interactions of the novel compounds with the selected receptors, as well as their pharmacokinetic properties.

## 2. Results and discussion

### 2.1. Chemistry

**Scheme 1.**
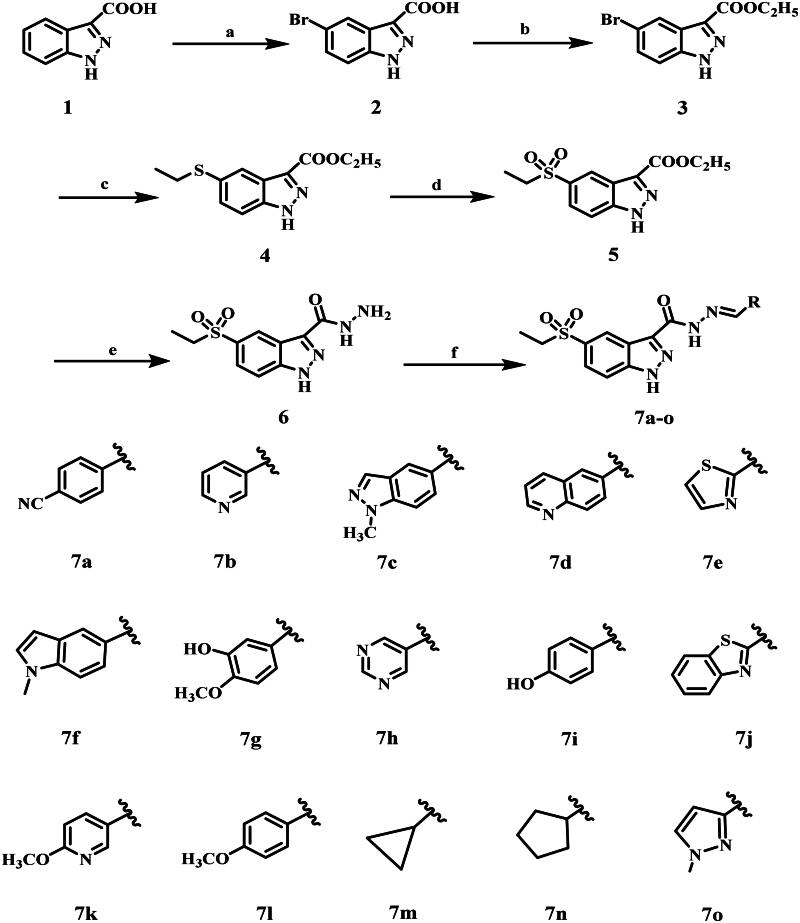
Synthesis of 5-(ethylsulfonyl)-1*H*-indazole-*N*-acyl hydrazones **7a–o**

The target compounds **7a–o** were synthesised following the synthetic route illustrated in Scheme 1. The synthesis commenced with the bromination of indazole-3-carboxylic acid **1**, which was obtained from Millipore Sigma and used without further purification. The reaction proceeded via an electrophilic aromatic substitution (SEAr) mechanism, yielding the 5-bromoindazole-3-carboxylic acid **2** as the major regioisomer. Subsequently, Fischer esterification of compound **2** was carried out by refluxing with anhydrous ethanol in the presence of concentrated sulphuric acid as a catalyst, affording intermediate **3**. The structure of compound **3** was confirmed by its ^1^H NMR spectrum, which exhibited characteristic triplet and quartette signals at δ 4.40 and δ 1.37 ppm, respectively, corresponding to the methylene and methyl protons of the ethyl ester group (Figure S3, Supplementary File).

The next step in this process involved a palladium-catalyzed C–S cross-coupling reaction under Buchwald–Hartwig conditions^45^, resulting in the introduction of a thioether functionality. Compound **4** was formed by reacting compound **3** with ethanethiol, utilising tris(dibenzylideneacetone) dipalladium(0) [Pd2(dba)3] as the palladium catalyst, Xantphos as the ligand, and *N*,*N*-diisopropylethylamine (DIPEA) as the base. The base’s role is to assist the reaction by deprotonating the thiol, either before or after coordination with the metal catalyst. Subsequent oxidation of thioether **4** using *m*-chloroperbenzoic acid (*m*-CPBA) afforded the corresponding sulphone intermediate **5**. LC–MS analysis confirmed the formation of the sulphonyl derivative rather than the sulphinyl derivative, supporting the complete oxidation of the sulphur centre. Moreover, the ^1^H NMR spectrum of sulphone derivative **5** exhibited the same number of signals as its precursor, indicating retention of the core structural framework. However, all aromatic proton signals exhibited a downfield shift, indicating an increased electron-withdrawing effect due to the presence of the sulphone functional group (Figure S7, Supplementary File).

In the next step, the ester compound **5** was refluxed with an excess of hydrazine hydrate (79%) in absolute ethanol, yielding the corresponding hydrazide derivative **6**. The ^1^H NMR spectrum of compound **6** confirmed the conversion, with the disappearance of the characteristic triplet and quartet signals of the ethyl ester group and the appearance of distinctive peaks at δ 5.47 and δ 4.84 ppm, corresponding to the –NH– and –NH_2_ protons of the hydrazide functionality (Figure S9, Supplementary File).

In the final step, the target molecules **7a–o** were obtained through the condensation of indazole-3-carbohydrazide derivative **6** with the appropriate aromatic/heteroaromatic aldehydes in ethanol containing a catalytic amount of acetic acid, yielding the corresponding *N*-acyl hydrazone derivatives **7a–o**. The final compounds’ structures were validated by ^1^H NMR and ^13^C NMR spectroscopy (Figures S11–S39, Supplementary File). The disappearance of the NH_2_ signal from the starting hydrazide and the development of a characteristic azomethine (N = CH) proton in the δ 7.93–7.81 ppm region provided convincing proof of successful condensation.

The ^1^H NMR spectrum of compound **7k**, as a representative example, displayed two singlet signals at δ 14.29 and 12.20 ppm, corresponding to the NH of indazole and carbohydrazide, respectively. The azomethine proton signal was detected at δ 7.92–7.83 ppm, accompanied by a single signal from the methoxy group at δ 3.86 ppm. The spectrum also indicated triplet and quartet ethyl group signals at δ 3.30 and 1.07 ppm (Figure S30, Supplementary File). The ^13^C NMR spectrum of **7k** revealed characteristic signals at δ 165.1, 158.5, and 54.1 ppm corresponding to the C-2 of pyrimidine, carbonyl group, and methoxy group (Figure S31, Supplementary File).

**Reagents and reactions conditions:** a) Br_2_, AcOH, 90 °C, 16 h; b) EtOH, H_2_SO_4_ (Cat), 90 °C, 16h; c) EtSH, Pd_2_(dba)_3_, Xantphos, DIPEA, Dioxane, 90 °C, 4h; d) *m*-CPBA, DCM, 0 °C to rt, 2h; e) NH_2_–NH_2_.H_2_O, EtOH, 90 °C, 6h; f) R-CHO, EtOH, AcOH (Cat), 90 °C.

### 2.2. Biology

#### 2.2.1. Cell viability assay

This test examines the effects of novel compounds **7a–o** on normal cell lines to assess their safety, a crucial factor in drug discovery. The viability of the investigated compounds was assessed using the normal human mammary gland epithelial cell line MCF-10A. After four days of incubation on MCF-10A cells with 50 µM of each examined compound, cell viability was assessed using the MTT test^46^^,^^47^. Table 1 results indicate that none of the tested compounds exhibited cytotoxicity, with all compounds maintaining cell viability above 91% at a concentration of 50 µM.

**Table 1.** Cell viability assay and IC_50_ values of compounds **7a–o** against four cancer cell lines.

Comp.	Cell viability %	Antiproliferative activity IC_50_ ± SEM (nM)				
		A-549	MCF-7	Panc-1	HT-29	Average IC_50_ (GI_50_)
**7a**	**94**	77 ± 6	79 ± 6	77 ± 6	78 ± 6	78
**7b**	**92**	47 ± 3	50 ± 4	48 ± 3	48 ± 3	48
**7c**	**91**	56 ± 4	59 ± 4	58 ± 4	58 ± 4	58
**7d**	**92**	53 ± 4	55 ± 4	54 ± 4	54 ± 4	54
**7e**	**94**	58 ± 4	61 ± 5	60 ± 5	60 ± 5	60
**7f**	**92**	71 ± 6	75 ± 6	73 ± 6	72 ± 6	73
**7 g**	**91**	35 ± 2	38 ± 2	36 ± 2	36 ± 2	36
**7h**	**93**	67 ± 5	70 ± 6	68 ± 5	68 ± 5	68
**7i**	**92**	31 ± 2	34 ± 2	32 ± 2	32 ± 2	32
**7j**	**91**	26 ± 1	28 ± 1	26 ± 1	27 ± 1	27
**7k**	**93**	24 ± 1	26 ± 1	25 ± 1	24 ± 1	25
**7 l**	**91**	41 ± 3	43 ± 3	42 ± 4	42 ± 4	42
**7 m**	**94**	49 ± 3	52 ± 4	50 ± 4	50 ± 4	50
**7n**	**90**	63 ± 5	66 ± 5	64 ± 5	64 ± 5	64
**7o**	**92**	29 ± 1	32 ± 1	29 ± 1	29 ± 1	30
**Erlotinib**	ND	30 ± 3	40 ± 3	30 ± 3	30 ± 3	33
						

**ND**: Not determined.

#### 2.2.2. Antiproliferative assay

The MTT assay was employed to assess the antiproliferative efficacy of novel compounds **7a–o** against four human cancer cell lines, using erlotinib as a reference: HT-29 (colon), Panc-1 (pancreatic), A-549 (lung), and MCF-7 (breast) cancer cell lines^48^^,^^49^. Table 1 presents the median inhibitory concentration (IC_50_) and GI_50_ (mean IC_50_) for the four cancer cell lines.

Table 1 shows that compounds **7a–o** had potent antiproliferative activity, with GI_50_ values ranging from 25 nM to 78 nM against the four cancer cell lines tested, in contrast to the reference erlotinib, which had a GI_50_ value of 33 nM. Compounds **7 g**, **7i–7l**, and **7o** emerged as the most efficient six derivatives, with GI_50_ values ranging from 25 nM to 42 nM. Notably, compounds **7j**, **7k**, and **7o** (GI_50_ values of 27, 25, and 30, respectively) exhibited marginally superior potency than erlotinib (GI_50_ value of 33 nM), particularly against breast cancer (MCF-7) cell lines.

Compound **7k** (Ar = 6-methoxypyridin-3-yl) exhibited the highest potency among the newly synthesised derivatives **7a–o**, demonstrating a GI_50_ value of 25 nM, which is 1.3-fold more effective than the reference erlotinib (GI_50_ = 33 nM). Compound **7k** exhibits greater potency than erlotinib against the four cancer cell lines tested. It had an IC_50_ value of 26 nM against the MCF-7 breast cancer cell line, demonstrating 1.5-fold more potency than erlotinib (IC_50_ = 40 nM).

According to the findings, the type of aryl (Ar) moiety found in the methylene group of the carbohydrazide moiety appears to be critical for action. For instance, compound **7 l** (Ar = 4-methoxyphenyl), which possesses the same backbone as compound **7k** but incorporates a phenyl group instead of a pyridine moiety, exhibited a GI_50_ of 42 nM (1.7-fold less potent than **7k**), indicating that the pyridine moiety is more crucial for antiproliferative activity than the phenyl one. Compound **7i** (Ar = 4-hydroxyphenyl) had a GI_50_ value of 32 nM against the four cancer cell lines studied, positioning it fourth in antiproliferative efficacy, in contrast to compound **7 l** (Ar = 4-methoxyphenyl), which demonstrated a GI_50_ value of 42 nM. The data indicate that when phenyl groups act as the aryl moiety, the hydroxyl group is preferred over the methoxy group.

Compound **7j** (Ar = benzothiazole-2-yl) ranked second in activity, with a GI_50_ value of 27 nM, comparable to that of compound **7k** (Ar = 6-methoxypyridin-3-yl), which exhibited a GI_50_ value of 25 nM. Compound **7j**, similar to compound **7k**, had more efficacy than the reference erlotinib across the four evaluated cancer cell lines. Compound **7e** (Ar = thiazol-2-yl) exhibited a GI_50_ value of 60 nM, demonstrating a potency that is 2.3-fold inferior to that of compound **7j** (Ar = benzothiazole-2-yl). The data highlight the significance of the phenyl group in the antiproliferative activity of compound **7j**.

Compound **7o** (Ar = 1-methylpyrazol-3-yl) ranked third in antiproliferative activity, exhibiting a GI_50_ of 30 nM, which is similar to that of the reference medication erlotinib (GI_50_ = 33 nM). Compound **7o** exhibits better activity than erlotinib against the MCF-7 breast cancer cell line, with an IC_50_ value of 32 nM, which is 1.25 times more potent than that of erlotinib (IC_50_ = 40 nM). Ultimately, compound **7a** (Ar = 4-cyanophenyl) had the lowest potency among compounds **7a–o**.

It demonstrated a GI_50_ value of 78 nM, which is 2.4-fold less potent than compound **7i** (Ar = 4-hydroxyphenyl), 1.9-fold less potent than compound **7 l** (Ar = 4-methoxyphenyl), and 2.1-fold less potent than compound **7 g** (Ar = 3-hydroxy-4-methoxyphenyl). These data demonstrate that when the phenyl group serves as the aryl moiety, it is preferable to have a hydroxyl group, a methoxy group, or both as substituents.

#### 2.2.3. Assay for EGFR inhibitory activity

The most effective antiproliferative compounds, **7 g**, **7i–7l**, and **7o**, were evaluated for their ability to inhibit EGFR using the EGFR-TK assay^50^^,^^51^. The results are presented in Table 2. Erlotinib served as the reference compound. The assay results align with those of the antiproliferative assay, indicating that compounds **7j**, **7k**, and **7o**, identified as the most potent antiproliferative agents, are the most effective derivatives of EGFR inhibitors, exhibiting IC_50_ values of 74 ± 4, 71 ± 4, and 76 ± 4, respectively. In every instance, compounds **7j**, **7k**, and **7o** exhibited superior efficacy as EGFR inhibitors compared to the reference erlotinib.

**Table 2.** IC_50_ values of compounds **7 g**, **7i–7l**, and **7o**, Erlotinib, and Sorafenib against EGFR and VEGFR-2.

Compound	EGFR inhibition IC_50_ ± SEM (nM)	VEGFR-2 inhibition IC_50_ ± SEM (nM)
**7g**	83 ± 5	39 ± 2
**7i**	81 ± 4	36 ± 2
**7j**	74 ± 4	27 ± 1
**7k**	71 ± 4	21 ± 1
**7 l**	87 ± 5	45 ± 3
**7o**	76 ± 4	31 ± 2
**Erlotinib**	80 ± 5	–
**Sorafenib**	–	0.17 ± 0.001

–: Not Determined.

Compound **7k** (Ar = 6-methoxy-pyridin-3-yl), the most effective antiproliferative compound, exhibited the highest potency as an EGFR inhibitor with an IC_50_ value of 71 nM, compared to erlotinib’s IC_50_ value of 80 nM. Compounds **7 g** and **7i** exhibited comparable EGFR inhibitory efficacy to erlotinib, with IC_50_ values of 81 and 83 nM, respectively. Ultimately, compound **7 l** exhibited significant activity as an EGFR inhibitor, with an IC_50_ value of 87 nM. The data indicate that compounds **7j**, **7k**, and **7o** are effective antiproliferative agents that may function as EGFR inhibitors.

#### 2.2.4. VEGFR-2 inhibitory assay

The inhibitory action of compounds **7 g**, **7i–7l**, and **7o** against VEGFR-2 was assessed using kinase assays, with sorafenib serving as the control agent^52^. Table 2 presents the results in terms of IC_50_ values. The findings revealed that the compounds studied effectively suppressed VEGFR-2, with IC_50_ values ranging from 21 to 45 nM, as opposed to sorafenib, which had an IC_50_ of 0.17 nM. In all cases, the compounds investigated were less potent than sorafenib as VEGFR-2 inhibitors, but more potent than EGFR inhibitors. Compounds **7j**, **7k**, and **7o**, recognised as the most efficacious antiproliferative and EGFR inhibitors, also exhibited significant potency as VEGFR-2 inhibitors with IC_50_ values of 27, 21, and 31 nM, suggesting their potential as dual EGFR/VEGFR-2 inhibitors.

#### 2.2.5. Apoptotic markers assays

Apoptosis, or programmed cell death, is a vital regulatory process that induces cell death when DNA damage surpasses the capacity of repair processes. Apoptosis, as a component of proper development, regulates cell abundance and proliferation^53^^,^^54^. Defects in apoptotic signalling contribute to a variety of human illnesses, including cancer. These flaws allow tumour cells to live longer than expected, reducing their reliance on exogenous survival factors and shielding them from oxidative stress and hypoxia, resulting in tumour growth and expansion. These abnormalities allow for the accumulation of genetic changes that promote angiogenesis, deregulating cell proliferation, interfering with differentiation, and increasing invasiveness as the tumour progresses^55^. Restoring normal apoptotic equilibrium is therefore a dependable cancer treatment approach.

Compounds **7j**, **7k**, and **7o** were evaluated for their ability to induce apoptosis in A-549 (lung) cancer cells by analysing the expression of key apoptotic markers, including Bcl-2, p53, and Bax. The results are cited in Table 3. The Bcl-2 protein family, comprising inducer proteins (Bax) and suppressor proteins (Bcl-2), primarily regulates apoptosis. Several studies have demonstrated a strong correlation between elevated Bcl-2 and reduced Bax levels, which are associated with tumour cell proliferation^56–59^. Consequently, we estimated the levels of Bcl-2 and Bax proteins in A-549 lung cancer cells treated with compounds **7j**, **7k**, and **7o**^60^. Table 3 presents results demonstrating a significant 9-fold increase in the Bax level and a 4.2-fold decrease in the Bcl-2 level for compound **7k** relative to the control untreated cells. Additionally, compounds **7j** and **7o** showed a significant 8-fold increase in Bax and a threefold decrease in Bcl-2 levels. The data indicate that apoptosis may contribute to the antiproliferative effects of the examined compounds.

**Table 3.** Results of apoptosis assays of compounds **7j**, **7k**, and **7o** against Bax, p53, and Bcl-2.

Compound No.	Bcl-2 (ng/mL)	Fold reduction	Bax (pg/mL)	Fold change	p53 (pg/mL)	Fold change
**7j**	1.50 ± 0.001	3.3	505 ± 3	8.5	340 ± 2	5.3
**7k**	1.20 ± 0.001	4.2	540 ± 3	9	371 ± 2	5.7
**7o**	1.80 ± 0.001	2.8	480 ± 2	8	285 ± 2	4.4
**Control**	5	1	60	1	65	1

A prime example of a gene product is the p53 protein, which affects apoptosis. The potential for p53 overexpression to induce apoptosis may elucidate the frequent deactivation of p53 enzymes by cancer cells during transformation^61^. The p53 levels in cancer cells treated with compounds **7j**, **7k**, and **7o** showed a significant increase, exceeding those of the untreated control cells by at least 5-fold. This observation suggests that elevated levels of the p53 protein regulate the apoptosis process in these novel compounds.

### 2.3. Computational studies

#### 2.3.1. Molecular mechanical computations

Molecular mechanics (MM) is a computational chemistry method based on classical physics principles, employed to investigate molecular structures, energetics, and interactions. MM calculations predict the geometry, energy profiles, and intermolecular interactions of molecules^62^. This technique is integral to molecular docking and molecular dynamics (MD) simulations, significantly contributing to the exploration of ligand–receptor interactions, ligand binding affinity, conformational stability, and dynamic behavior^63^. In molecular docking studies, MM-based scoring functions provide quantitative estimates of ligand–protein binding affinities, identify preferred binding orientations, and highlight critical interactions within the receptor’s active sites. Similarly, MD simulations utilise MM to explore molecular flexibility and stability over extended simulation periods, providing in-depth insights into conformational variations and dynamic ligand–receptor interactions^64^. The integration of MM calculations with molecular docking and MD simulations enables an enhanced and comprehensive evaluation of ligand binding behaviour, molecular recognition, and stability within the receptor binding site. Such integrative computational analyses are instrumental in the rational design, optimisation, and refinement of novel anticancer candidates, thereby improving drug discovery efficiency^65^.

#### 2.3.2. Molecular docking studies of EGFR and VEGFR-2 enzymes

**Figure 6.**
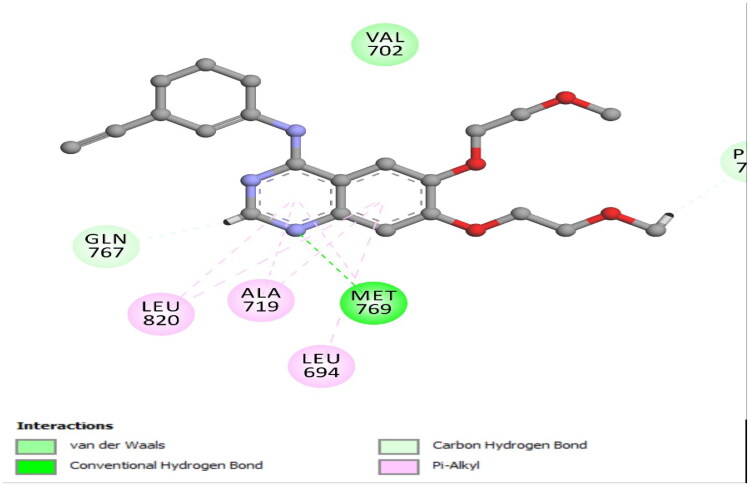
Validation docking pose of the co-ligand erlotinib within the EGFR active site (PDB ID: 1M17).

Molecular docking analyses were performed to investigate the binding modes and detailed molecular interactions of our newly synthesised indazole-based derivatives **(7i, 7k,** and **7 l)** against EGFR (PDB ID: 1M17) and VEGFR-2 (PDB ID: 3WZE)^44^. Erlotinib and sorafenib were utilised as reference ligands for EGFR and VEGFR-2 enzymes, respectively. The crystal structures of EGFR (PDB ID: 1M17) and VEGFR-2 (PDB ID: 3WZE) were obtained from the Protein Data Bank^66^. Protein preparation was performed using Discovery Studio 2016, version 16.1.0.15 (BIOVIA, Dassault Systèmes) with the CDOCKER module, which is based on a CHARMM algorithm. All heteroatoms, ligands, and crystallographic water molecules beyond 5 Å from the binding site were removed. Missing hydrogen atoms were added, and the protonation states of ionisable amino acid residues were adjusted to pH 7.4 using the built-in pKa estimation tools. Histidine tautomeric forms were manually checked and optimised for hydrogen bonding capability^67^. The prepared structures were then subjected to energy minimisation using the CHARMm force field, applying a root mean square gradient convergence criterion of 0.01 kcal/mol/Å to relieve steric clashes while preserving the backbone geometry^68^. These minimised structures were used for subsequent molecular docking and dynamics studies. Rigid receptor–flexible ligand docking was employed. The receptor (EGFR: PDB ID 1M17; VEGFR-2: PDB ID 3WZE) was kept rigid during the docking process, while full conformational flexibility was allowed for the ligands. Ligands were prepared using the “Prepare Ligands” protocol in Discovery Studio, which included the generation of low-energy 3D conformers and assignment of protonation states. The active site was defined based on the coordinates of the co-crystallized ligand. This site encompasses key residues known to participate in kinase–inhibitor interactions, as reported in structural and literature data^69^^,^^70^. No blind docking was performed. For each ligand, 10 poses were generated, and the best pose was selected based on the CDOCKER interaction energy score (a combination of van der Waals and electrostatic energy terms), which is reported in kcal/mol. Additional visualisation and analysis of receptor–ligand interactions (hydrogen bonding, pi–pi stacking, and hydrophobic contacts) were performed using the “Receptor–Ligand Interactions” tools in Discovery Studio. The docking protocol was validated by re-docking the co-crystallized ligand into its native binding site, yielding an RMSD < 2.0 Å. The re-docked poses were compared with their experimental crystallographic conformations using root mean square deviation (RMSD) analysis. This validation yielded an *S* score of −7.68 kcal/mol and an RMSD of 1.04 Å. These results demonstrate that the docking setup can faithfully reproduce experimentally observed ligand binding orientations and is suitable for predictive modelling. Moreover, the critical hydrogen bond interaction between pyrimidine nitrogen and the hinge-region residue Met769 was confirmed, indicating its pivotal role in stabilising ligand binding within the EGFR active site (Figure 6).

Docking results were evaluated using a combination of quantitative and qualitative criteria. The primary selection metric was the interaction energy score, which accounts for van der Waals and electrostatic interactions between the ligand and receptor. Among the generated poses, those with the lowest (most negative) interaction energy were prioritised.

**Figure 7.**
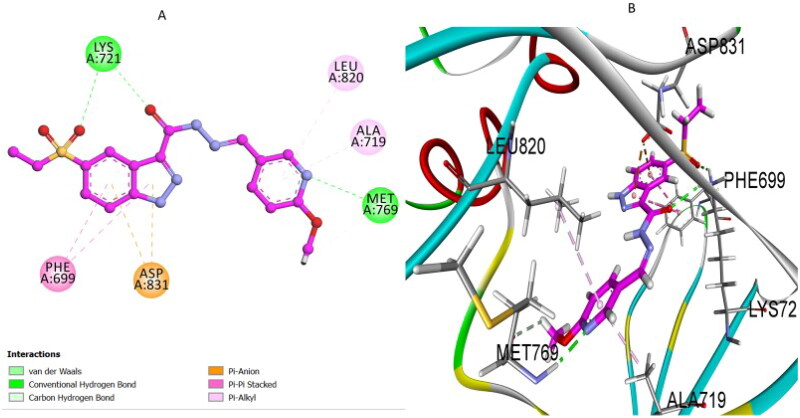
(A) 2D and (B) 3D docking poses illustrating the interactions of compound **7k** in the EGFR active site.

Additionally, each top-scoring pose was visually inspected to assess the orientation of the ligand within the binding pocket, ensuring correct positioning relative to key active-site residues. Ligand–receptor interactions, including hydrogen bonds, pi–stacking, pi–cation, and hydrophobic contacts, were analysed. Only poses exhibiting strong interaction profiles and consistent alignment were selected for further analysis. Among the tested derivatives, compound **7k** displayed the highest docking affinity with an S score of −8.25 kcal/mol and an RMSD of 0.8 Å, aligning closely with its superior experimental EGFR inhibitory activity (IC_50_ = 71 nM). Analysis of its docking pose (Figure 7) revealed multiple interactions underlying its remarkable affinity. A conventional hydrogen bond between the nitrogen atom of its pyridine moiety and the hinge residue Met769 closely mimicked the essential interaction observed with erlotinib, ensuring optimal orientation and stability within the binding pocket. Additionally, compound **7k** established a critical hydrogen bond interaction involving the carbonyl oxygen atoms of its hydrazide and sulphonyl functional groups with the side chain of residue Lys721, significantly enhancing ligand–receptor binding stability. Furthermore, significant pi–anion interactions with Asp831, and robust pi–alkyl interactions involving residues Leu820, Ala719, and Phe699 were observed with the indazole ring, complementing the hydrogen bonding interactions. These cumulative interactions effectively anchored compound **7k** within the active site, consistent with its experimentally demonstrated potent EGFR inhibitory activity (IC_50_ = 71 nM) and excellent antiproliferative efficacy.

**Figure 8.**
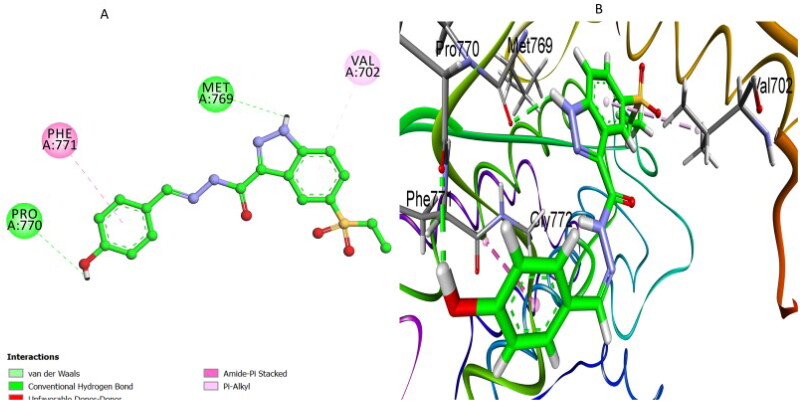
(A) 2D and (B) 3D interaction representations of **7i**, highlighting the hydrogen bond between its phenolic OH group and residue Pro770, as well as supplementary hydrophobic interactions with Val702 and pi-stacking with residue Phe771.

Compound **7i**, which exhibited an intermediate binding affinity (*S* score = −7.42 kcal/mol; RMSD = 1.39 Å), replaced the pyridine moiety with a phenolic hydroxyl group, thereby forming a critical conventional hydrogen bond interaction with residue Pro770. This hydrogen bond provided notable stabilisation within the active site, although it positioned compound **7i** slightly offset compared to compound **7k** (Figure 8). Another significant conventional hydrogen bond formed between the nitrogen of the indazole ring and residue Met769 additionally reinforced compound stabilisation. Moreover, the phenyl rings of compound **7i** established essential hydrophobic pi–alkyl interactions with residue Val702 and valuable pi–stacked interactions with Phe771. While compound **7i** maintained these crucial contacts, it exhibited fewer overall interactions compared to compound **7k**, which explains its moderate EGFR inhibitory activity experimentally (IC_50_ = 81 nM).

**Figure 9.**
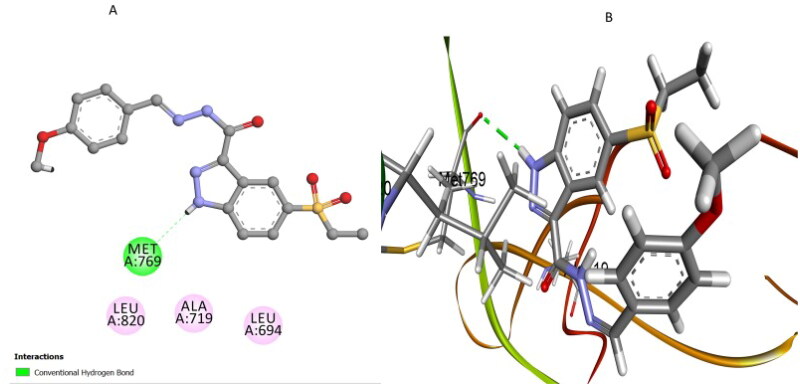
(A) 2D and (B) 3D docking representations of **7 l**, highlighting the limited molecular interactions observed. The presence of only one significant hydrogen bond with Met769 accounts for its low binding affinity (S score = −6.82 kcal/mol, RMSD = 1.66 Å).

Compound **7 l** exhibited the lowest binding affinity (*S* score = −6.82 kcal/mol; RMSD = 1.66 Å), supported by fewer and weaker interactions (Figure 9). Unlike compounds **7k** and **7i**, compound **7 l** lacked the pyridine nitrogen or phenolic OH group crucial for forming robust hydrogen bonds. Although compound **7 l** formed a single conventional hydrogen bond between indazole nitrogen and residue Met769, analogous to compounds **7i** and **7k**, this sole interaction was insufficient for robust stabilisation. Critically, the absence of additional hydrogen bonds with Pro770 or Lys721 resulted in a markedly reduced docking affinity (*S* score = −6.82 kcal/mol, RMSD = 1.66 Å), which corresponds clearly to its weakest experimental EGFR inhibition potency (IC_50_ = 87 nM).

Overall, these docking analyses align closely with the experimental *in vitro* data on EGFR inhibition. They emphasise the fundamental role of the pyridine nitrogen atom in compound **7k** and the phenolic hydroxyl group in compound **7i**, which are crucial for establishing robust hydrogen bonding interactions. Such insights confirm the structure–activity relationships obtained experimentally and offer valuable guidance for designing future EGFR-targeted anticancer therapeutics with optimised potency and specificity.

**Figure 10.**
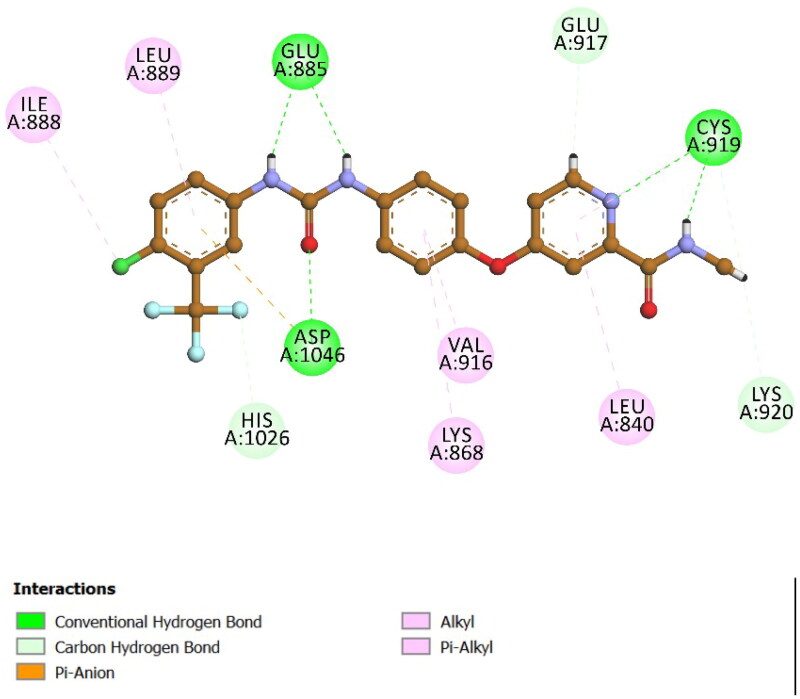
Docking pose and 2D interaction diagram of co-ligand sorafenib within the VEGFR-2 active site (PDB ID: 3WZE).

Docking studies against VEGFR-2 were also performed to gain further insights into the molecular interactions of our compounds within the VEGFR-2 active site. Sorafenib, an established VEGFR-2 inhibitor, was used as the reference ligand. Docking of sorafenib validated the accuracy of our computational approach, yielding an S score of −8.71 kcal/mol and an RMSD value of 0.75 Å, which indicates a precise and energetically favourable ligand–protein complex. A detailed analysis of the binding pose (Figure 10) revealed extensive hydrogen bonding interactions, which are critical for its strong affinity towards VEGFR-2. The key hydrogen bond interactions involved residues Glu885, Cys919, and Asp1046. Furthermore, sorafenib displayed multiple hydrophobic interactions (pi–alkyl and alkyl interactions) with residues such as Val916, Leu840, Lys868, and Leu889, accompanied by a stabilising pi–anion interaction with Asp1046. Collectively, these interactions substantially contributed to the strong binding stability and potent inhibitory activity exhibited by sorafenib.

**Figure 11.**
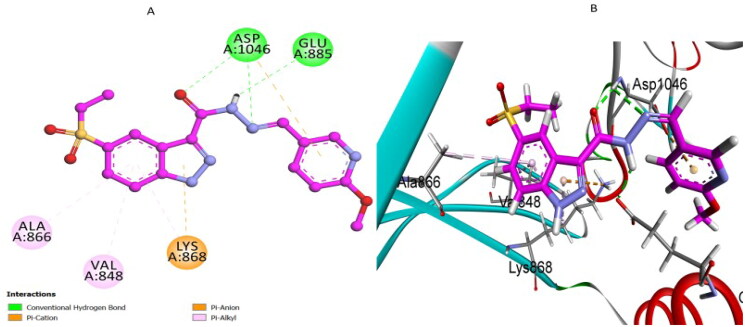
(A) 2D and (B) 3D representations of the docking interactions for compound **7k** in the VEGFR-2 active site.

Compound **7k**, our leading derivative, exhibited significant inhibitory potency experimentally against VEGFR-2 (IC_50_ = 21 nM) and correspondingly demonstrated substantial docking affinity (S score = −7.12 kcal/mol, RMSD = 1.57 Å). The docking analysis of compound **7k** within the VEGFR-2 active site (Figure 11) revealed a strong molecular interaction profile that closely aligns with the key interactions of sorafenib. Compound **7k** formed conventional hydrogen bonds with essential catalytic residues, specifically residues Glu885 and Asp1046, similar to interactions observed with sorafenib. These hydrogen bonds significantly stabilised compound **7k** within the enzyme active site, promoting a strong binding affinity. In addition to these critical hydrogen bonds, compound **7k** exhibited extensive hydrophobic π–alkyl and π–anion interactions with residues Val848, Ala866, and Lys868, further enhancing the stabilisation of its ligand–protein complex. Notably, compound **7k** also established a valuable pi–anion interaction with the residue Asp1046, analogous to the interaction observed with sorafenib, providing an additional layer of electrostatic stabilisation. This comprehensive interaction network rationalises its potent VEGFR-2 inhibition, as confirmed by experimental enzyme assays.

In summary, docking studies against VEGFR-2 reinforced the experimental biological activity observed for compound **7k**, providing critical structural insights into its interaction pattern. Compound **7k** effectively mimics key binding interactions displayed by sorafenib, particularly the pivotal hydrogen bonding and pi–anion interactions with Glu885 and Asp1046 residues, emphasising its strong potential as a dual-targeted EGFR/VEGFR-2 anticancer agent.

#### 2.3.3. Molecular dynamics (MD) simulations for compound 11 and erlotinib with EGFR

**Figure 12.**
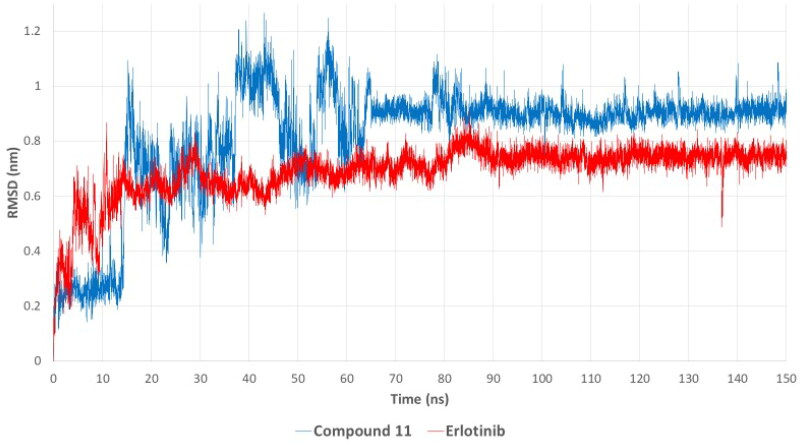
RMSD plot showing the structural stability of the compound **7k** –EGFR and erlotinib–EGFR complexes over 150 ns of molecular dynamics simulation. Compound **7k** exhibited a stable RMSD of ∼0.8 nm, while erlotinib showed a slightly lower and more rigid binding conformation (RMSD ∼0.6 nm).

To gain deeper insights into the binding stability and dynamic behaviour of compound **7k** in complex with EGFR, molecular dynamics (MD) simulations were conducted for 150 ns, with erlotinib used as a reference ligand^71^. Molecular dynamics (MD) simulations were performed using GROMACS 2023 to evaluate the stability and binding behaviour of the EGFR–compound **7k** complex^72^. The protein–ligand complex was prepared using UCSF Chimaera, where hydrogen atoms were added to maintain correct geometry and bonding^73^. The CHARMM36 force field was applied to the protein, and ligand parameters were generated using the CHARMM General Force Field (CGenFF)^74^^,^^75^. Ligand topology and parameters were generated using the CGenFF web application (ParamChem server) to ensure compatibility with the CHARMM36 force field used for the protein^76^^,^^78^. Following parameter generation, penalty scores were reviewed to assess the reliability of assigned bonded and electrostatic terms. All relevant parameters exhibited penalty scores below 10, indicating high confidence in the force field analogy and no need for reparameterization. To validate structural integrity, energy minimisation and short equilibration runs were performed using GROMACS 2023, followed by visual inspection of the ligand within the protein–ligand complex. No abnormalities were observed, and no manual corrections were required. Each protein–ligand complex was embedded in a periodic cubic simulation box solvated with TIP3P water molecules, maintaining a 1 nm buffer around all sides of the complex^79^. To neutralise the system and mimic physiological ionic strength, Na^+^ and Cl^–^ ions were added at a final concentration of 150 mM. Energy minimisation was performed using the steepest descent algorithm to eliminate steric clashes and optimise the system. NVT equilibration for 100 ps at 300 K using the V-rescale thermostat, maintaining a constant volume^80^. NPT equilibration for 100 ps at 1.0 bar using the Parrinello–Rahman barostat, allowing pressure equilibration^81^. During both equilibration phases, position restraints were applied to the heavy atoms of the protein–ligand complex. The production MD run was then performed for 150 ns without restraints, using a 2 fs time step. Trajectory frames were saved every 10 ps for subsequent analysis. Periodic boundary conditions were applied in all directions. Bond lengths involving hydrogen atoms were constrained using the LINCS algorithm^82^, and long-range electrostatic interactions were calculated using the Particle Mesh Ewald (PME) method with a 10 Å cutoff^83^. Trajectory snapshots were saved every 10 ps for post-simulation analysis. To evaluate the stability and dynamics of the complexes, several key parameters were analysed. Root Mean Square Deviation (RMSD) was calculated to monitor the global conformational stability of the protein–ligand complex throughout the simulation. Root Mean Square Fluctuation (RMSF) was used to assess the flexibility of individual amino acid residues. The radius of gyration (Rg) was computed to measure the overall compactness of the protein structure. In addition, hydrogen bond analysis was conducted to quantify the number and persistence of intermolecular hydrogen bonds between the protein and ligand. Finally, the potential energy profile of the system was monitored across the 150 ns simulation to confirm thermodynamic stability. The RMSD (Root Mean Square Deviation), hydrogen bond occupancy, and potential energy profiles were analysed to evaluate the structural stability and interaction strength of the ligand–protein complexes throughout the simulation. The RMSD plot (Figure 12) illustrates the stability of compound **7k** and erlotinib within the EGFR binding pocket over time. Initially, both complexes exhibited fluctuations during the first 20–30 ns as the ligands adapted to the binding site. However, after approximately 70 ns, compound **7k** stabilised with an RMSD value averaging 0.8 nm, indicating a stable binding conformation for the remainder of the simulation. In contrast, erlotinib achieved equilibrium earlier (around 20 ns) and maintained a lower RMSD (∼0.6 nm), suggesting a more rigid binding pose within the active site. Despite the slightly higher RMSD of compound **7k**, it remained within an acceptable range, indicating that it maintained a stable and persistent interaction with EGFR throughout the simulation. The higher RMSD fluctuations observed for compound **7k**, particularly between 30 and 60 ns, may be attributed to conformational rearrangements within the binding site, allowing the ligand to establish a more optimised interaction network. This behaviour is not uncommon for flexible molecules, as their initial binding conformation may undergo subtle refinements before reaching a final, stable state.

**Figure 13.**
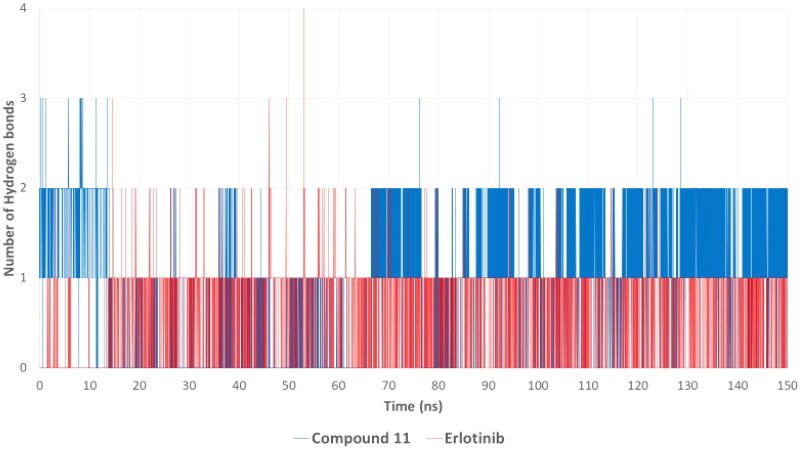
Hydrogen bond occupancy plot illustrating the number of hydrogen bonds formed between EGFR and compound **7k** or erlotinib throughout the simulation. Compound **7k** exhibited one to three hydrogen bonds, while erlotinib predominantly maintained a single hydrogen bond, suggesting a more dynamic hydrogen bonding pattern for compound **7k**.

Hydrogen bonding is a critical factor influencing the stability and specificity of ligand–protein interactions. The hydrogen bond occupancy analysis (Figure 13) revealed that compound **7k** consistently formed one to three hydrogen bonds with key residues in the EGFR active site, with at least one hydrogen bond maintained throughout the simulation. Notably, compound **7k** displayed a more dynamic hydrogen bond network, suggesting strong yet flexible interactions that contribute to its high affinity. In contrast, erlotinib formed fewer hydrogen bonds, with a predominant single hydrogen bond observed during most of the simulation time. This suggests that while erlotinib binds strongly to EGFR, it does so with a more limited hydrogen bonding network, potentially relying more on hydrophobic and van der Waals interactions for stability. The ability of compound **7k** to form multiple hydrogen bonds, including interactions with Met769 and Lys721, aligns with its superior EGFR inhibitory activity (IC_50_ = 71 nM) compared to erlotinib (IC_50_ = 80 nM).

To further evaluate the dynamic behaviour and stability of the EGFR complexes with compound **7k** and erlotinib, we performed root mean square fluctuation (RMSF) and radius of gyration (Rg) analyses over the 150 ns molecular dynamics simulations.

**Figure 14.**
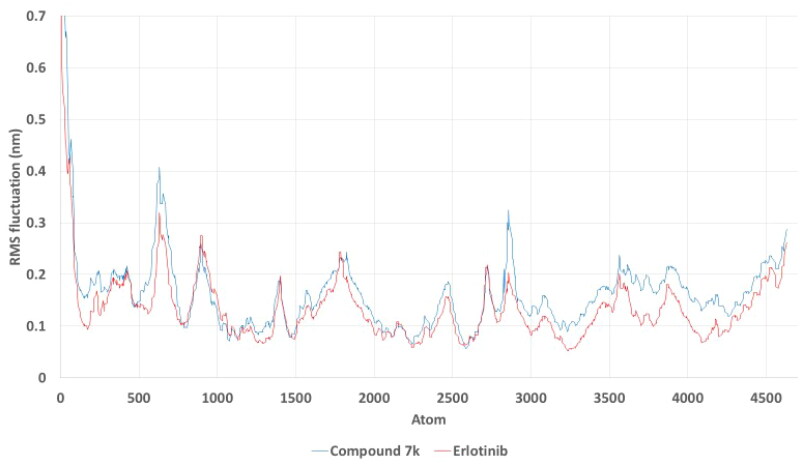
Root mean square fluctuation (RMSF) analysis of the EGFR complexes with compound 7k (blue) and erlotinib (red) over a 150 ns molecular dynamics simulation. The plot shows the flexibility of individual amino acid residues, with slightly higher fluctuations observed in certain regions for the compound 7k complex compared to erlotinib, indicating differences in local residue mobility.

The RMSF analysis (Figure 14) reveals the flexibility of individual residues within the protein during the simulation. Both complexes exhibited similar fluctuation profiles across most residues, with compound **7k** showing slightly higher RMSF values than erlotinib in certain regions, indicating increased local flexibility. This suggests that while compound **7k** maintains stable binding overall, it may allow more conformational adaptability within the binding site compared to erlotinib. Such flexibility could be advantageous for accommodating induced-fit interactions or multiple binding modes.

**Figure 15.**
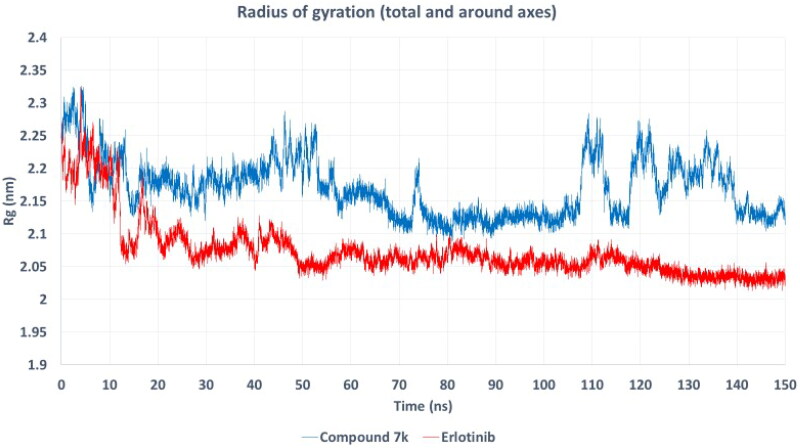
Radius of gyration (*R*g) profiles for EGFR complexes bound to compound 7k (blue) and erlotinib (red) during 150 ns molecular dynamics simulations. The *R*g values reflect the overall compactness and folding stability of the protein. The erlotinib complex exhibits a consistently lower *R*g, suggesting a more compact conformation, while the compound 7k complex maintains stable yet slightly higher *R*g values indicative of minor conformational flexibility.

The radius of gyration (*R*g) profiles (Figure 15) demonstrate the overall compactness and folding stability of the protein during simulation. The *R*g values for both complexes remain relatively stable throughout, with erlotinib-bound EGFR consistently exhibiting a slightly lower *R*g (∼2.05 nm) compared to the compound **7k** complex (∼2.15–2.20 nm). This indicates that the erlotinib complex adopts a more compact and rigid conformation, whereas compound 7k-bound EGFR shows marginally higher structural fluctuations. However, both systems maintain their structural integrity without significant unfolding or expansion.

**Figure 16.**
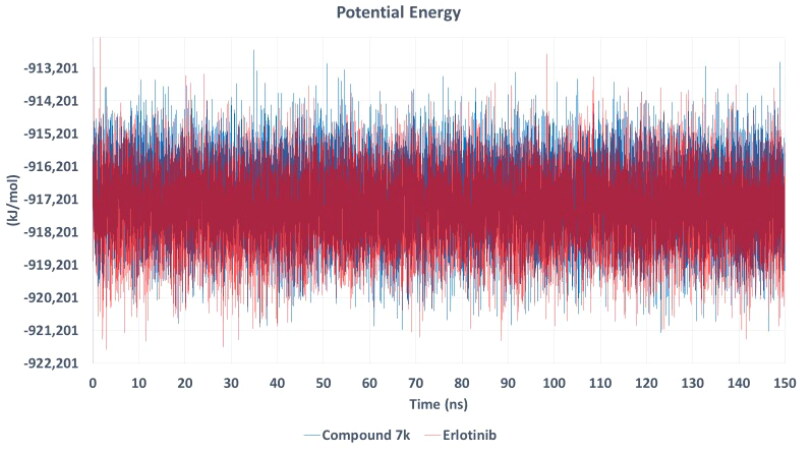
Potential energy profile of **7k**–EGFR and erlotinib–EGFR complexes over 150 ns. Both complexes remained energetically stable, confirming the thermodynamic favorability of ligand binding. Compound **7k** showed slightly higher fluctuations, indicative of conformational flexibility and adaptability within the EGFR active site.

The potential energy profile (Figure 16) provides further evidence supporting the stability of both ligand–EGFR complexes. The potential energy values for both compound **7k** and erlotinib remained consistent throughout the 150 ns simulation, indicating that both complexes were energetically favourable and stable. Minor fluctuations were observed, but no significant destabilisation events occurred, reinforcing the validity of the docking predictions. Although the compound **7k**–EGFR complex exhibited slightly higher energy fluctuations, this is consistent with its more flexible hydrogen bonding pattern and larger conformational adaptability, which may contribute to its enhanced inhibitory potency. On the other hand, erlotinib exhibited a slightly lower and more stable energy profile, correlating with its rigid binding conformation.

The combined analyses illustrate that while erlotinib forms a more rigid and compact complex with EGFR, compound **7k** maintains a stable but more flexible binding mode. This flexibility may allow compound 7k to adapt to conformational changes in the receptor, potentially contributing to unique binding characteristics and efficacy profiles. The stability in potential energy and persistent hydrogen bonding support the overall robustness of both complexes.

The MD simulation results strongly support the molecular docking findings, providing additional evidence of the stability of **7k** and favourable interaction profile within the EGFR active site. These findings are consistent with the experimental EGFR inhibitory data, where compound **7k** exhibited superior inhibition (IC_50_ = 71 nM) compared to erlotinib (IC_50_ = 80 nM).

#### 2.3.4. Quantum mechanical (QM) computations for compound 7k

Quantum mechanical (QM) computations offer a deeper understanding of the electronic structure, reactivity, and binding potential of bioactive molecules, thereby explaining their inhibitory effects at the molecular level^84^. In this study, Density Functional Theory (DFT) and Molecular Electrostatic Potential (MEP) mapping were employed to analyse the electronic properties of **7k**, a potent EGFR and VEGFR-2 inhibitor, to establish a direct correlation between its electronic behaviour, molecular docking, molecular dynamics (MD) simulations, and experimental inhibitory activity^85^.

#### 2.3.5. Density functional theory (DFT) analysis of compound 7k

**Figure 17.**
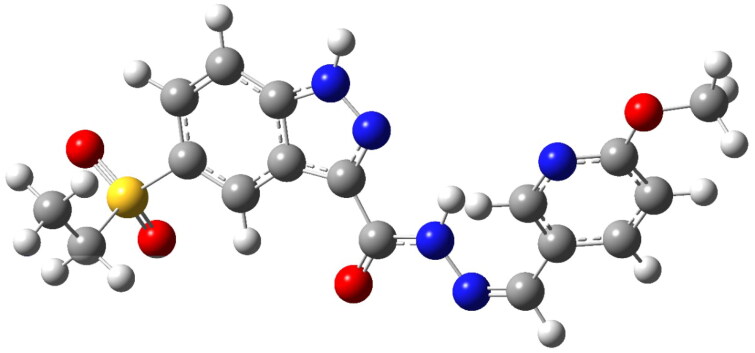
Optimised molecular geometry of **7k**, obtained from DFT calculations using the 6–311 + G(2d,p) basis set.

To evaluate the electronic features and stability of **7k**, DFT calculations were performed using the 6–311 + G(2d,p) basis set^86^. This hybrid DFT method was chosen for its well-documented performance in modelling the structural and electronic characteristics of organic molecules, particularly heterocyclic systems^87^^,^^88^. The basis set includes diffuse and polarisation functions, which improve the accuracy of frontier orbital predictions, while maintaining computational feasibility for drug-like compounds^89^. The optimised molecular geometry was confirmed as a global minimum through frequency analysis, ensuring its stability and suitability for further computational assessments^62^ (Figure 17).

**Figure 18.**
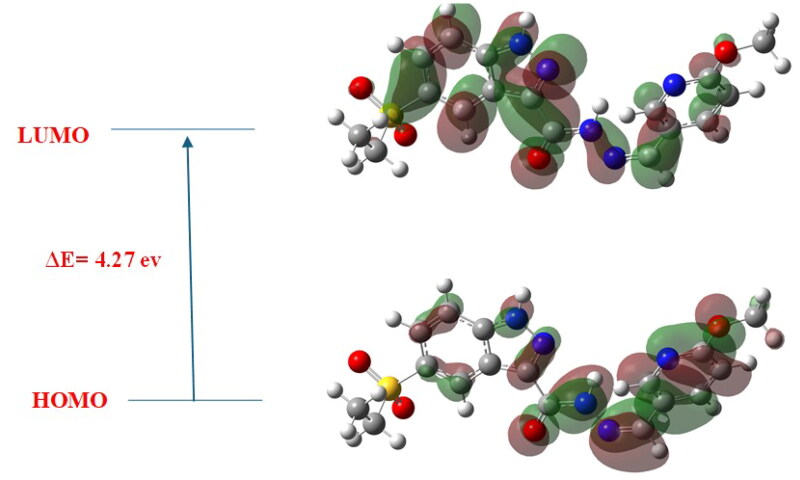
HOMO–LUMO energy gap (Δ*E* = 4.27 eV) of **7k**, illustrating electronic reactivity and stability. HOMO (lower panel) is localised over the pyridine and carbohydrazide moieties, in contrast, LUMO (upper panel) is mainly distributed over the indazole core and sulphonyl group, reflecting a balanced electronic profile conducive to effective receptor binding interactions.

In addition to geometry optimisation, frontier molecular orbitals (HOMO and LUMO) were calculated using the B3LYP/6–311 + G(2d,p) level of theory. The HOMO–LUMO energy gap was used to estimate the chemical reactivity and kinetic stability of the compounds^90^. A smaller energy gap typically indicates greater reactivity and potential for electron transfer interactions, which are critical for binding affinity^91^. The electronic distributions of HOMO and LUMO were also visualised to identify potential sites of nucleophilic and electrophilic activity within each molecule^92^. To address concerns regarding the chemical stability of the imine (Schiff base) scaffold, we conducted a comprehensive analysis of the frontier molecular orbitals (FMOs) of compound 7k. The calculated HOMO–LUMO energy gap is 4.27 eV (Figure 18), with a chemical hardness (η) of 2.135 eV and a softness (σ) of 0.234 eV^−1^. These parameters suggest a favourable balance between stability and reactivity. Recent studies have demonstrated that a larger HOMO–LUMO gap correlates with increased kinetic stability and decreased chemical reactivity^48^^,^^93^. For instance, compounds with higher energy gaps exhibit reduced susceptibility to electron transfer reactions, enhancing their stability under physiological conditions^94^. Additionally, the chemical hardness and softness values provide insights into the resistance to deformation and its polarizability, respectively^95^. A higher hardness value indicates a more stable and less reactive molecule, while a lower softness value suggests reduced polarizability^96^. Furthermore, the stability of Schiff base derivatives has been corroborated through density functional theory (DFT) studies, which reveal that such compounds can maintain structural integrity and exhibit desirable electronic properties. These findings support the viability of the imine scaffold in drug design, particularly when electronic parameters indicate favourable stability profiles. The HOMO (Highest Occupied Molecular Orbital) distribution was found to be predominantly localised over the pyridine and carbohydrazide moieties, indicating that these regions act as electron donors, contributing to π–π stacking and hydrogen bonding interactions within the EGFR and VEGFR-2 active sites. In contrast, the LUMO (Lowest Unoccupied Molecular Orbital) orbital distribution was primarily observed over the indazole core and sulphonyl group, identifying these regions as electron acceptors that facilitate key electrostatic and hydrogen bond interactions with receptor residues. This electronic distribution aligns well with molecular docking findings, where the pyridine nitrogen interacts with Met769 in EGFR, and the carbohydrazide moiety contributes to binding with Asp1046 and Glu885 in VEGFR-2.

#### 2.3.6. Molecular electrostatic potential (MEP) analysis

**Figure 19.**
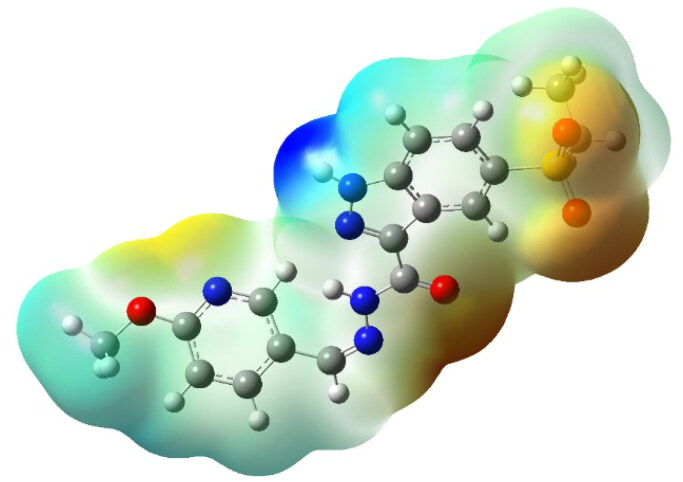
Molecular electrostatic potential (MEP) surface of **7k**, highlighting charge distribution. Red and orange regions correspond to electron-rich (nucleophilic) areas, primarily sulphonyl and carbonyl oxygens, as well as pyridine nitrogen, which serves as a hydrogen bond acceptor in protein binding interactions. Blue regions indicate electron-deficient (electrophilic) sites, localised around amide hydrogen atoms, which act as hydrogen bond donors and support ligand–protein stabilisation within the active sites of EGFR and VEGFR-2.

The Molecular Electrostatic Potential (MEP) map of **7k** (Figure 19) provides a detailed visualisation of charge distribution, identifying key electrophilic and nucleophilic regions critical for ligand–protein interactions. The electron-rich (nucleophilic) regions, primarily localised around the oxygen atoms of the sulphonyl and carbohydrazide groups, as well as the pyridine nitrogen, serve as hydrogen bond acceptor sites. This observation is consistent with docking, where these functional groups formed stable hydrogen bonds with Met769 and Lys721 in EGFR, and Asp1046 and Glu885 in VEGFR-2. Conversely, the electron-deficient (electrophilic) regions, predominantly observed around the hydrogen atoms of the carbohydrazide moiety, function as hydrogen bond donor sites, facilitating key interactions with receptor residues. This charge distribution strongly supports the hydrogen bonding interactions identified in MD simulations, where **7k** maintained persistent hydrogen bonds throughout the 150 ns simulation period, reinforcing its high binding affinity and stability.

The DFT and MEP findings, in conjunction with docking, MD, and experimental results, provide strong evidence for the high potency of **7k** as a dual EGFR/VEGFR-2 inhibitor. The localised HOMO and LUMO regions, along with distinct nucleophilic and electrophilic sites, rationalise their strong binding affinity, stable receptor interactions, and potent enzymatic inhibition. These insights reinforce compound **7k** as a promising anticancer candidate, offering a strong foundation for future structure-based drug design and optimisation efforts.

### 2.4. ADME studies

To assess the pharmacokinetic and drug-likeness properties of **7k** and erlotinib, an ADME (Absorption, Distribution, Metabolism, and Excretion) study was conducted using SwissADME. This comparative analysis offers insights into the physicochemical, solubility, pharmacokinetic, and medicinal chemistry profiles of both molecules, providing a comprehensive evaluation of their potential as EGFR inhibitors.

Compound **7k** has a molecular weight of 387.41 g/mol, while erlotinib has a slightly higher molecular weight of 393.44 g/mol. Both compounds meet Lipinski’s rule of five, indicating favourable oral bioavailability. Erlotinib has 16 aromatic heavy atoms, compared to 15 in **7k**, suggesting a similar structural complexity and potential for π-stacking interactions. Topological Polar Surface Area (TPSA) is a crucial factor influencing cell permeability. Compound **7k** has a higher TPSA (134.78 Å^2^) compared to erlotinib (74.73 Å^2^), suggesting that **7k** may have slightly lower membrane permeability but stronger hydrogen bonding potential, as evidenced in docking and MD simulations. Lipophilicity, represented as log Po/w, affects membrane permeability and drug distribution.

The consensus log Po/w of **7k** is 1.70, whereas erlotinib has a significantly higher log Po/w of 3.20, indicating that erlotinib is more lipophilic. This increased lipophilicity suggests that erlotinib may penetrate cell membranes more efficiently; however, **7k** may offer a better balance between solubility and permeability, thereby reducing non-specific tissue accumulation. In terms of water solubility, **7k** is moderately soluble (log S = −3.22, 0.231 mg/mL), whereas erlotinib is moderate to poorly soluble, with a log S range from −4.11 to −7.26, indicating lower aqueous solubility. This suggests that **7k** may have a better solubility profile, supporting its potential for improved bioavailability in physiological environments. Both compounds exhibit high gastrointestinal (GI) absorption, indicating strong oral bioavailability. However, a key difference is that erlotinib crosses the blood–brain barrier (BBB), while **7k** does not. This suggests that compound **7k** is less likely to cause central nervous system (CNS) side effects, making it a safer alternative for targeted therapy. A critical distinction between the two compounds lies in their effects on the cytochrome P450 (CYP) enzyme, which influences drug metabolism and the potential for drug–drug interactions.

Erlotinib inhibits CYP1A2, CYP2C19, CYP2C9, CYP2D6, and CYP3A4, which raises concerns about metabolic interactions and toxicity. In contrast, compound **7k** does not inhibit these enzymes, reducing the likelihood of adverse metabolic interactions. Additionally, compound **7k** is not a substrate for P-glycoprotein (P-gp), indicating a lower probability of efflux-mediated resistance compared to erlotinib, which may be prone to P-gp-related drug resistance mechanisms. Both compounds satisfy Lipinski’s, Ghose’s, Veber’s, and Muegge’s drug-likeness filters, suggesting strong oral bioavailability and drug-like characteristics. However, erlotinib has two lead-likeness violations (MW > 350, rotatable bonds >7), while **7k** has only one (MW > 350), indicating that **7k** may be a more synthetically accessible and chemically favourable lead candidate.

The synthetic accessibility score for **7k** is 3.07, whereas for erlotinib, it is slightly higher (3.19), indicating that both compounds have comparable synthetic feasibility. Notably, erlotinib has a Brenk alert due to the presence of a triple bond, which may raise concerns regarding potential reactivity and stability. The comparative ADME analysis of **7k** and erlotinib reveals that while erlotinib exhibits higher lipophilicity and BBB permeability, it also poses significant risks of CYP inhibition and has lower solubility. In contrast, **7k** demonstrates better solubility, a favourable hydrogen bonding network, reduced interactions with the CYP enzyme, and lower risks of CNS toxicity, making it a promising alternative for selective EGFR inhibition. These findings, supported by docking, molecular dynamics (MD), and experimental studies, position compound **7k** as a viable lead compound for further drug development.

## 3. Conclusion

This study involved the design, synthesis, and biological evaluation of a novel series of 5-ethylsulfonyl-indazole-3-carbohydrazide derivatives (**7a–o**) as dual-target EGFR/VEGFR-2 inhibitors with significant antiproliferative efficacy. Compounds **7j** and **7k** exhibited the most potent antiproliferative activities (GI_50_ = 27 and 25 nM, respectively), surpassing erlotinib through combined inhibition of EGFR and VEGFR-2. The examination of the structure–activity relationship highlighted the crucial contributions of the indazole moiety, sulphonyl moiety, and electron-donating substituents in enhancing activity. Furthermore, **7j** and **7k** exhibited significant apoptotic potential. The multi-target nature of these compounds highlights their potential as adaptable therapeutic agents for cancer treatment. The molecular docking, molecular dynamics (MD) simulations, and density functional theory (DFT) calculations provided valuable insights into the binding interactions of these compounds with EGFR and VEGFR-2. Specifically, the docking studies revealed that compound **7k** demonstrated the highest binding affinity to both EGFR and VEGFR-2, with strong hydrogen bonding and hydrophobic interactions stabilising its binding in the active sites. The key interactions involved hydrogen bonds with Met769 and Lys721 in EGFR, as well as Glu885 and Asp1046 in VEGFR-2, reinforcing the dual inhibition activity. MD simulations and DFT analysis showed stable ligand-receptor interactions, confirming the efficacy of the compounds as dual inhibitors. Further ADME studies highlighted the favourable pharmacokinetic profile of compound **7k**, with promising drug-likeness properties and reduced risks of adverse metabolic interactions. Further research is needed to determine their *in vivo* efficacy and toxicity. Future studies will focus on enhancing EGFR and VEGFR-2 inhibition while maintaining the compounds’ anticancer properties.

## 4. Experimental

### 4.1. General details: see Appendix A (Supplementary File)

All spectra (MS, ^1^H NMR, and ^13^C NMR) of compounds **2–6** and **7a–o** were included in the supplementary file (**Figures S1–S39**).

### 4.2. Chemistry

#### 4.2.1. Synthesis of 5-bromo-1H-indazole-3-carboxylic acid (2)

A suspension of indazole-3-carboxylic acid **1** (10.0 g, 61.7 mmol) in glacial acetic acid (300 ml) was heated to 90 °C until a clear solution was obtained. A solution of bromine (6.35 ml, 123.4 mmol) in glacial acetic acid (10 ml) was added slowly to the reaction mixture while maintaining the temperature at 90 °C. The reaction mixture was further heated at 90 °C for 16 h. After completion, the reaction mixture was cooled to room temperature and poured into ice water. The resulting mixture was stirred at room temperature for 15 min, leading to the formation of a solid precipitate. The solid was collected by filtration, washed with cold water, and dried under vacuum at room temperature to afford 5-bromo-1*H*-indazole-3-carboxylic acid **2** as a white solid (12.0 g, 81% yield) that was used for the next step without further purification.

^1^H NMR (400 MHz, DMSO) δ 14.01 (s, 1H), 8.22 (dd, *J* = 1.9, 0.7 Hz, 1H), 7.65 (dd, *J* = 8.8, 0.7 Hz, 1H), 7.56 (dd, *J* = 8.8, 1.9 Hz, 1H). MS (*m*/*z*): 238.8 (M-1).

#### 4.2.2. Synthesis of ethyl 5-bromo-1H-indazole-3-carboxylate (3)

To a suspension of 5-bromo-*1H*-indazole-3-carboxylic acid **2** (8.0 g, 33.19 mmol) in dry ethanol (200 ml), concentrated sulphuric acid (4 ml) was added. The reaction mixture was heated at 90 °C for 16 h under nitrogen atmosphere. After completion, the reaction mixture was cooled to room temperature, and ethanol was evaporated under vacuum. The resulting residue was dissolved in ethyl acetate (500 ml) and washed sequentially with saturated sodium bicarbonate solution (150 ml) and water (150 ml). The organic layer was dried over anhydrous Na_2_SO_4_, filtered, and concentrated under reduced pressure to afford ethyl 5-bromo-1*H*-indazole-3-carboxylate **3** as a white solid (8.57 g, 96% yield) that was used for the next step without further purification.

^1^H NMR (400 MHz, DMSO) δ 8.19 (dd, *J* = 1.9, 0.7 Hz, 1H), 7.66 (dd, *J* = 8.9, 0.7 Hz, 1H), 7.58 (dd, *J* = 8.9, 1.9 Hz, 1H), 4.40 (q, *J* = 7.1 Hz, 2H), 1.37 (t, *J* = 7.1 Hz, 3H). MS (*m*/*z*): 266.8 (M-1)

#### 4.2.3. Synthesis of ethyl 5-(ethylthio)-1H-indazole-3-carboxylate (4)

A two-necked flask equipped with a condenser was dried under vacuum using a heat gun, then allowed to cool to room temperature under nitrogen atmosphere. To the flask, 1,4-dioxane (120 ml) was added and degassed. Xantphos (1.29 g, 10 mol%) and Pd_2_(dba)_3_ (1.02 g, 5 mol%) were introduced into the flask, and the mixture was further degassed. Subsequently, ethyl 5-bromo-1*H*-indazole-3-carboxylate **3** (6.0 g, 22.3 mmol) was added, followed by a solution of ethanethiol (1.65 ml, 22.3 mmol) and DIPEA (11.65 ml, 66.9 mmol). The reaction mixture was heated at 90 °C for 4 h under a nitrogen atmosphere. Upon completion, the reaction mixture was diluted with ethyl acetate and filtered through a celite pad. The filtrate was concentrated, and the crude product was purified by flash chromatography using a gradient elution of 5–50% EtOAc in hexane, yielding the desired product **4** as a yellow oil (3.07 g, 55% yield). MS (*m*/*z*): 249.0 (M-1).

^1^H NMR (400 MHz, DMSO) δ 13.95 (s, 1H), 7.99 (dd, *J* = 1.7, 0.8 Hz, 1H), 7.63 (dd, *J* = 8.8, 0.8 Hz, 1H), 7.42 (dd, *J* = 8.8, 1.7 Hz, 1H), 4.39 (q, *J* = 7.1 Hz, 2H), 2.99 (q, *J* = 7.3 Hz, 2H), 1.37 (t, *J* = 7.1 Hz, 3H), 1.23 (t, *J* = 7.3 Hz, 3H).

#### 4.2.4. Synthesis of ethyl 5-(ethylsulfonyl)-1H-indazole-3-carboxylate (5)

A solution of ethyl 5-(ethylthio)-1*H*-indazole-3-carboxylate **4** (3.0 g, 11.98 mmol) in dichloromethane (75 ml) was cooled to 0 °C. *m*-Chloroperbenzoic acid (6.2 g, 35.95 mmol) was added portionwise while maintaining the temperature at 0 °C. The reaction mixture was then allowed to warm to room temperature and stirred for 2 h. Upon completion, the reaction mixture was diluted with DCM and sequentially washed with saturated NaHCO_3_ solution, followed by brine. The organic layer was then dried over anhydrous Na_2_SO_4_, filtered, and concentrated under reduced pressure. The crude product was purified by flash chromatography using a gradient elution of 5–50% EtOAc in DCM, affording the desired product **5** as an off-white solid (2.1 g, 60% yield). ^1^H NMR (400 MHz, DMSO) δ 8.60 (t, *J* = 1.2 Hz, 1H), 8.02–7.72 (m, 2H), 4.43 (q, *J* = 7.1 Hz, 2H), 1.38 (t, *J* = 7.1 Hz, 3H), 1.11 (t, *J* = 7.3 Hz, 3H). MS (*m*/*z*): 280.9 (M-1).

#### 4.2.5. Synthesis of 5-(ethylsulfonyl)-1H-indazole-3-carbohydrazide (6)

A solution of ethyl 5-(ethylsulfonyl)-1*H*-indazole-3-carboxylate **5** (2.0 g, 7.08 mmol) and hydrazine hydrate (2.61 ml, 42.48 mmol) in ethanol (25 ml) was heated at 90 °C for 6 h. After completion, the reaction mixture was cooled to room temperature, and the precipitated solids were filtered, washed with cold ethanol, and dried under vacuum at room temperature to afford the desired product **6** as a light pink solid (1.61 g, 85% yield) that was used for the next step without further purification.

^1^H NMR (400 MHz, DMSO) δ 13.34 (s, 1H), 8.47 (dd, *J* = 1.7, 0.8 Hz, 1H), 7.87–7.54 (m, 2H), 5.47 (s, 1H), 4.84 (s, 2H), 3.28 (q, *J* = 7.3 Hz, 2H), 1.10 (t, *J* = 7.3 Hz, 3H). MS (*m*/*z*): 266.50 (M-1).

#### 4.2.6. General procedure for synthesis of compounds (7a–o)

To a stirred solution of 5-(ethylsulfonyl)-1*H*-indazole-3-carbohydrazide **(6)** (0.37 mmol, 1eq) in 30 ml of absolute ethanol containing a few drops of glacial acetic acid as a catalyst, suitable aldehyde (0.5 mmol, 1.3 eq) was progressively added, and the resultant mixture was refluxed overnight. After the reaction was complete (as monitored by TLC), it was allowed to cool, and the formed precipitate was filtered and washed several times with ethanol to remove any excess unreacted aldehyde.

##### 4.2.6.1. (*E*)-*N′*-(4-Cyanobenzylidene)-5-(ethylsulfonyl)-1*H-*indazole-3-carbohydrazide (7a)

Yield: 0.13 g (91%), white solid, mp >300 °C. ^1^H NMR (500 MHz, δ ppm DMSO-*d*_6_): 14.35 (s, 1H, –NH–N), 12.43 (s, 1H, NH–C = O), 8.71 (s, 1H, Ar–H), 8.60 (s, 1H, Ar–H), 7.93–7.81 (m, 6H, Ar–H, CH=N), 3.31 (q, *J* = 7.3 Hz, 2H, CH_2_–CH_3_), 1.08 (t, *J* = 7.4 Hz, 3H, CH_2_–CH_3_). ^13^C NMR (126 MHz, DMSO-*d*_6_): 158.8, 146.8, 143.0, 139.4, 139.2, 133.3, 128.2, 125.8, 124.0, 121.8, 119.2, 113.0, 112.4, 50.1, 7.9. Anal. Calc. (%) for C_18_H_15_N_5_O_3_S: C, 56.68; H, 3.96; N, 18.36; S, 8.41. Found: C, 56.76; H, 4.09; N, 18.45; S, 8.35.

##### 4.2.6.2. (*E*)-5-(ethylsulfonyl)-*N′*-(pyridin-3-ylmethylene)-1*H*-indazole-3-carbohydrazide (7b)

Yield: 0.12 g (83%), white solid, mp >300 °C. ^1^H NMR (500 MHz, δ ppm DMSO-*d*_6_): 14.29 (s, 1H, –NH–N), 12.20 (s, 1H, NH–C = O), 8.70 (s, 1H, Ar–H), 8.54 (s, 1H, Ar–H), 8.34 (d, *J* = 2.3 Hz, 1H, Ar–H), 8.11–8.05 (m, 1H, Ar–H), 7.92–7.83 (m, 3H, Ar–H, CH=N), 6.89 (d, *J* = 8.7 Hz, 1H, Ar–H), 3.30 (q, *J* = 7.2 Hz, 2H, CH_2_–CH_3_), 1.07 (t, *J* = 7.3 Hz, 3H, CH_2_–CH_3_). ^13^C NMR (126 MHz, DMSO-*d*_6_): 165.1, 158.5, 147.9, 146.0, 143.0, 139.4, 136.6, 133.1, 125.8, 124.8, 124.0, 121.8, 112.9, 112.0, 50.1, 7.9. Anal. Calc. (%) for C_16_H_15_N_5_O_3_S: C, 53.77; H, 4.23; N, 19.60; S, 8.97. Found: C, 53.71; H, 4.32; N, 19.49; S, 9.05.

##### 4.2.6.3. (*E*)-5-(ethylsulfonyl)-*N′*-((1-methyl-1*H*-indazol-5-yl)methylene)-1*H*-indazole-3-carbohydrazide (7c)

Yield: 0.14 g (92%), white solid, mp >300 °C. ^1^H NMR (500 MHz, δ ppm DMSO-*d*_6_): 14.29 (s, 1H, –NH–N), 12.13 (s, 1H, NH–C = O), 8.73 (s, 1H, Ar–H), 8.66 (s, 1H, Ar–H), 8.10 (s, 1H, Ar–H), 7.95 (s, 1H, Ar–H), 7.90–7.85 (m, 3H, Ar–H, CH=N), 7.69 (d, *J* = 8.9 Hz, 1H, Ar–H), 4.03 (s, 3H, –N–N–CH_3_), 3.31 (q, *J* = 7.3 Hz, 2H, CH_2_–CH_3_), 1.08 (t, *J* = 7.2 Hz, 3H, CH_2_–CH_3_). ^13^C NMR (126 MHz, DMSO-*d*_6_): 158.4, 149.5, 143.0, 140.8, 139.5, 133.9, 133.0, 127.7, 125.8, 124.2, 124.0, 122.6, 121.8, 113.0, 111.0, 50.1, 36.1, 7.9. Anal. Calc. (%) for C_19_H_18_N_6_O_3_S: C, 55.60; H, 4.42; N, 20.48; S, 7.81. Found: C, 55.67; H, 4.35; N, 20.55; S, 7.78.

##### 4.2.6.4. (*E*)-5-(ethylsulfonyl)-*N′*-(quinolin-6-ylmethylene)-1*H*-indazole-3-carbohydrazide (7d)

Yield: 0.14 g (93%), white solid, mp >300 °C. ^1^H NMR (500 MHz, δ ppm DMSO-*d*_6_): 14.33 (s, 1H, **–**NH–N), 12.36 (s, 1H, NH–C = O), 8.88 (d, *J* = 4.3 Hz, 1H, Ar–H), 8.74 (d, *J* = 8.3 Hz, 2H, Ar–H), 8.43 (d, *J* = 8.0 Hz, 1H, Ar–H), 8.22–8.14 (m, 2H, Ar–H), 8.04 (d, *J* = 8.8 Hz, 1H, Ar–H), 7.94–7.83 (m, 2H, Ar–H, CH=N), 7.54 (dd, *J* = 8.4, 4.3 Hz, 1H, Ar–H), 3.31 (q, *J* = 7.3 Hz, 2H, CH_2_–CH_3_), 1.09 (t, *J* = 7.4 Hz, 3H, CH_2_–CH_3_). ^13^C NMR (126 MHz, DMSO-*d*_6_): 158.7, 151.8, 149.0, 148.0, 143.1, 139.4, 137.1, 136.9, 133.2, 130.2, 129.3, 129.1, 128.5, 125.8, 124.0, 122.7, 121.8, 113.0, 50.1, 7.9. Anal. Calc. (%) for C_20_H_17_N_5_O_3_S: C, 58.96; H, 4.21; N, 17.19; S, 7.87. Found: C, 58.90; H, 4.30; N, 17.26; S, 7.93.

##### 4.2.6.5. (*E*)-5-(ethylsulfonyl)-*N′*-(thiazol-2-ylmethylene)-1*H*-indazole-3-carbohydrazide (7e)

Yield: 0.11 g (81%), white solid, mp >300 °C.^1^H NMR (500 MHz, δ ppm DMSO-*d*_6_): 14.44 (s, 1H, –NH–N), 12.61 (s, 1H, NH–C = O), 8.77 (s, 1H, Ar–H), 8.70 (s, 1H, Ar–H), 7.94–7.85 (m, 3H, Ar–H, CH=N), 7.81 (d, *J* = 3.2 Hz, 1H, Ar–H), 3.31 (q, *J* = 7.3 Hz, 2H, CH_2_–CH_3_), 1.08 (t, *J* = 7.4 Hz, 3H, CH_2_–CH_3_). ^13^C NMR (126 MHz, DMSO-*d*_6_): 165.0, 158.6, 144.6, 143.1, 142.98, 139.0, 133.3, 125.9, 123.9, 122.6, 121.8, 113.0, 50.1, 7.8. Anal. Calc. (%) for C_14_H_13_N_5_O_3_S_2_: C, 46.27; H, 3.61; N, 19.27; S, 17.64. Found: C, 46.21; H, 3.69; N, 19.35; S, 17.60.

##### 4.2.6.6. (*E*)-5-(ethylsulfonyl)-*N′*-((1-methyl-1*H*-indol-5-yl)methylene)-1*H*-indazole-3-carbohydrazide (7f)

Yield: 0.11 g (73%), red solid, mp >300 °C. ^1^H NMR (500 MHz, δ ppm DMSO-*d*_6_): 14.27 (s, 1H, –NH–N), 12.00 (s, 1H, NH–C = O), 8.72 (t, *J* = 1.3 Hz, 1H, Ar–H), 8.61 (s, 1H, Ar–H), 7.87 (dd, *J* = 3.6, 1.3 Hz, 2H, Ar–H, CH=N), 7.79 (s, 1H, Ar–H), 7.63 (dd, *J* = 8.6, 1.6 Hz, 1H, Ar–H), 7.50 (d, *J* = 8.6 Hz, 1H, Ar–H), 7.35 (d, *J* = 3.1 Hz, 1H, Ar–H), 6.49 (dd, *J* = 3.1, 0.8 Hz, 1H, Ar–H), 3.78 (s, 3H, –N–CH_3_), 3.30 (q, *J* = 7.4 Hz, 2H, CH_2_–CH_3_), 1.08 (t, *J* = 7.3 Hz, 3H, CH_2_–CH_3_). ^13^C NMR (126 MHz, DMSO-*d*_6_): 158.3, 150.7, 143.0, 139.7, 137.9, 133.0, 131.3, 128.5, 126.1, 125.7, 124.1, 121.9, 121.75, 119.9, 112.9, 110.9, 101.8, 50.1, 33.2, 7.9. Anal. Calc. (%) for C_20_H_19_N_5_O_3_S: C, 58.67; H, 4.68; N, 17.10; S, 7.83. Found: C, 58.74; H, 4.61; N, 17.16; S, 7.89.

##### 4.2.6.7. (*E*)-5-(ethylsulfonyl)-*N′*-(3-hydroxy-4-methoxybenzyli­dene)-1*H*-indazole-3-carbohydrazide (7 g)

Yield: 0.13 g (88%), white solid, mp >300 °C. ^1^H NMR (500 MHz, δ ppm DMSO-*d*_6_): 14.26 (s, 1H, –NH–N), 11.99 (s, 1H, NH–C = O), 9.35 (s, 1H, Ar–OH), 8.71 (s, 1H, Ar–H), 8.40 (s, 1H, Ar–H), 7.92–7.82 (m, 2H, Ar–H, CH=N), 7.25 (d, *J* = 2.3 Hz, 1H, Ar–H), 7.00 (dd, *J* = 8.1, 2.2 Hz, 1H, Ar–H), 6.94 (d, *J* = 8.1 Hz, 1H, Ar–H), 3.76 (s, 3H, Ar–OCH_3_), 3.30 (q, *J* = 7.3 Hz, 2H, CH_2_–CH_3_), 1.07 (t, *J* = 7.2 Hz, 3H, CH_2_–CH_3_). ^13^C NMR (126 MHz, DMSO-*d*_6_): 158.3, 150.3, 149.0, 147.4, 143.0, 139.6, 133.0, 127.7, 125.8, 124.1, 121.7, 121.2, 112.9, 112.7, 112.3, 56.1, 50.1, 7.9. Anal. Calc. (%) for C_18_H_18_N_4_O_5_S: C, 53.72; H, 4.51; N, 13.92; S, 7.97. Found: C, 53.61; H, 4.42; N, 14.01; S, 7.93.

##### 4.2.6.8. (*E*)-5-(ethylsulfonyl)-*N′*-(pyrimidin-5-ylmethylene)-1*H*-indazole-3-carbohydrazide (7h)

Yield: 0.11 g (84%), white solid, mp >300 °C. ^1^H NMR (500 MHz, δ ppm DMSO-*d*_6_): 14.36 (s, 1H, –NH–N), 12.55 (s, 1H, NH–C = O), 9.18 (s, 1H, Ar–H), 9.06 (s, 2H, Ar–H), 8.70 (s, 1H, Ar–H), 8.60 (s, 1H, Ar–H), 7.94–7.82 (m, 2H, Ar–H, CH=N), 3.30 (q, *J* = 7.3 Hz, 2H, CH_2_–CH_3_), 1.08 (t, *J* = 7.2 Hz, 3H, CH_2_–CH_3_). Anal. Calc. (%) for C_15_H_14_N_6_O_3_S: C, 50.27; H, 3.94; N, 23.45; S, 8.95. Found: C, 50.39; H, 4.02; N, 23.54; S, 9.01.

##### 4.2.6.9. (*E*)-5-(ethylsulfonyl)-*N′*-(4-hydroxybenzylidene)-1*H*-indazole-3-carbohydrazide (7i)

Yield: 0.12 g (83%), white solid, mp >300 °C. ^1^H NMR (500 MHz, δ ppm DMSO-*d*_6_): 11.97 (s, 1H, NH–C = O), 8.71 (s, 1H, Ar–H), 8.44 (s, 1H, Ar–H), 7.91–7.81 (m, 2H, Ar–H, CH=N), 7.52 (d, *J* = 8.6 Hz, 2H, Ar–H), 6.81 (d, *J* = 8.5 Hz, 2H, Ar–H), 3.30 (q, *J* = 7.3 Hz, 2H, CH_2_–CH_3_), 1.07 (t, *J* = 7.2 Hz, 3H, CH_2_–CH_3_). ^13^C NMR (126 MHz, DMSO-*d*_6_): 160.0, 158.3, 149.1, 143.0, 139.59, 133.0, 129.4, 125.9, 125.7, 124.1, 121.7, 116.3, 112.9, 50.1, 7.9. Anal. Calc. (%) for C_17_H_16_N_4_O_4_S: C, 54.83; H, 4.33; N, 15.05; S, 8.61. Found: C, 54.90; H, 4.29; N, 15.17; S, 8.55.

##### 4.2.6.10. (*E*)-*N′*-(benzo[d]thiazol-2-ylmethylene)-5-(ethylsul­fonyl)-1*H*-indazole-3-carbohydrazide (7j)

Yield: 0.13 g (84%), white solid, mp >300 °C. ^1^H NMR (500 MHz, δ ppm DMSO-*d*_6_): 14.44 (s, 1H, –NH–N), 12.85 (s, 1H, NH–C = O), 8.87 (s, 1H, Ar–H), 8.72 (s, 1H, Ar–H), 8.12 (d, *J* = 7.9 Hz, 1H, Ar–H), 8.01 (d, *J* = 8.0 Hz, 1H, Ar–H), 7.96–7.86 (m, 2H, Ar–H, CH=N), 7.54–7.45 (m, 2H, Ar–H), 3.32 (q, *J* = 7.4 Hz, 2H, CH_2_–CH_3_), 1.08 (t, *J* = 7.4 Hz, 3H, CH_2_–CH_3_). ^13^C NMR (126 MHz, DMSO-*d*_6_): 165.8, 158.7, 153.7, 143.1, 143.0, 138.84, 134.6, 133.4, 127.2, 125.9, 123.8, 123.1, 121.8, 113.1, 50.1, 7.9. Anal. Calc. (%) for C_18_H_15_N_5_O_3_S_2_: C, 52.29; H, 3.66; N, 16.94; S, 15.51. Found: C, 52.22; H, 3.70; N, 15.02; S, 15.43.

##### 4.2.6.11. (*E*)-5-(ethylsulfonyl)-*N′*-((6-methoxypyridin-3-yl)methylene)-1*H*-indazole-3-carbohydrazide (7k)

Yield: 0.13 g (88%), white solid, mp >300 °C. ^1^H NMR (500 MHz, δ ppm DMSO-*d*_6_): 14.29 (s, 1H, –NH–N), 12.20 (s, 1H, NH–C = O), 8.70 (s, 1H, Ar–H), 8.54 (s, 1H, Ar–H), 8.34 (d, *J* = 2.3 Hz, 1H, Ar–H), 8.08 (dd, *J* = 9.0, 2.3 Hz, 1H, Ar–H), 7.92–7.83 (m, 2H, Ar–H, CH=N), 6.89 (d, *J* = 8.7 Hz, 1H, Ar–H), 3.86 (s, 3H, Ar–OCH_3_), 3.30 (q, *J* = 7.2 Hz, 2H, CH_2_–CH_3_), 1.07 (t, *J* = 7.3 Hz, 3H, CH_2_–CH_3_). ^13^C NMR (126 MHz, DMSO-*d*_6_): 165.1, 158.5, 147.9, 146.0, 143.0, 139.4, 136.6, 133.1, 125.8, 124.8, 124.0, 121.8, 112.9, 112.0, 54.1, 50.1, 7.9. Anal. Calc. (%) for C_17_H_17_N_5_O_4_S: C, 52.71; H, 4.42; N, 18.08; S, 8.28. Found: C, 52.59; H, 4.33; N, 18.11; S, 8.35.

##### 4.2.6.12. (*E*)-5-(ethylsulfonyl)-*N′*-(3-methoxybenzylidene)-1*H*-indazole-3-carbohydrazide (7 l)

Yield: 0.12 g (85%), white solid, mp >300 °C. ^1^H NMR (500 MHz, δ ppm DMSO-*d*_6_): 14.30 (s, 1H, –NH–N), 12.19 (s, 1H, NH–C = O), 8.71 (s, 1H, Ar–H), 8.53 (s, 1H, Ar–H), 7.93–7.84 (m, 2H, Ar–H, CH=N), 7.34 (t, *J* = 8.0 Hz, 1H, Ar–H), 7.26–7.21 (m, 2H, Ar–H), 6.98 (dd, *J* = 8.1, 2.6 Hz, 1H, Ar–H), 3.77 (s, 3H, Ar–OCH_3_), 3.30 (q, *J* = 7.7 Hz, 2H, CH_2_–CH_3_), 1.08 (t, *J* = 7.2 Hz, 3H, CH_2_–CH_3_).^13^C NMR (126 MHz, DMSO-*d*_6_): 160.1, 158.6, 148.7, 148.6, 143.0, 139.4, 136.4, 133.1, 130.5, 125.8, 124.2, 121.8, 120.6, 117.0, 116.6, 112.9, 112.0, 111.3, 55.7, 50.1, 7.9. Anal. Calc. (%) for C_18_H_18_N_4_O_4_S: C, 55.95; H, 4.70; N, 14.50; S, 8.30. Found: C, 55.88; H, 4.62; N, 14.43; S, 8.37.

##### 4.2.6.13. (*E*)-*N′*-(cyclopropylmethylene)-5-(ethylsulfonyl)-1*H*-indazole-3-carbohydrazide (7 m)

Yield: 0.1 g (85%), white solid, mp >300 °C. ^1^H NMR (500 MHz, δ ppm DMSO-*d*_6_): 14.19 (s, 1H, –NH–N), 11.66 (s, 1H, NH–C = O), 8.67 (s, 1H, Ar–H), 7.86 (s, 2H, Ar–H, CH=N), 7.34 (d, *J* = 7.6 Hz, 1H, Ar–H), 3.29 (q, *J* = 7.4 Hz, 2H, CH_2_–CH_3_), 1.65 (tq, *J* = 8.3, 4.2 Hz, 1H, CH_2_–CH–CH_2_), 1.06 (t, *J* = 7.3 Hz, 3H, CH_2_–CH_3_), 0.86 (dd, *J* = 8.1, 2.7 Hz, 2H, CH_2_–CH–CH_2_), 0.62 (d, *J* = 6.2 Hz, 2H, CH_2_–CH–CH_2_). ^13^C NMR (126 MHz, DMSO-*d*_6_): 157.9, 143.0, 139.5, 132.9, 125.7, 124.1, 124.0, 121.7, 112.8, 50.1, 14.3, 7.8, 6.6. Anal. Calc. (%) for C_14_H_16_N_4_O_3_S: C, 52.49; H, 5.03; N, 17.49; S, 10.01. Found: C, 52.58; H, 5.09; N, 17.42; S, 9.96.

##### 4.2.6.14. (*E*)-*N′*-(cyclopentylmethylene)-5-(ethylsulfonyl)-1*H*-indazole-3-carbohydrazide (7n)

Yield: 0.1 g (80%), white solid, mp >300 °C. ^1^H NMR (500 MHz, δ ppm DMSO-*d*_6_): 14.20 (s, 1H, –NH–N), 11.67 (s, 1H, NH–C = O), 8.66 (s, 1H, Ar–H), 7.87–7.82 (m, 2H, Ar–H, CH=N), 7.77 (d, *J* = 6.1 Hz, 1H, Ar–H), 3.29 (q, *J* = 7.4 Hz, 2H, CH_2_–CH_3_), 2.66 (h, *J* = 7.7 Hz, 1H, CH_2_–CH–CH_2_), 1.82–1.73 (m, 2H, CH_2_–CH_2_–CH_2_–CH_2_), 1.65–1.42 (m, 6H, CH_2_–CH_2_–CH_2_–CH_2_), 1.06 (t, *J* = 7.3 Hz, 3H, CH_2_–CH_3_). ^13^C NMR (126 MHz, DMSO-*d*_6_): 158.2, 156.6, 143.0, 139.5, 132.9, 125.7, 124.0, 121.7, 112.8, 50.1, 42.7, 30.6, 25.6, 7.8. Anal. Calc. (%) for C_16_H_20_N_4_O_3_S: C, 55.16; H, 5.79; N, 16.08; S, 9.20. Found: C, 55.12; H, 5.83; N,16.17; S, 9.23.

##### 4.2.6.15. (*E*)-5-(ethylsulfonyl)-*N′*-((1-methyl-1*H*-pyrazol-3-yl)methylene)-1*H*-indazole-3-carbohydrazide (7o)

Yield: 0.11 g (82%), white solid, mp >300 °C. ^1^H NMR (500 MHz, δ ppm DMSO-*d*_6_): 14.29 (s, 1H, –NH–N), 12.15 (s, 1H, NH–C = O), 8.70 (s, 1H, Ar–H), 8.53 (s, 1H, Ar–H), 7.90–7.87 (m, 2H, Ar–H, CH=N), 7.73 (d, *J* = 2.3 Hz, 1H, Ar–H), 6.58 (d, *J* = 2.4 Hz, 1H, Ar–H), 3.84 (s, 3H, –N–CH_3_), 3.30 (q, *J* = 7.2 Hz, 2H, CH_2_–CH_3_), 1.07 (t, *J* = 7.2 Hz, 3H, CH_2_–CH_3_). ^13^C NMR (126 MHz, DMSO-*d*_6_): 158.4, 148.0, 143.5, 143.0, 139.4, 133.1, 125.8, 124.0, 121.7, 112.9, 103.6, 50.1, 39.3, 7.8. Anal. Calc. (%) for C_15_H_16_N_6_O_3_S: C, 49.99; H, 4.48; N, 23.32; S, 8.90. Found: C, 50.05; H, 4.39; N,23.41; S, 8.88.

### 4.3. Biology

#### 4.3.1. Cell viability assay

The viability of **7a–o** was determined using the normal human mammary gland epithelial (MCF-10A) cell line^46,^^47^. After four days of incubation on MCF-10A cells with 50 µM of each tested compound; see Appendix A for more information.

#### 4.3.2. Antiproliferative assay

The MTT assay was employed to assess the antiproliferative efficacy of **7a–o** against four human cancer cell lines, utilising Erlotinib as a control^48^^,^^49^. Refer to Appendix A for additional information.

#### 4.3.3. EGFR inhibitory assay

The EGFR-TK assay evaluated the inhibitory efficacy of the most potent antiproliferative derivatives **7 g**, **7i–7l**, and **7o**, against EGFR^50^. For additional information, refer to Appendix A.

#### 4.3.4. VEGFR-2 inhibitory assay

Compounds **7 g**, **7i–7l**, and **7o** were evaluated for their capacity to inhibit VEGFR-2, with sorafenib serving as the control agent^52^. The results are expressed as IC_50_ values. Appendix A delineates further experimental specifics.

#### 4.3.5. Apoptotic markers assay

Compounds **7j**, **7k**, and **7o** were tested as Bax and p53 activators and as down-regulators of the anti-apoptotic protein Bcl-2 against the A-549 lung cancer cell line^60^. Appendix A gives more details.

### 4.4. Computational studies

Molecular docking simulations for EGFR (PDB ID: 1M17) and VEGFR-2 (PDB ID: 3WZE) were validated through a redocking test, in which the structures of the test proteins were maintained in a fixed state while the co-crystallized ligands (Erlotinib for EGFR and sorafenib for VEGFR-2) were redocked into their respective crystal-binding pockets. Refer to Appendix A for additional information.

## Supplementary Material

Supplementary_File_ Clean.docx

## Data Availability

The authors declare that the data supporting the findings of this study are available within the supplementary materials.
