# Supplementary material for: Design, synthesis, and antiproliferative activity of new 5-ethylsulfonyl-indazole-3-carbohydrazides as dual EGFR/VEGFR-2 kinases inhibitors
Source: J Enzyme Inhib Med Chem. 2025 Jun 24;40(1):2516075. doi: 10.1080/14756366.2025.2516075 (PMC12897534; doi:10.1080/14756366.2025.2516075)
Supplement: Supplementary_File_ Clean.docx [file IENZ_A_2516075_SM6197.docx]

**Design, synthesis, and antiproliferative activity of new 5-ethylsulfonyl-indaozle-3-carbohydrazides as dual EGFR/VEGFR-2 kinases inhibitors**

Supplementary Data

**
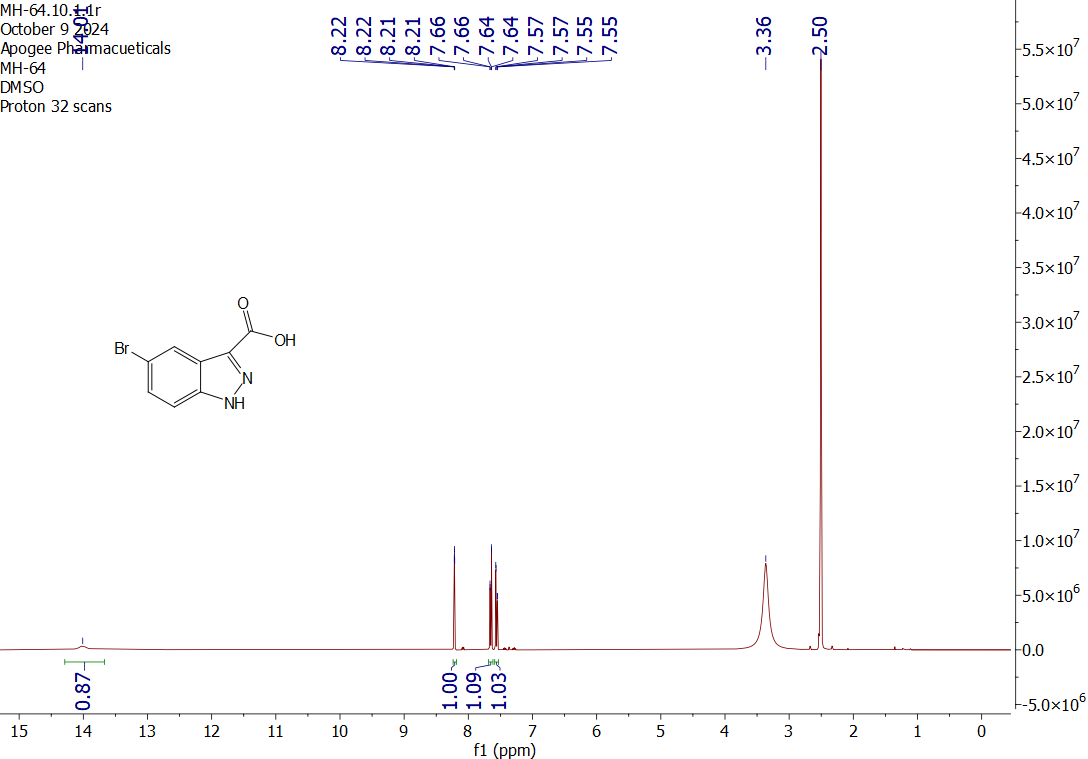
Figure S1**: ^1^H NMR spectrum (400 MHz, DMSO-*d*_6_) of compound **2**

**
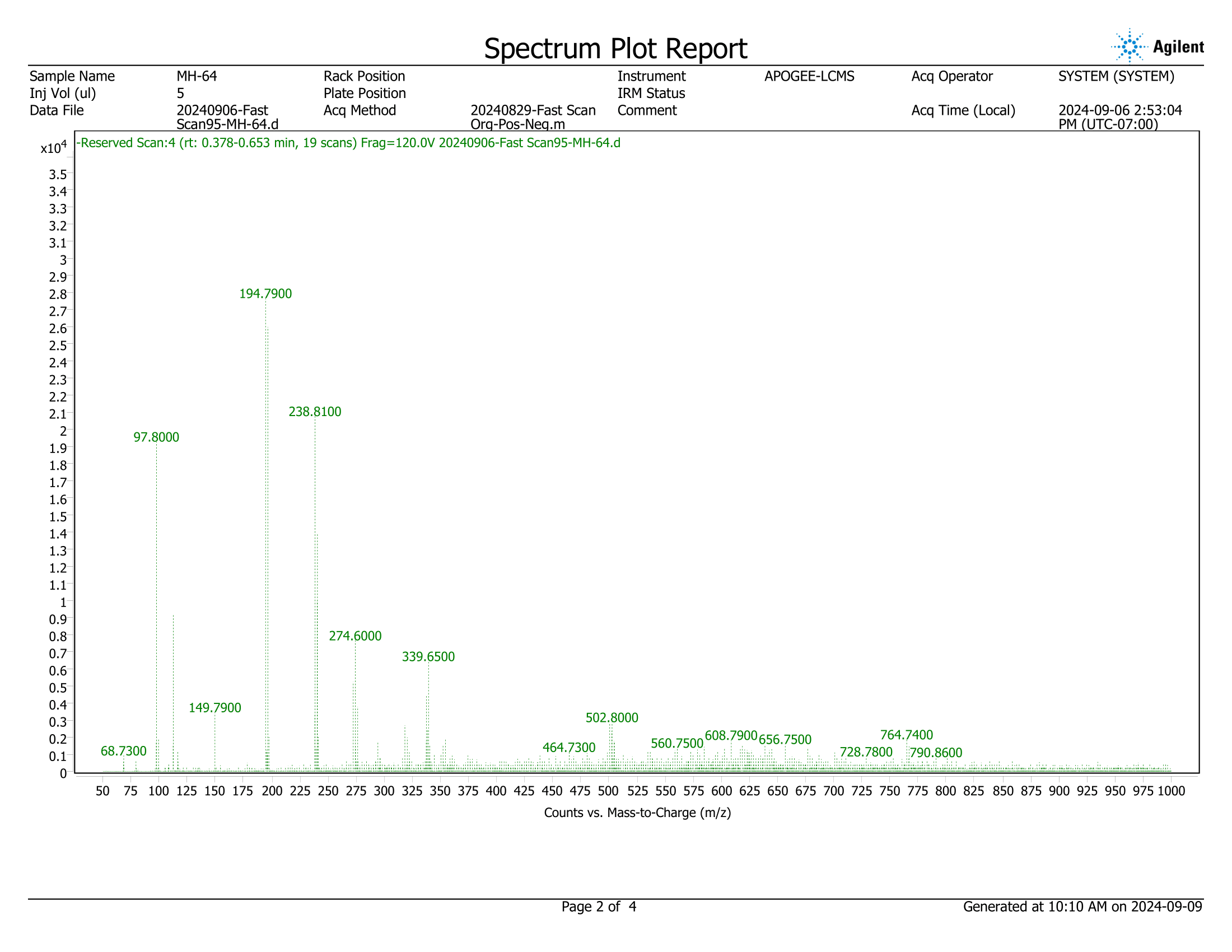
Figure S2**: HRMS spectrum of compound **2** showing [M-H] peak

**Figure S3**: ^1^H NMR spectrum (400 MHz, DMSO-*d*_6_) of compound **3**

**
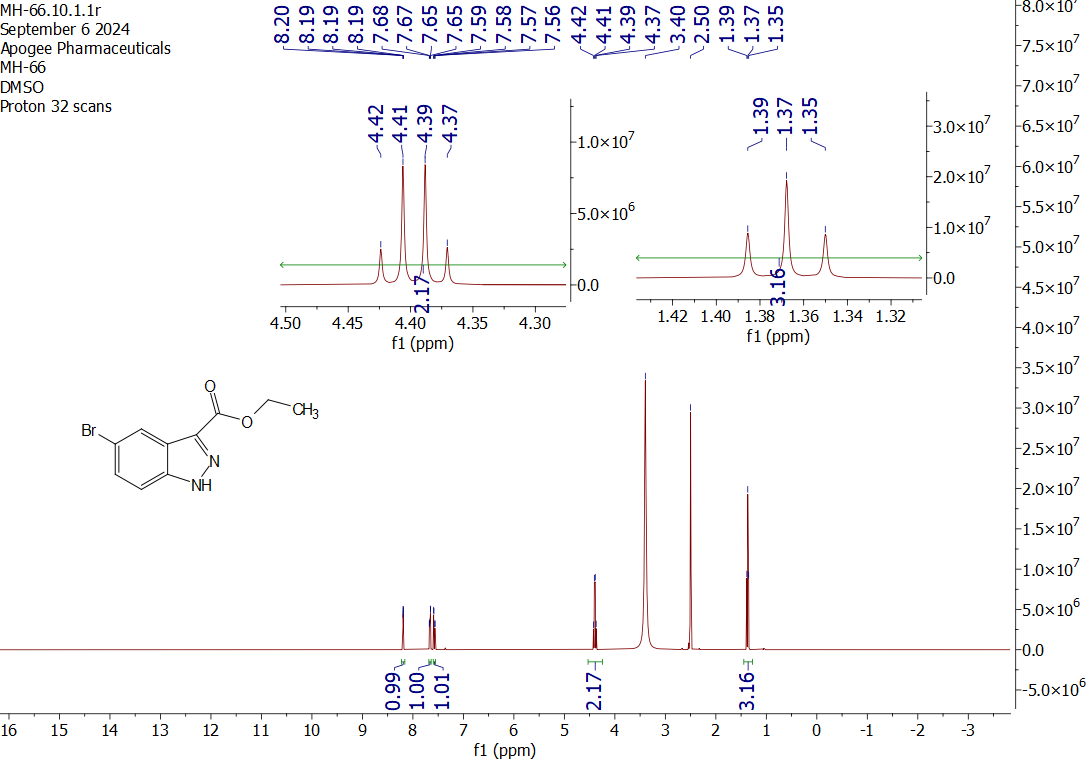
**

**Figure S4**: HRMS spectrum of compound **3** showing [M-H] peak

**
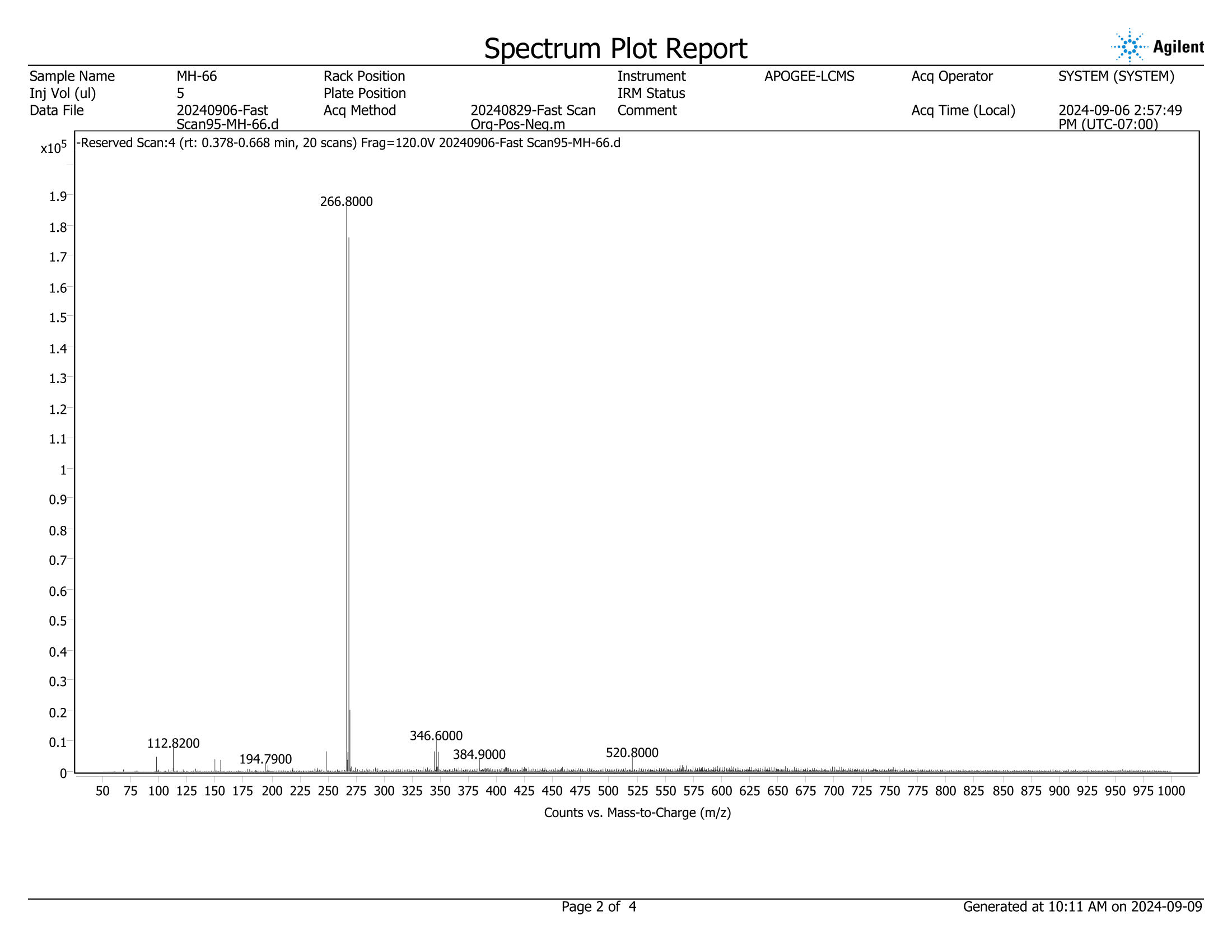
**

**Figure S5**: ^1^H NMR spectrum (400 MHz, DMSO-*d*_6_) of compound **4**

**
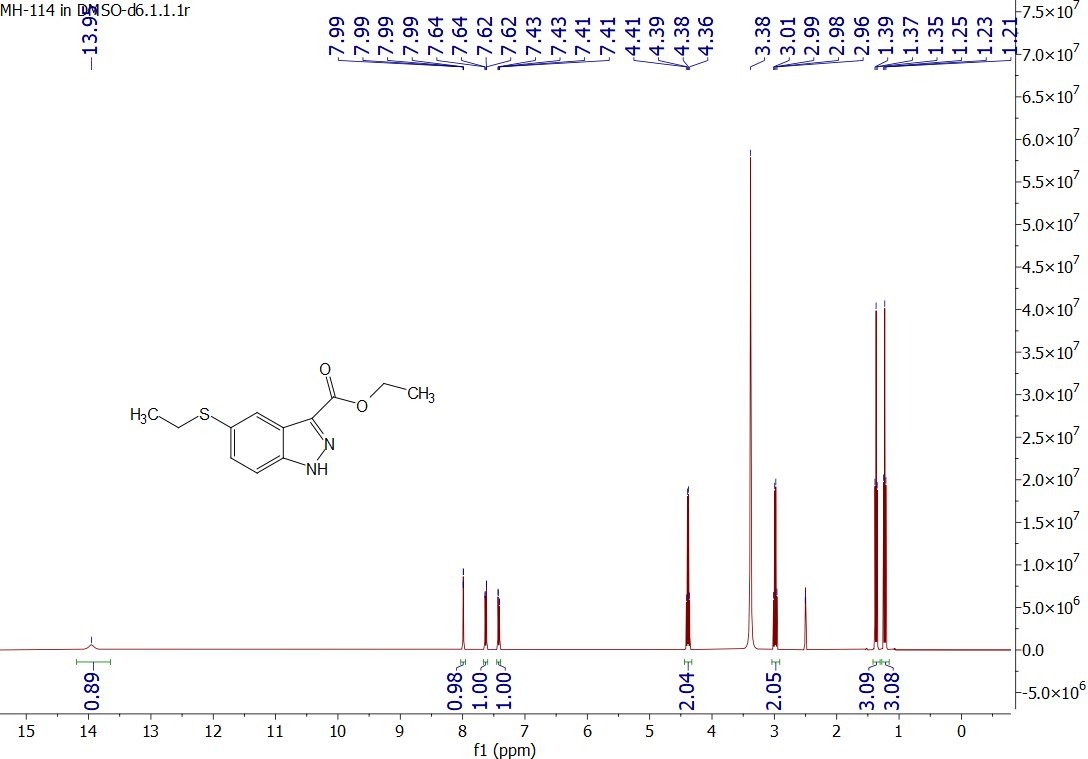
**

**
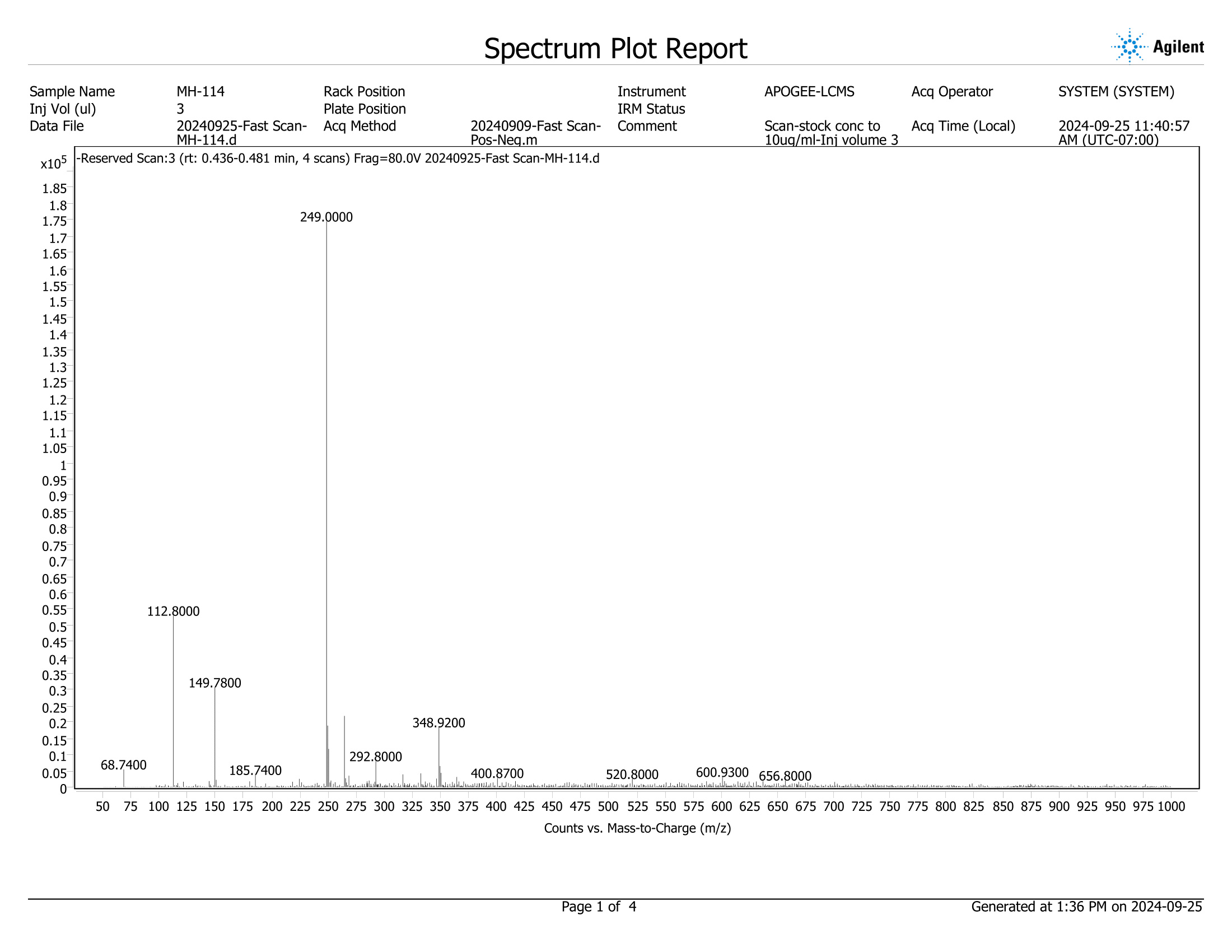
Figure S6**: HRMS spectrum of compound **4** showing [M-H] peak

**
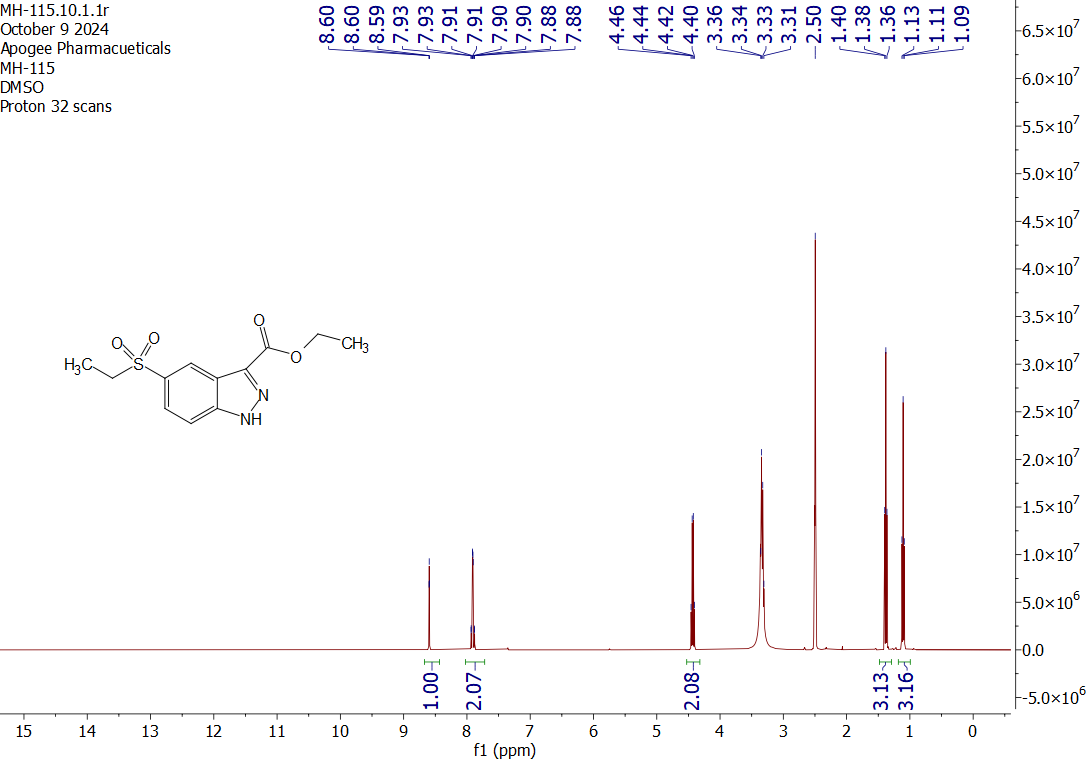
Figure S7**: ^1^H NMR spectrum (400 MHz, DMSO-*d*_6_) of compound **5**

**Figure S8**: HRMS spectrum of compound **5** showing [M-H] peak

**
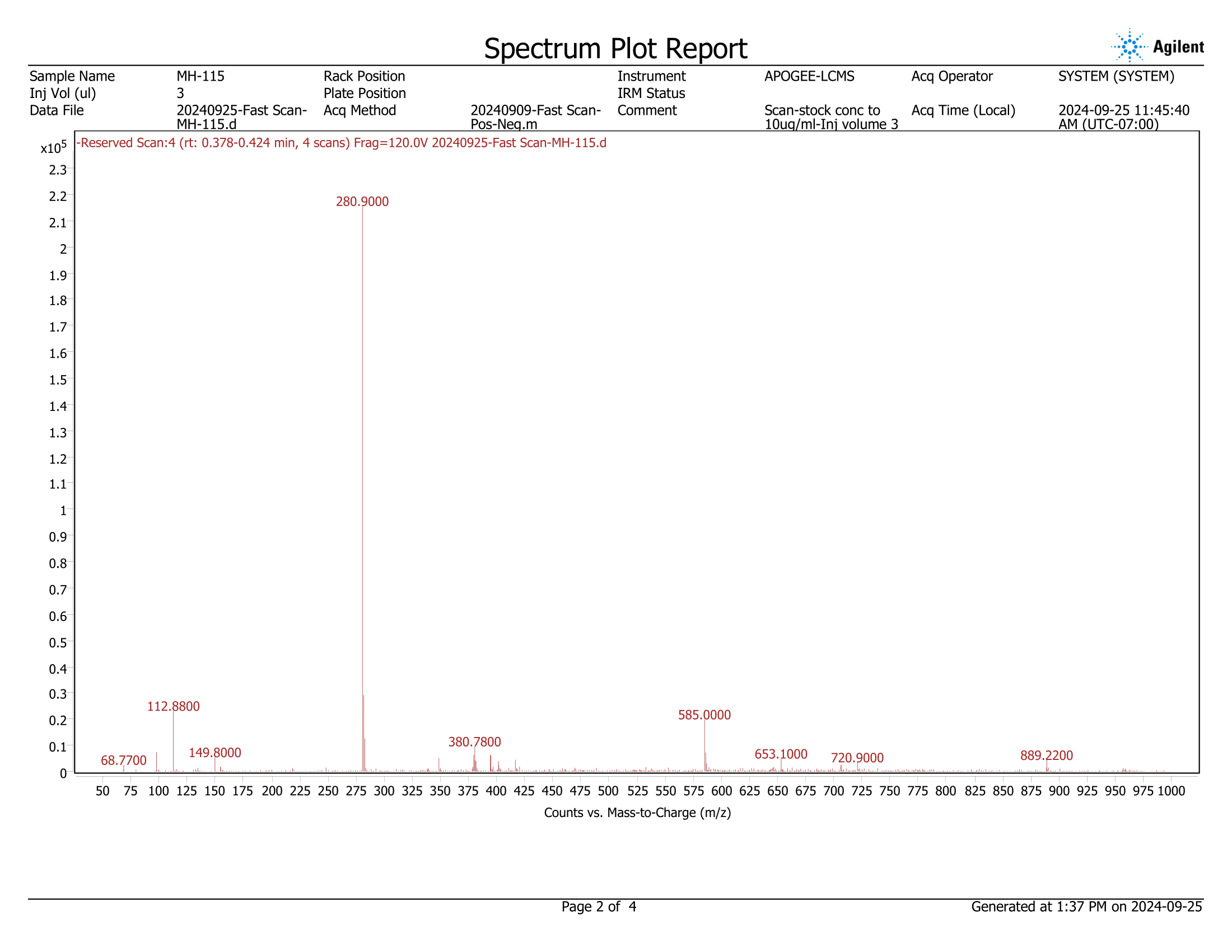
**

**Figure S9**: ^1^H NMR spectrum (400 MHz, DMSO-*d*_6_) of compound **6**

**
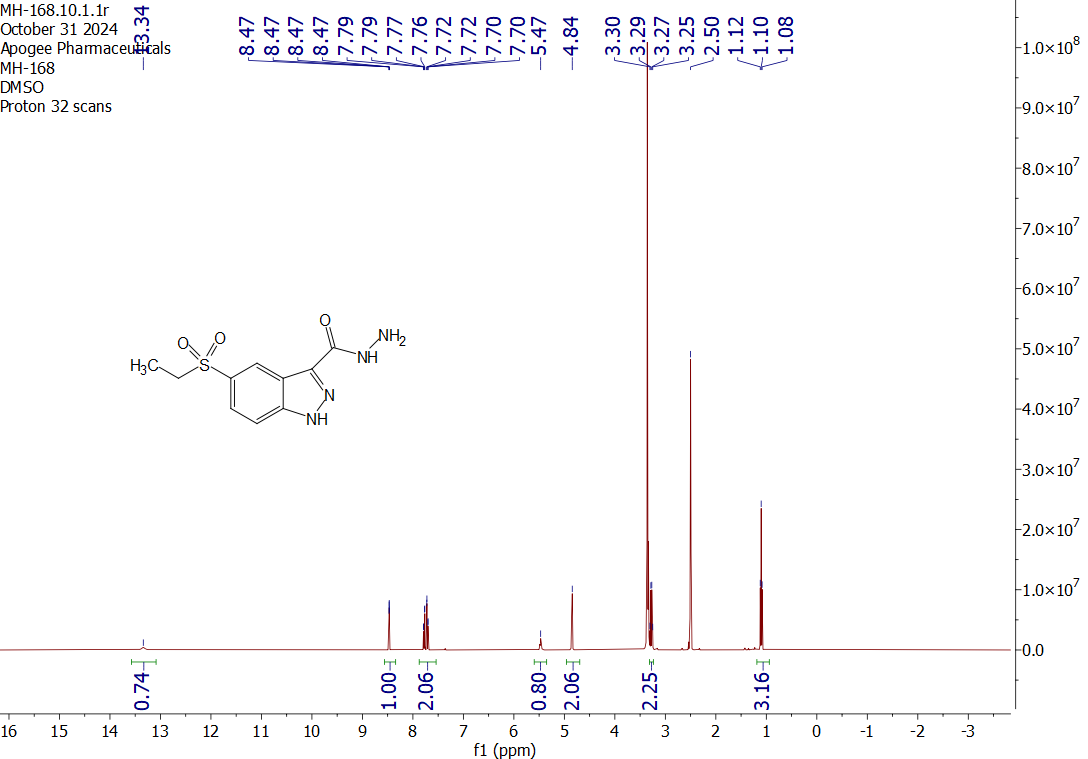
**


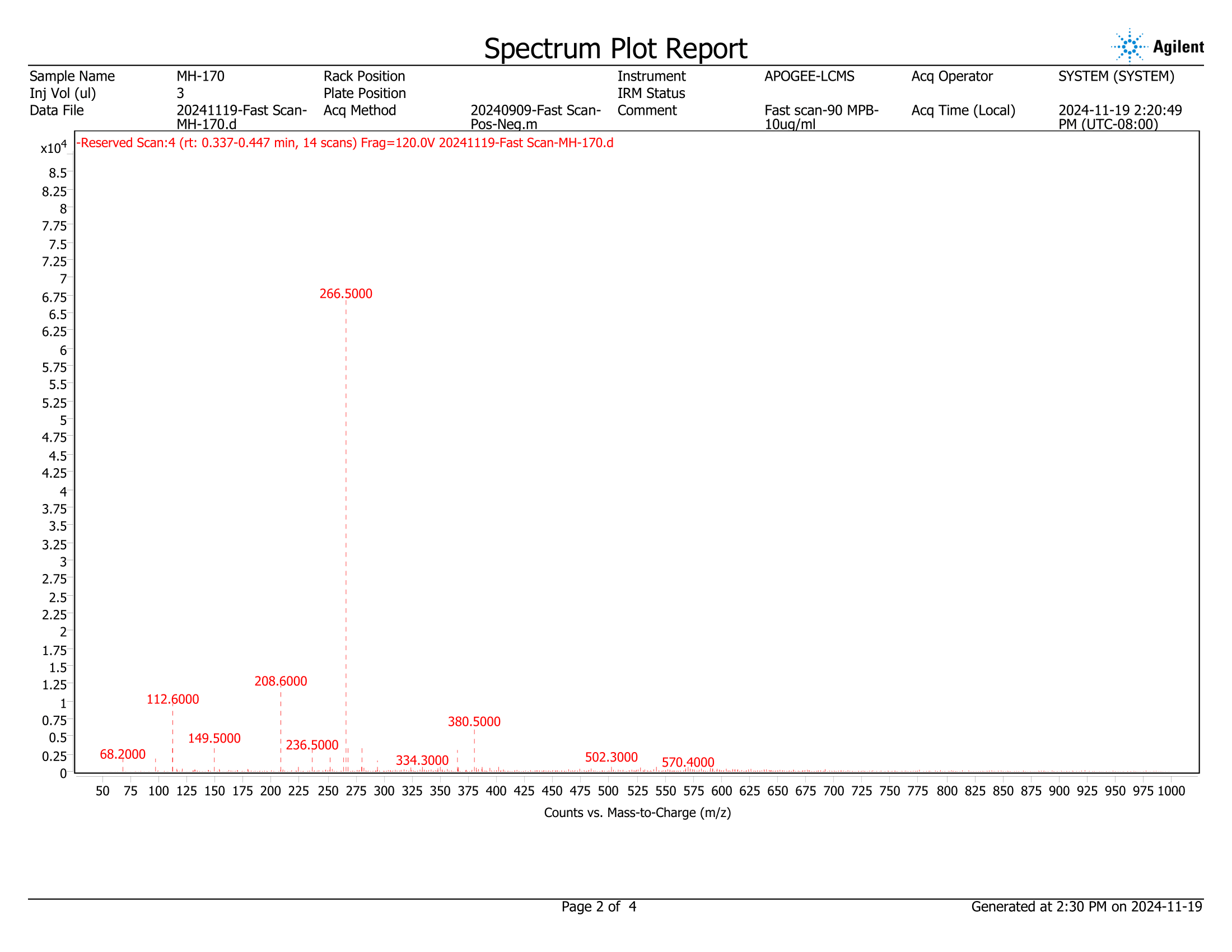
**Figure S10**: HRMS spectrum of compound **6** showing [M-H] peak

**Figure S11**: ^1^H NMR spectrum (500 MHz, DMSO-*d*_6_) of compound **7a**


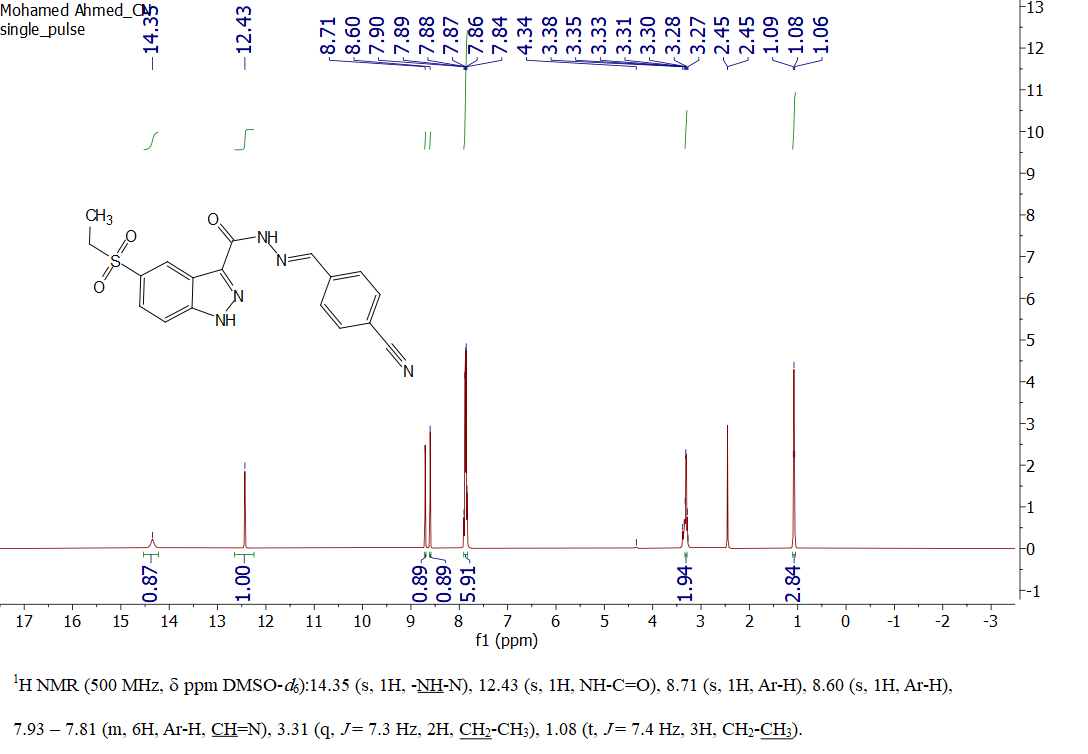


**
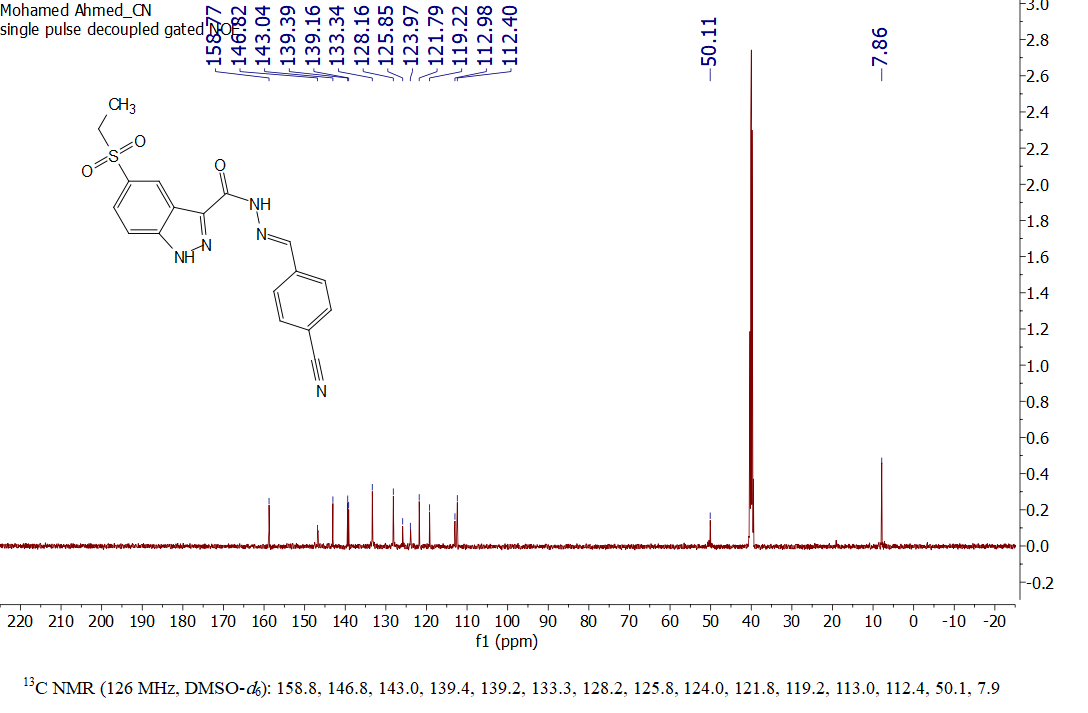
Figure S12**: ^13^C NMR spectrum (126 MHz, DMSO-*d*_6_) of compound **7a**

**Figure S13**: ^1^H NMR spectrum (500 MHz, DMSO-*d*_6_) of compound **7b**

**
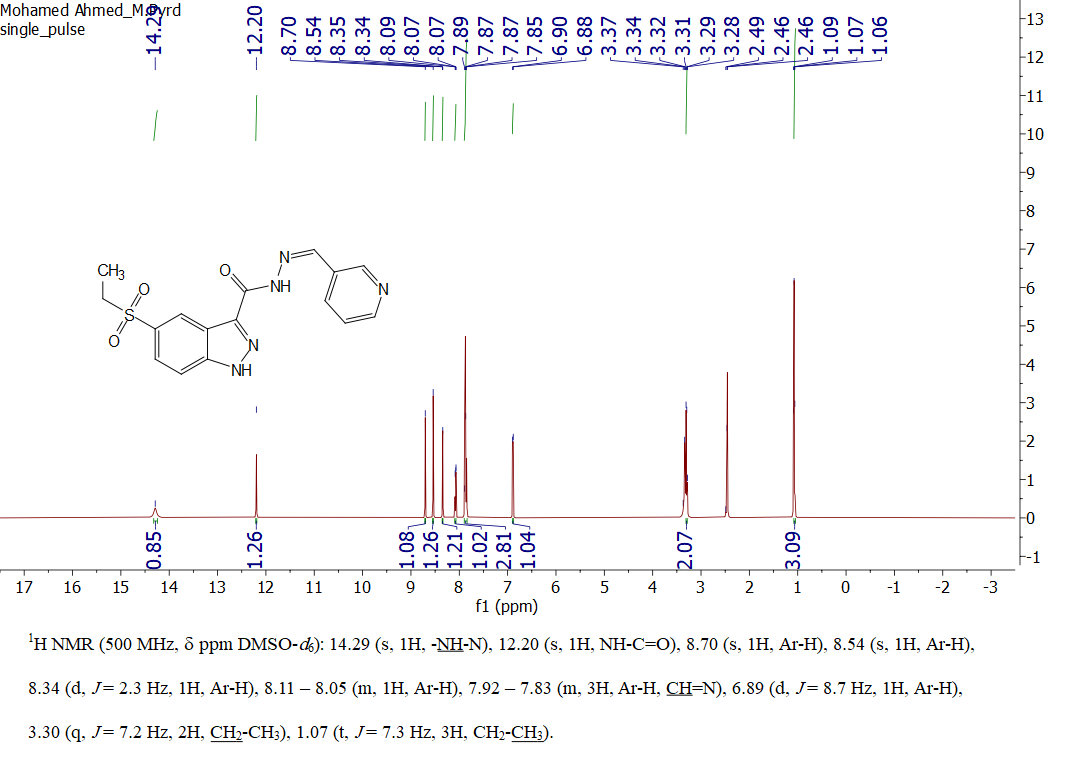
**

**Figure S14**: ^13^C NMR spectrum (126 MHz, DMSO-*d*_6_) of compound **7b**

**
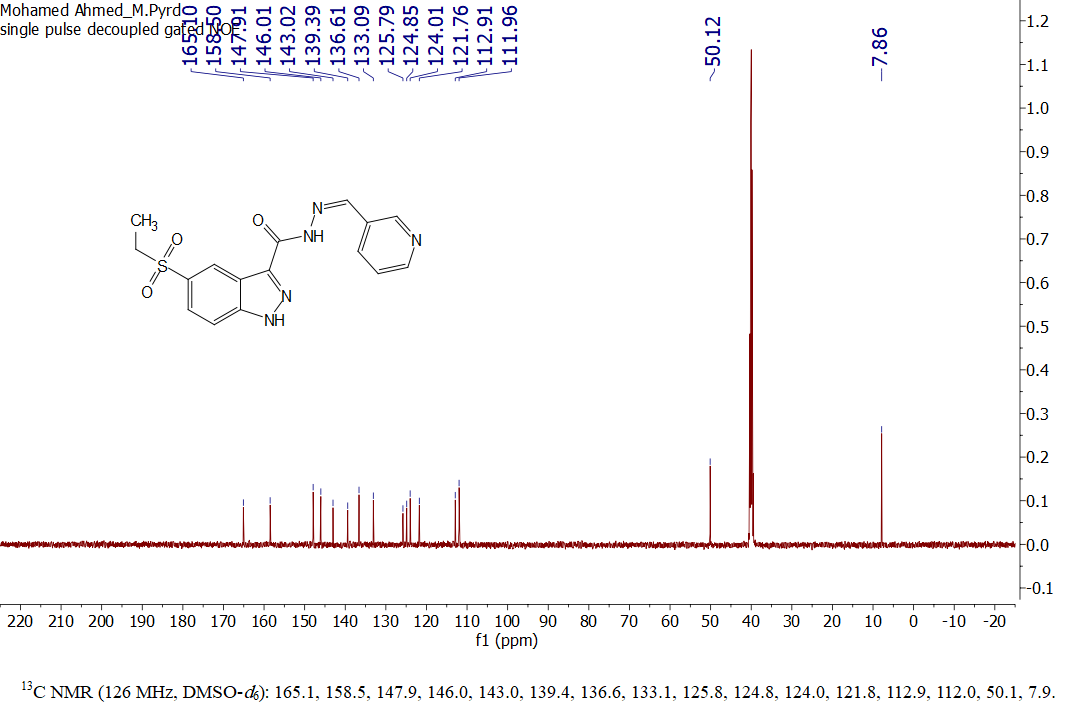
**

**Figure S15**: ^1^H NMR spectrum (500 MHz, DMSO-*d*_6_) of compound **7c**

**
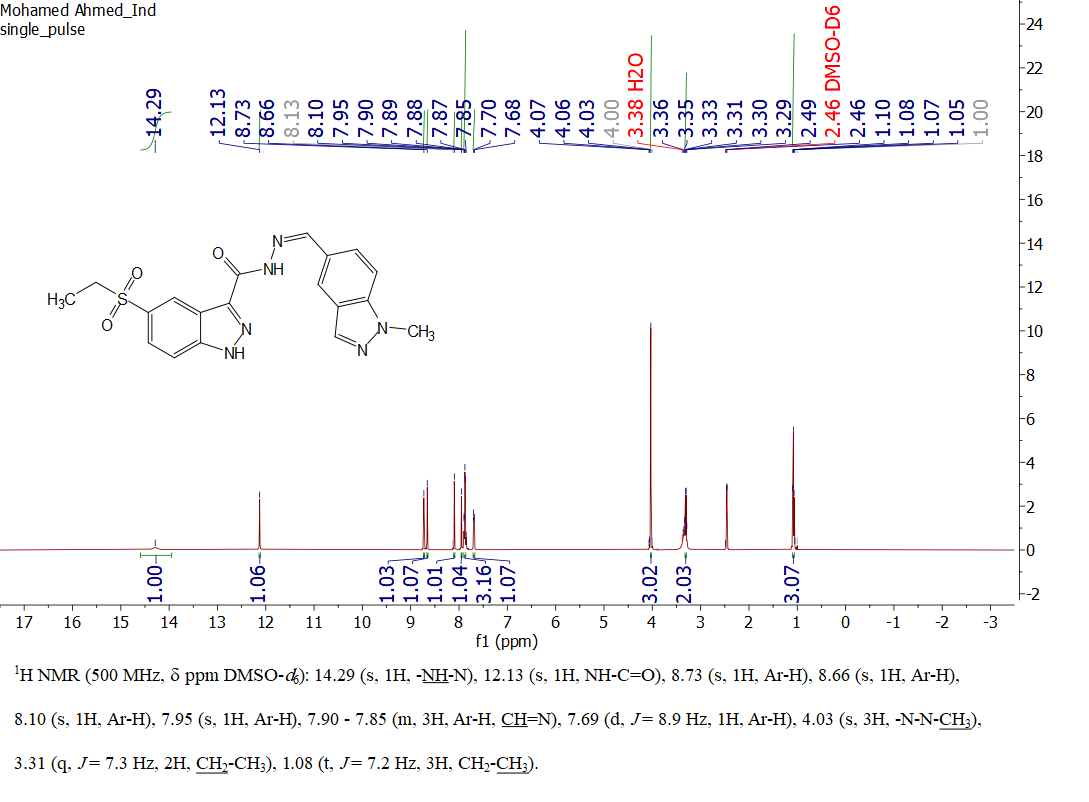
**

**Figure S16**: ^13^C NMR spectrum (126 MHz, DMSO-*d*_6_) of compound **7c**

**
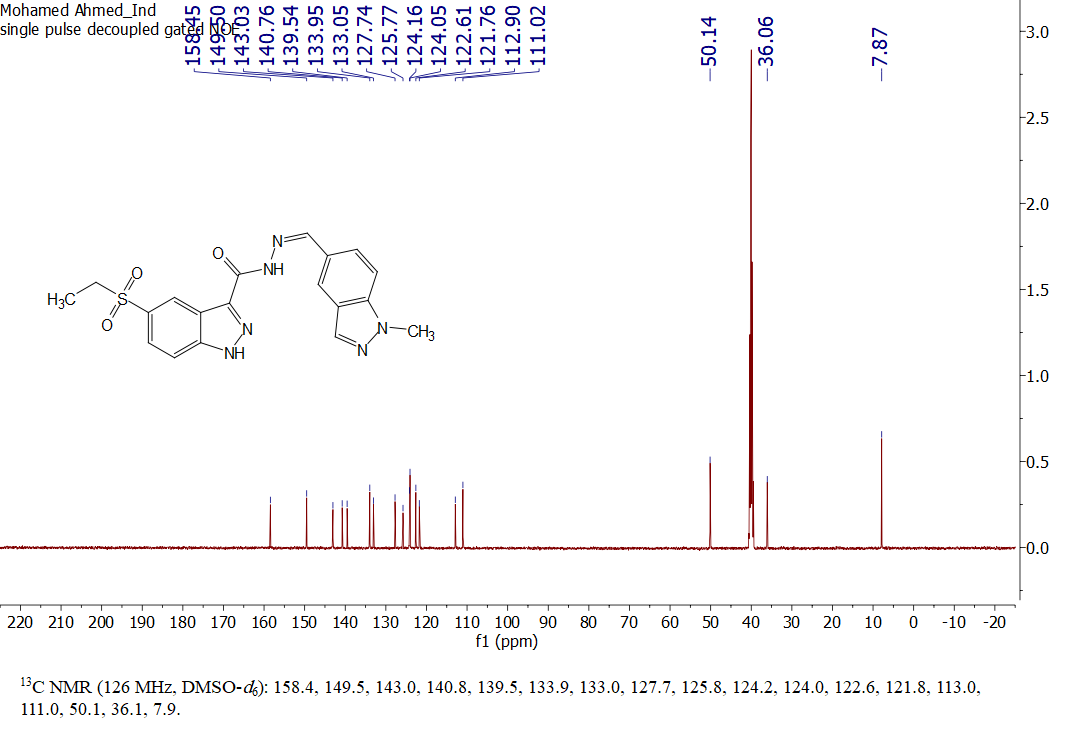
**

**Figure S17**: ^1^H NMR spectrum (500 MHz, DMSO-*d*_6_) of compound **7d**

**
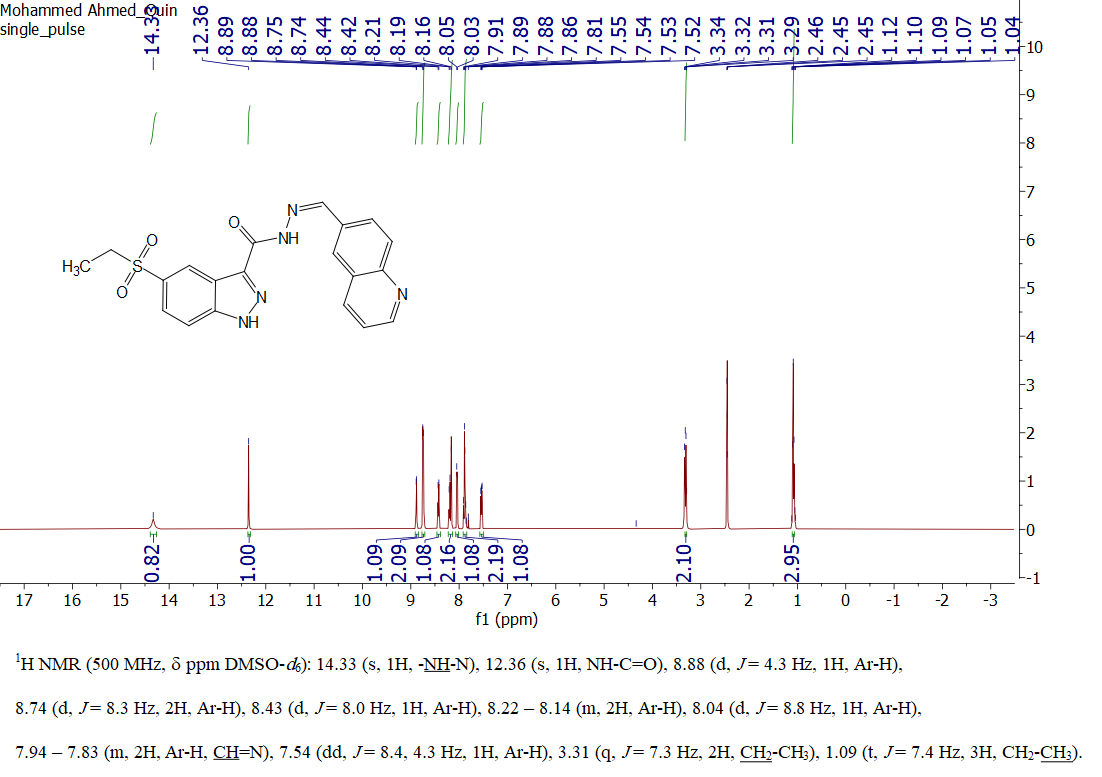
**

**Figure S18**: ^13^C NMR spectrum (126 MHz, DMSO-*d*_6_) of compound **7d**


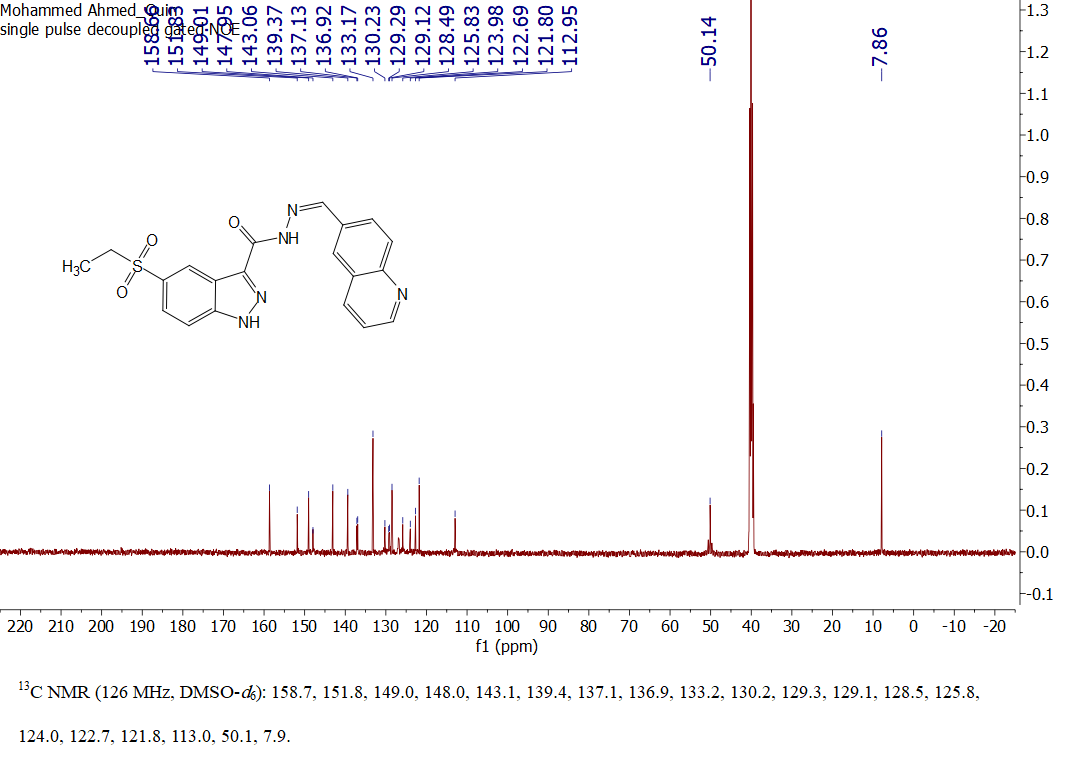


**Figure S19**: ^1^H NMR spectrum (500 MHz, DMSO-*d*_6_) of compound **7e**

**
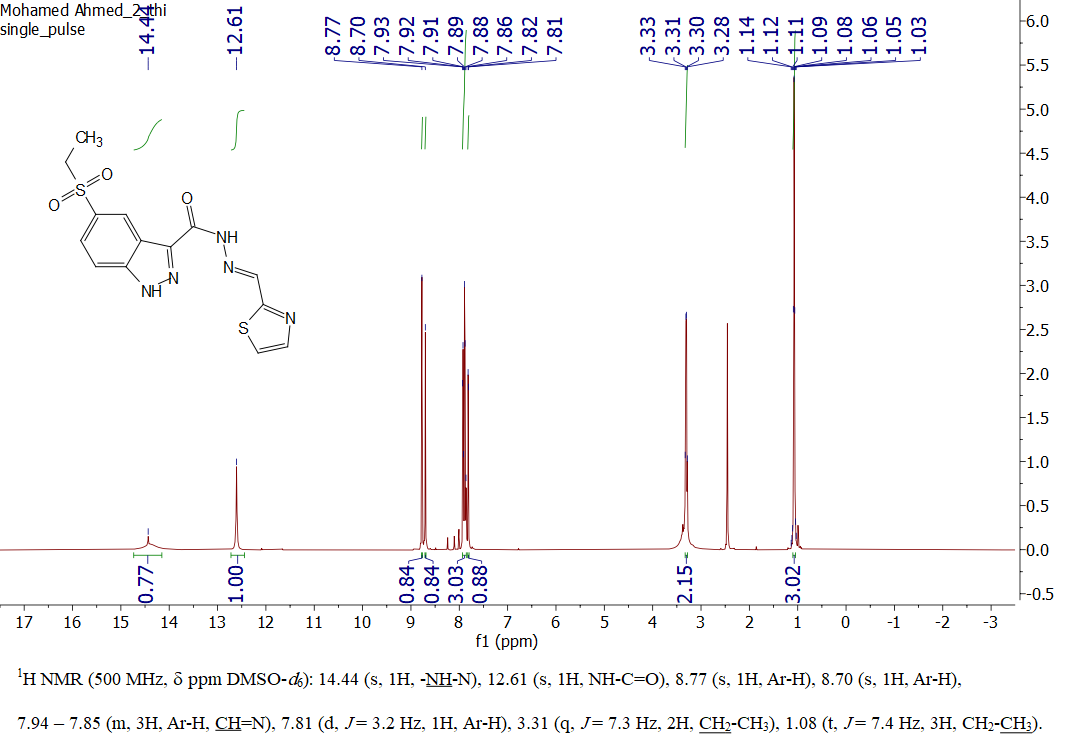
**

**Figure S20**: ^13^C NMR spectrum (126 MHz, DMSO-*d*_6_) of compound **7e**


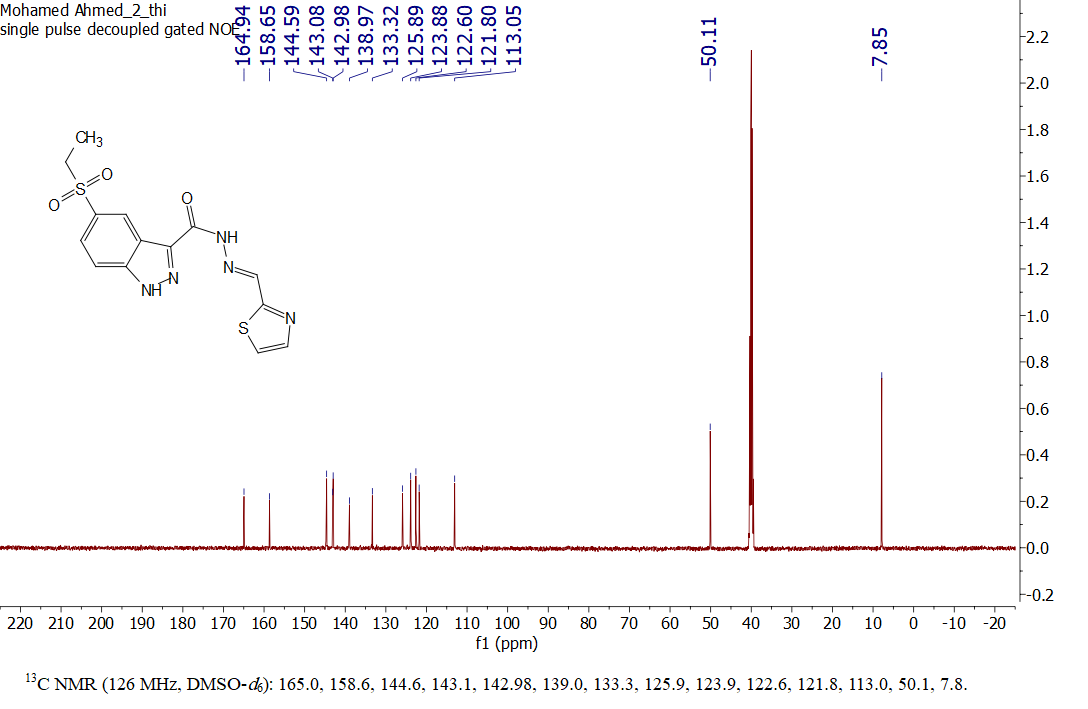


**Figure S21**: ^1^H NMR spectrum (500 MHz, DMSO-*d*_6_) of compound **7f**

**
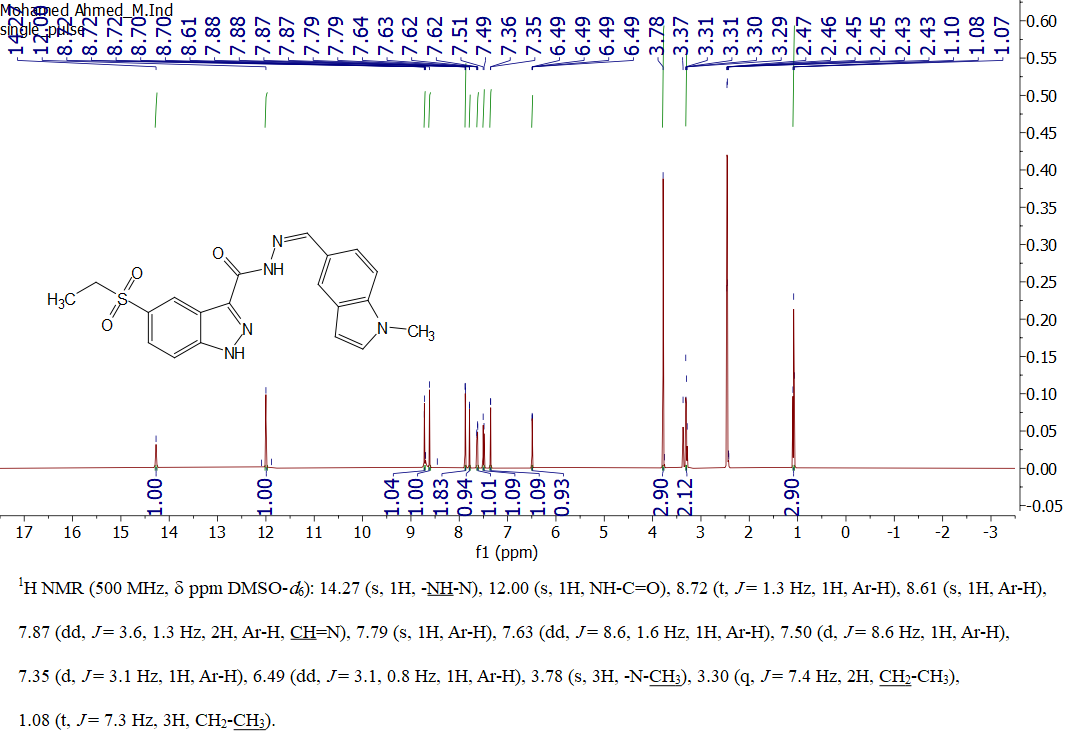
**

**Figure S22**: ^13^C NMR spectrum (126 MHz, DMSO-*d*_6_) of compound **7f**


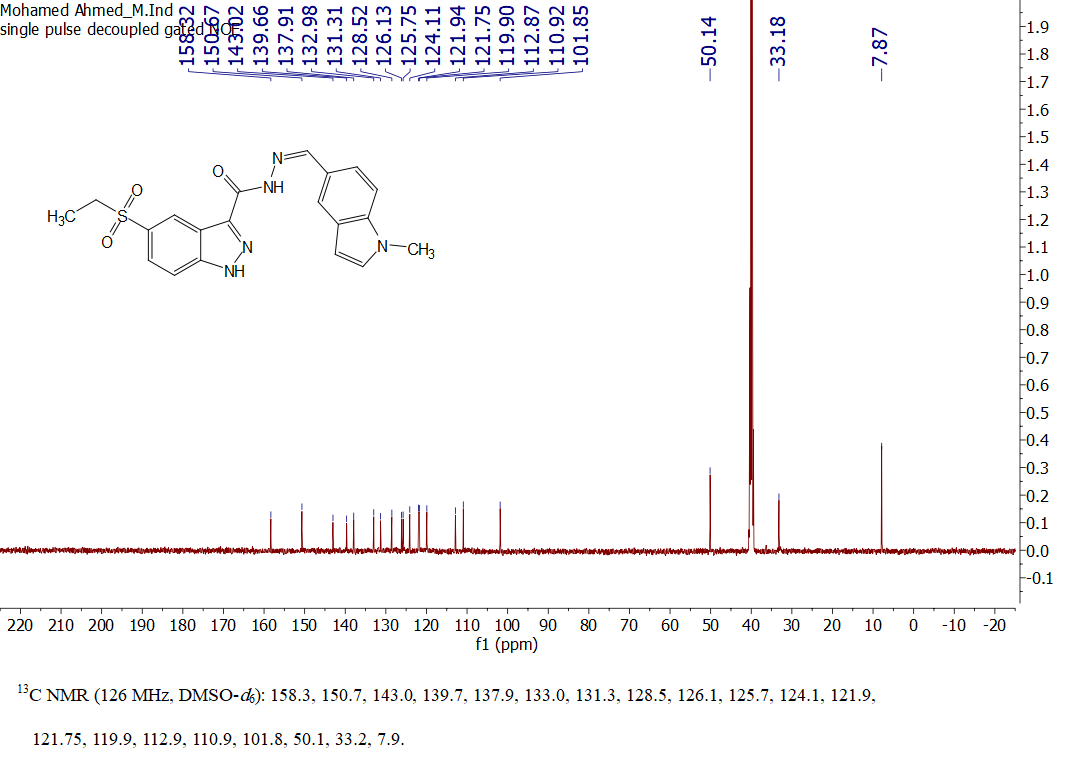


**Figure S23**: ^1^H NMR spectrum (500 MHz, DMSO-*d*_6_) of compound **7g**

**
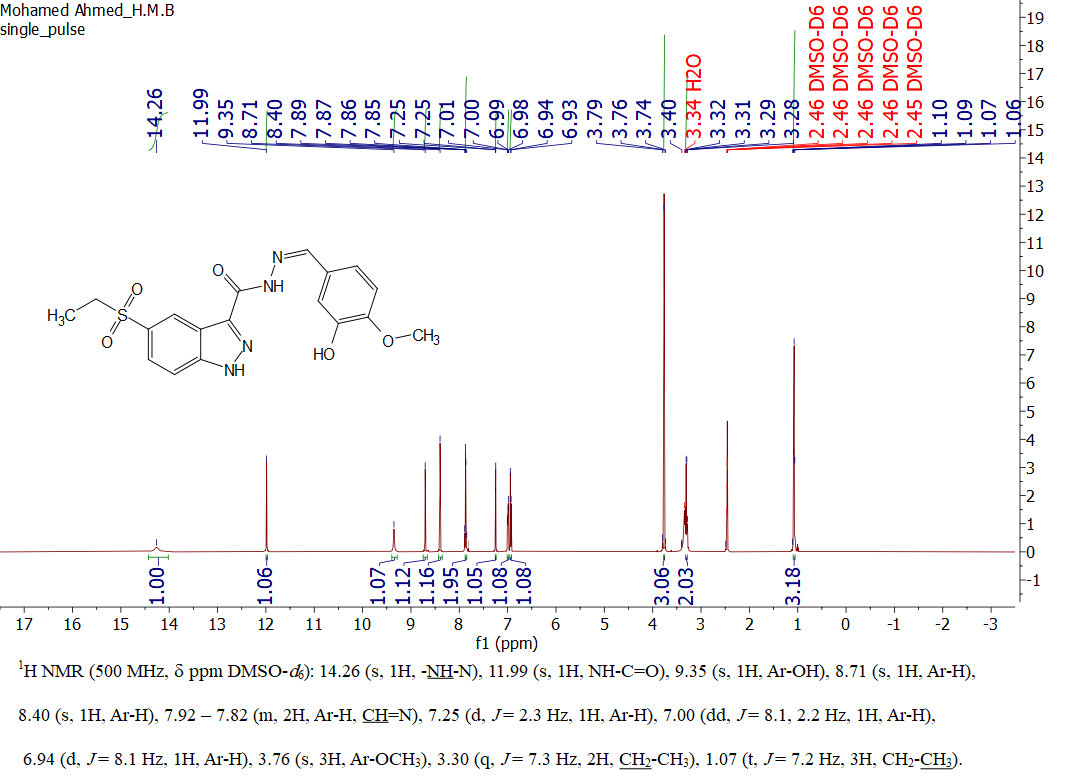
**

**Figure S24**: ^13^C NMR spectrum (126 MHz, DMSO-*d*_6_) of compound **7g**

**
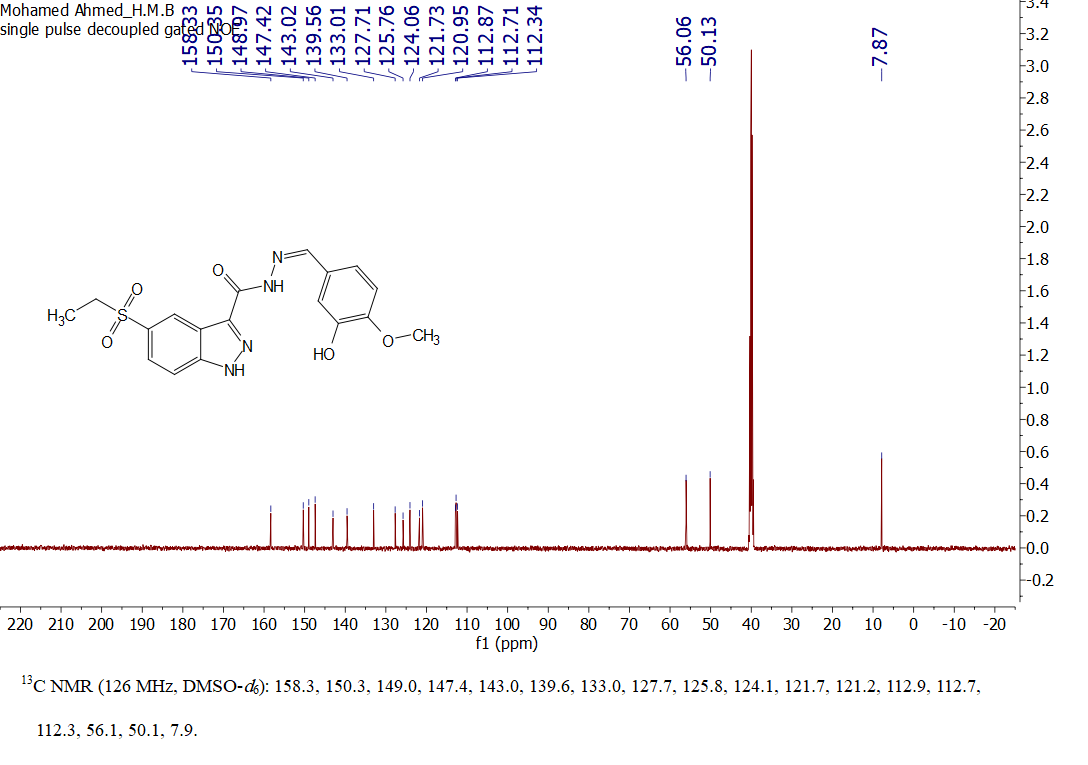
**

**Figure S25**: ^1^H NMR spectrum (500 MHz, DMSO-*d*_6_) of compound **7h**

**
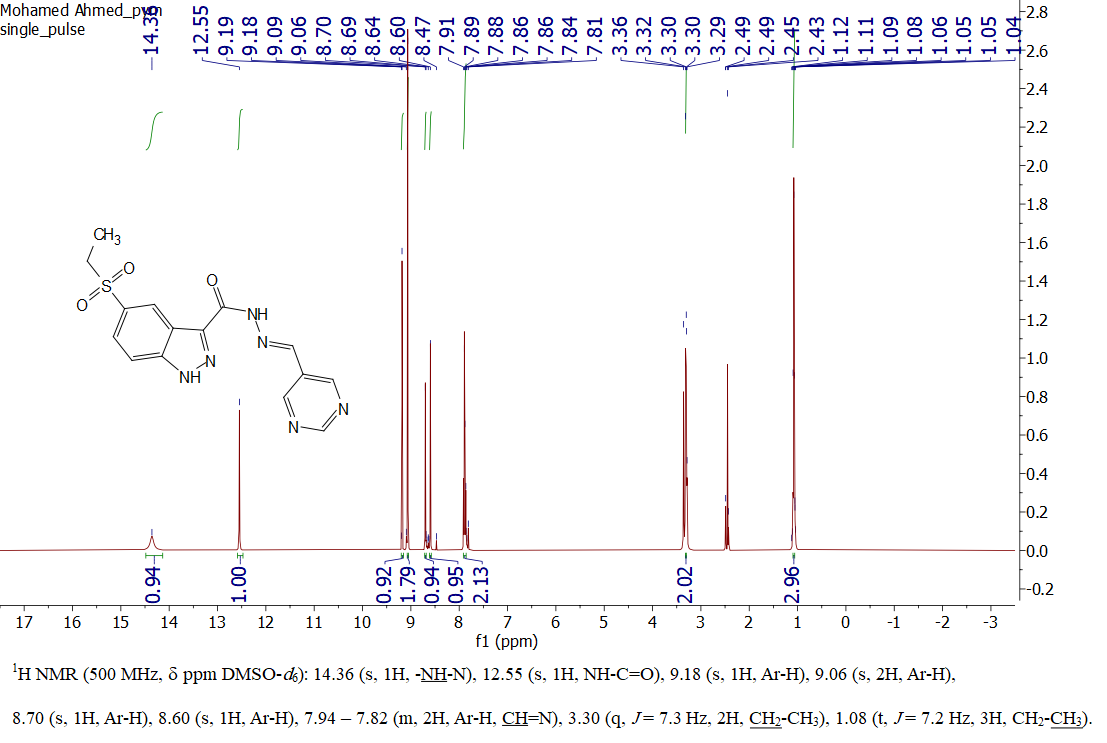
**

**Figure S26**: ^1^H NMR spectrum (500 MHz, DMSO-*d*_6_) of compound **7i**

**
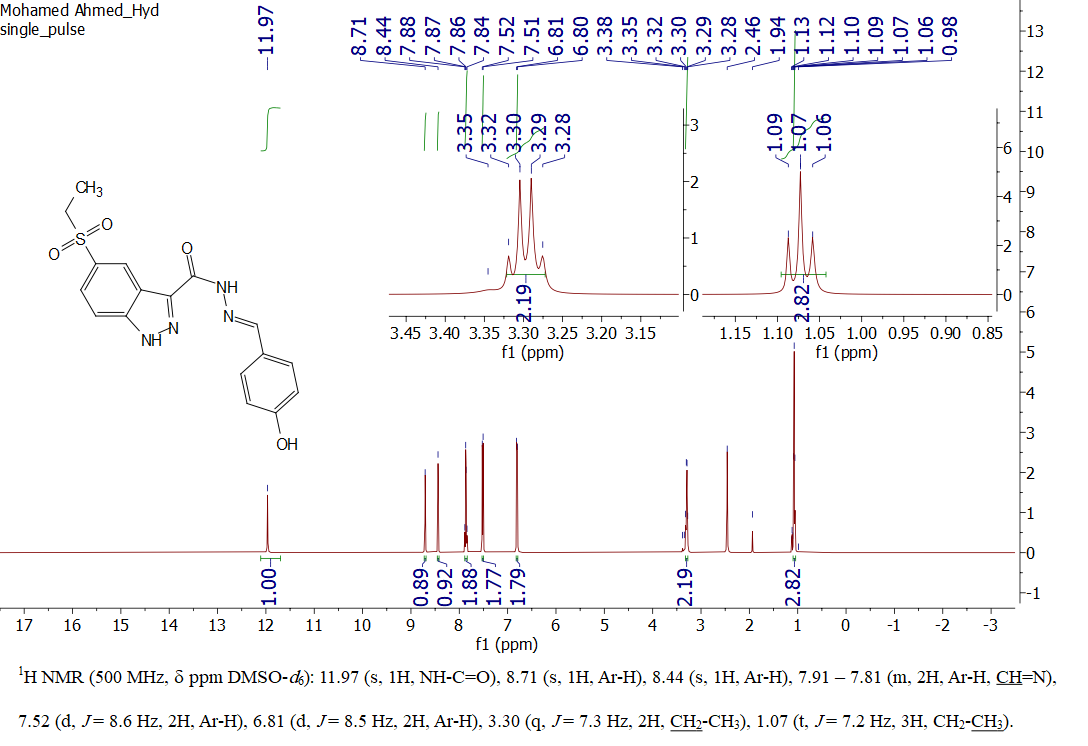
**

**Figure S27**: ^13^C NMR spectrum (126 MHz, DMSO-*d*_6_) of compound **7i**

**
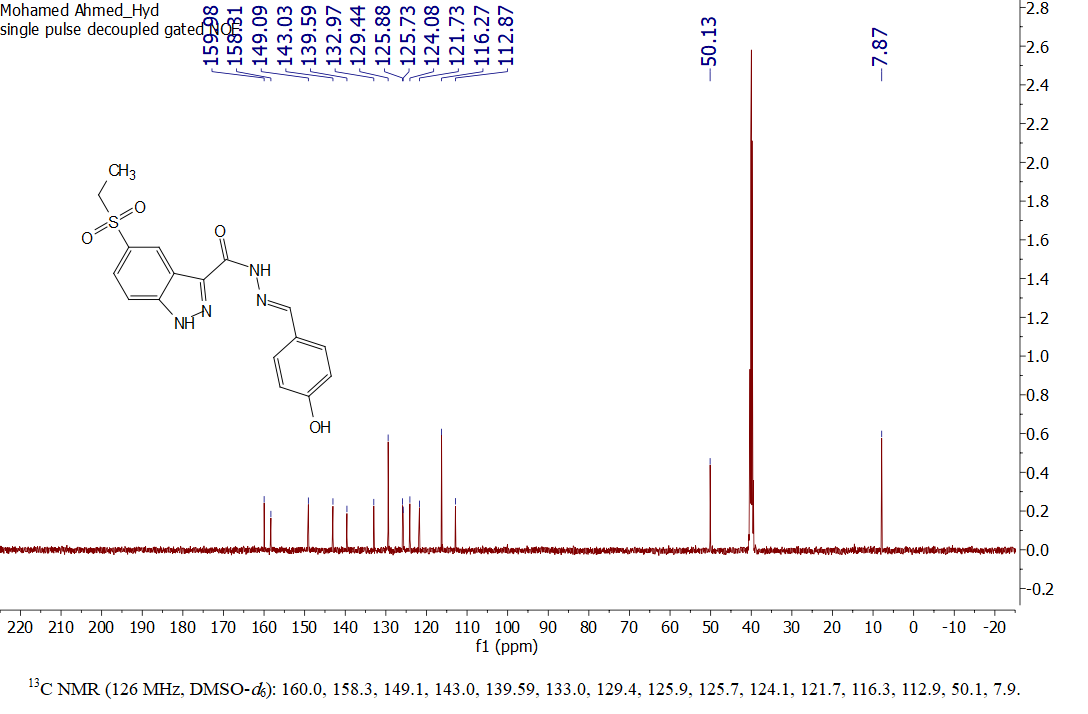
**

**Figure S28**: ^1^H NMR spectrum (500 MHz, DMSO-*d*_6_) of compound **7j**

**
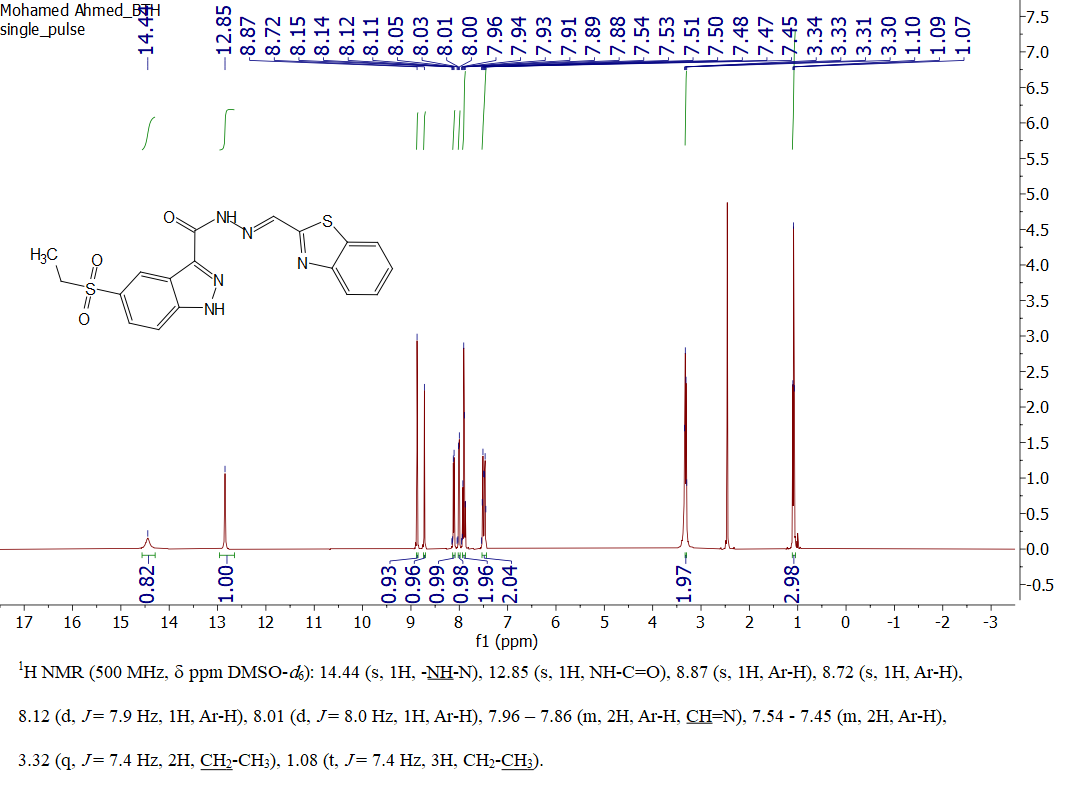
**

**Figure S29**: ^13^C NMR spectrum (126 MHz, DMSO-*d*_6_) of compound **7j**

**
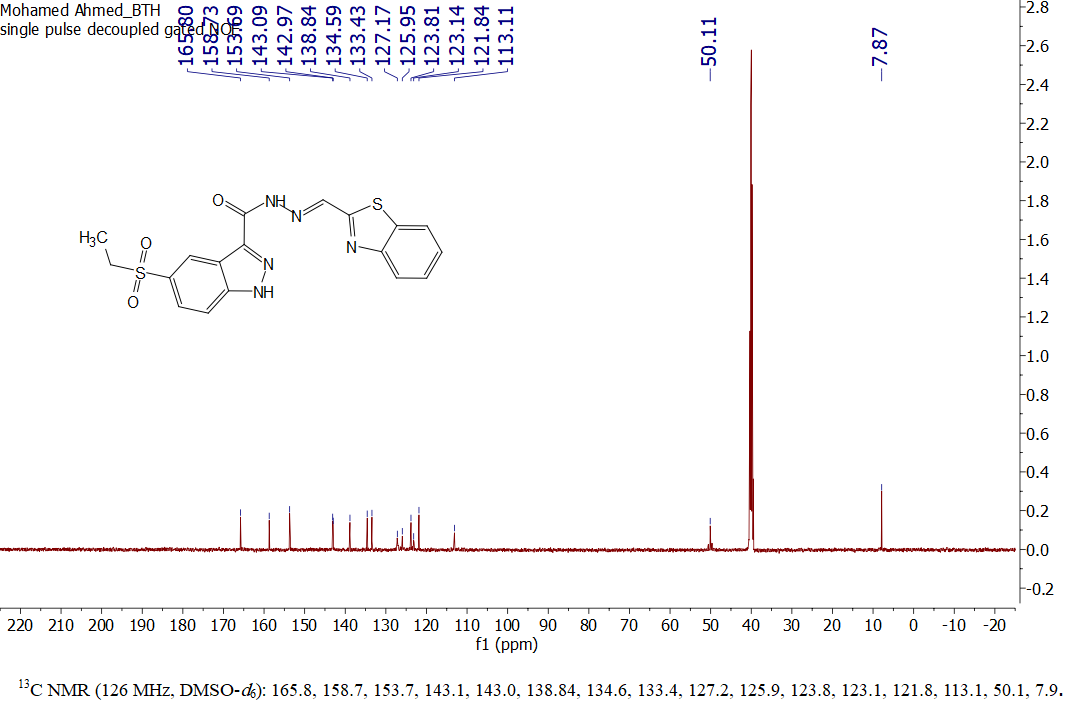
**

**Figure S30**: ^1^H NMR spectrum (500 MHz, DMSO-*d*_6_) of compound **7k**

**
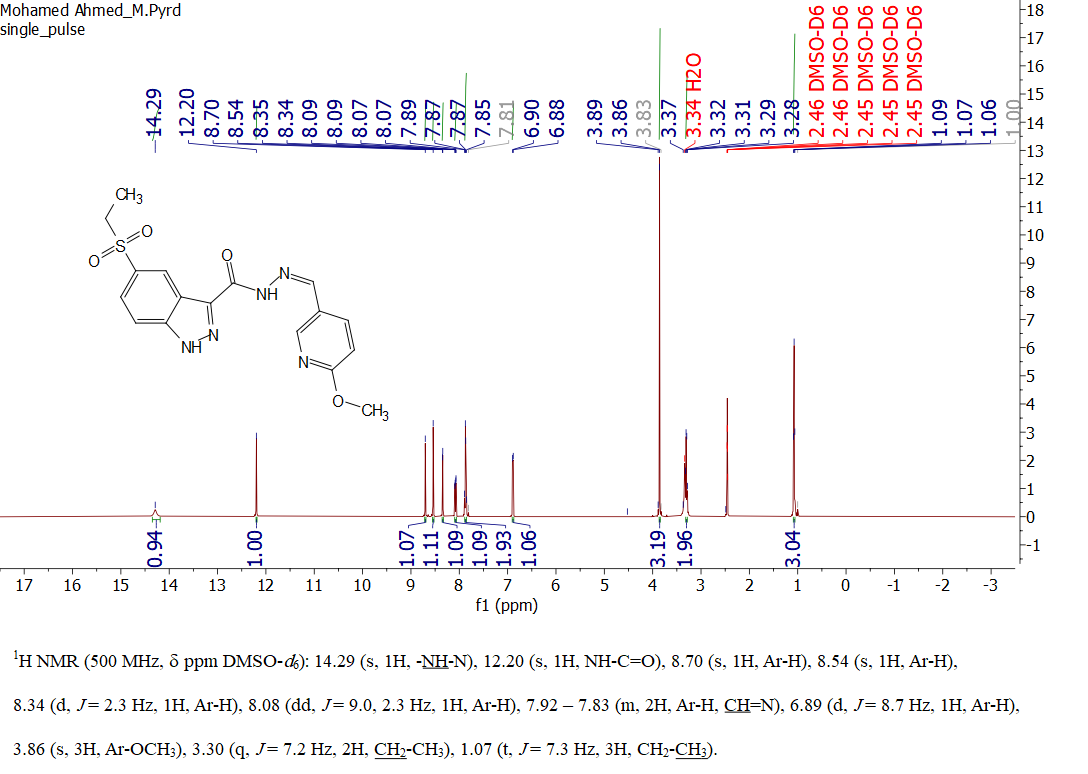
**

**Figure S31**: ^13^C NMR spectrum (126 MHz, DMSO-*d*_6_) of compound **7k**

**
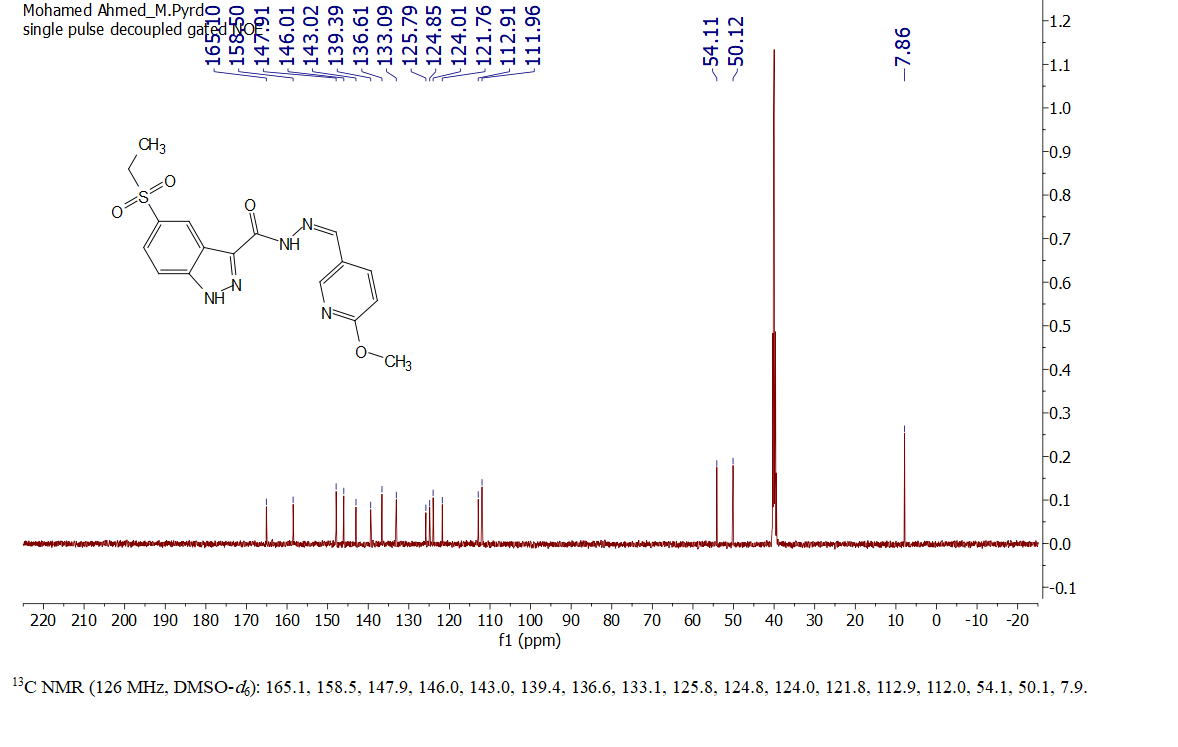
**

**Figure S32**: ^1^H NMR spectrum (500 MHz, DMSO-*d*_6_) of compound **7l**

**
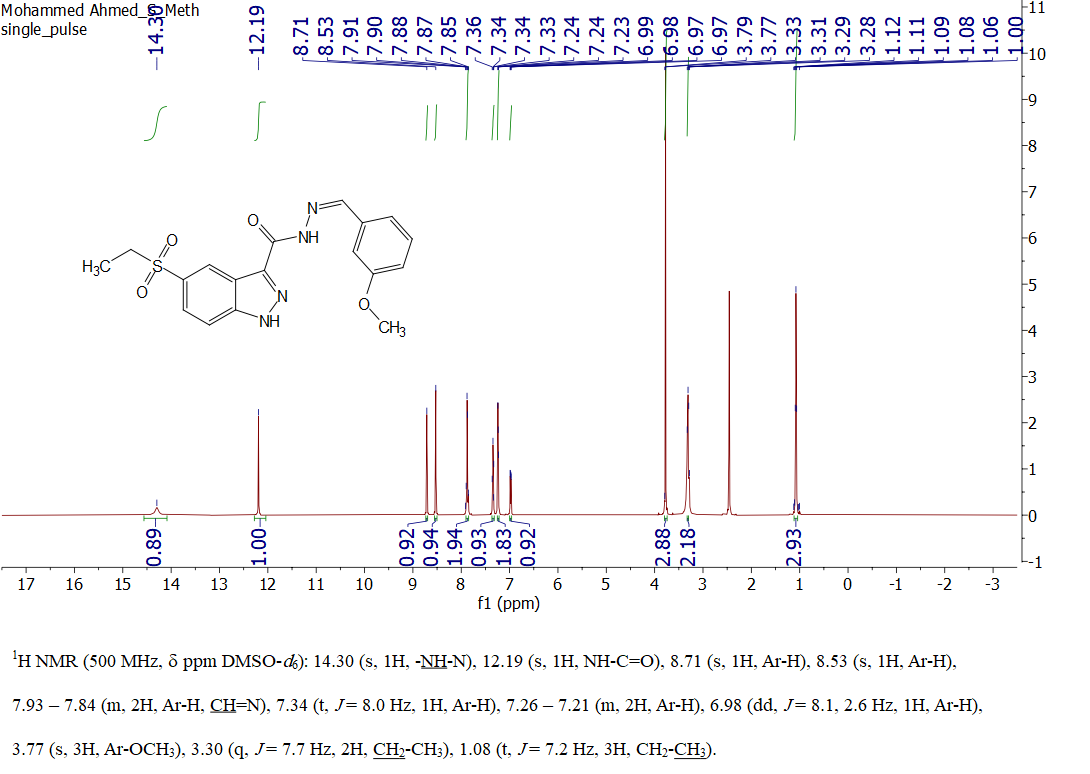
**

**Figure S33**: ^13^C NMR spectrum (126 MHz, DMSO-*d*_6_) of compound **7l**

**
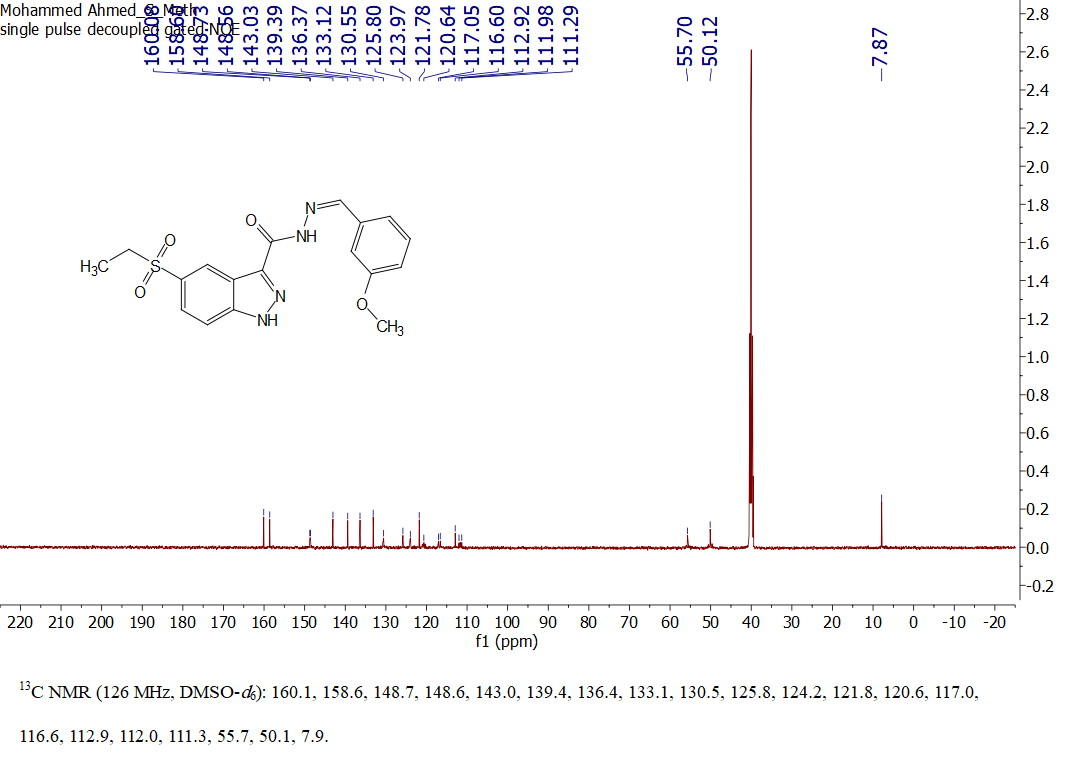
**

**Figure S34**: ^1^H NMR spectrum (500 MHz, DMSO-*d*_6_) of compound **7m**

**
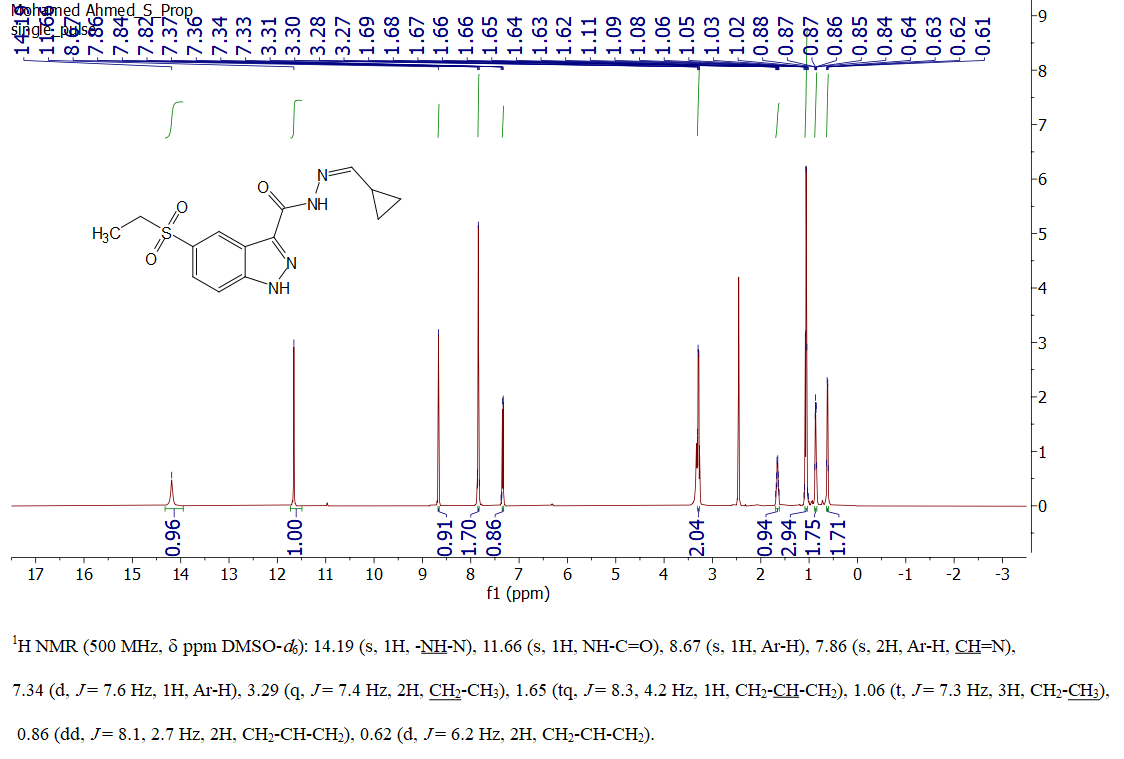
**

**Figure S35**: ^13^C NMR spectrum (126 MHz, DMSO-*d*_6_) of compound **7m**

**
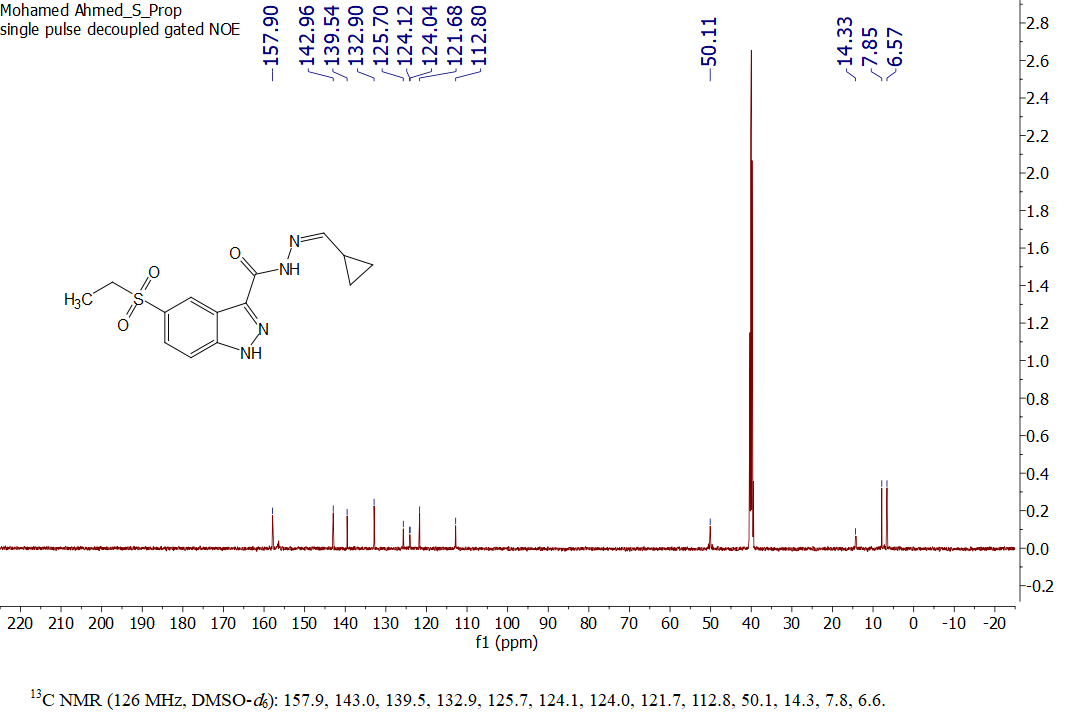
**

**Figure S36**: ^1^H NMR spectrum (500 MHz, DMSO-*d*_6_) of compound **7n**

**
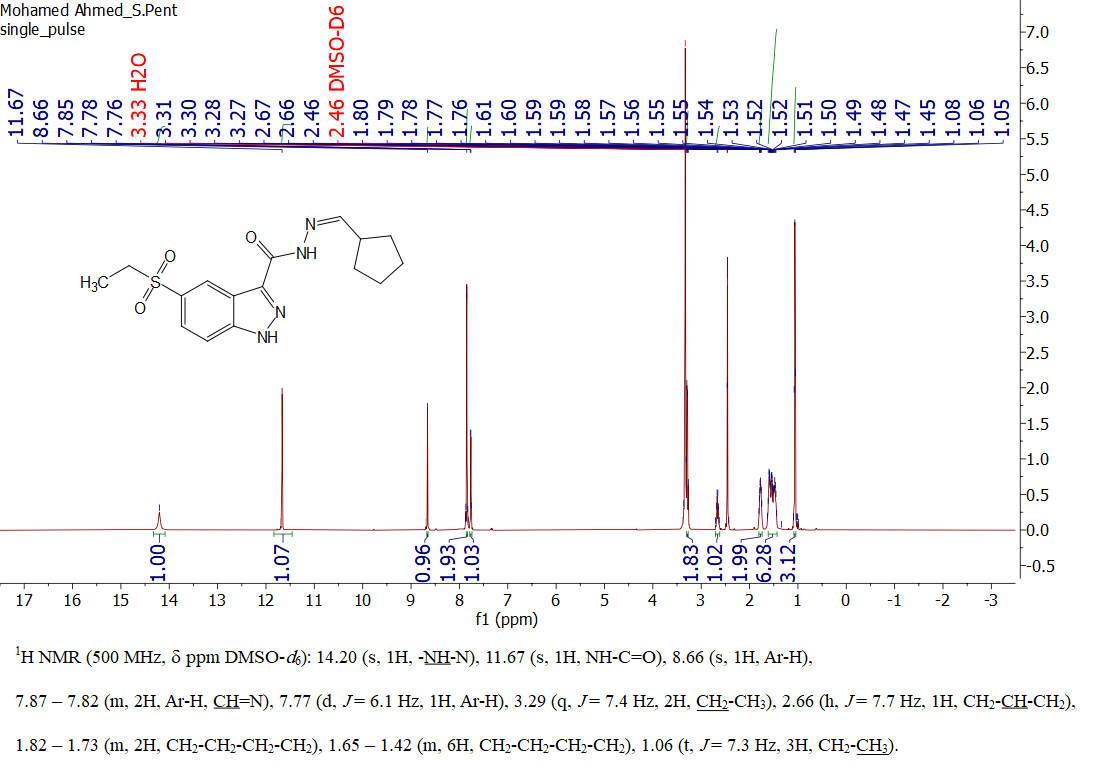
**

**Figure S37**: ^13^C NMR spectrum (126 MHz, DMSO-*d*_6_) of compound **7n**

**
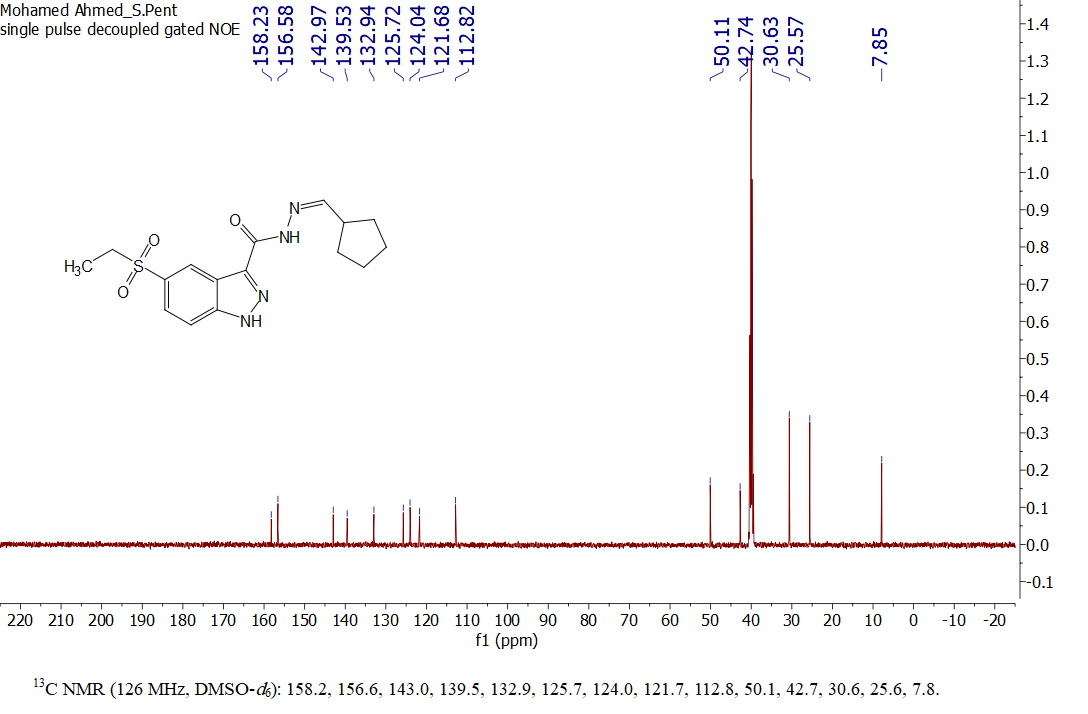
**

**Figure S38**: ^1^H NMR spectrum (500 MHz, DMSO-*d*_6_) of compound **7o**

**
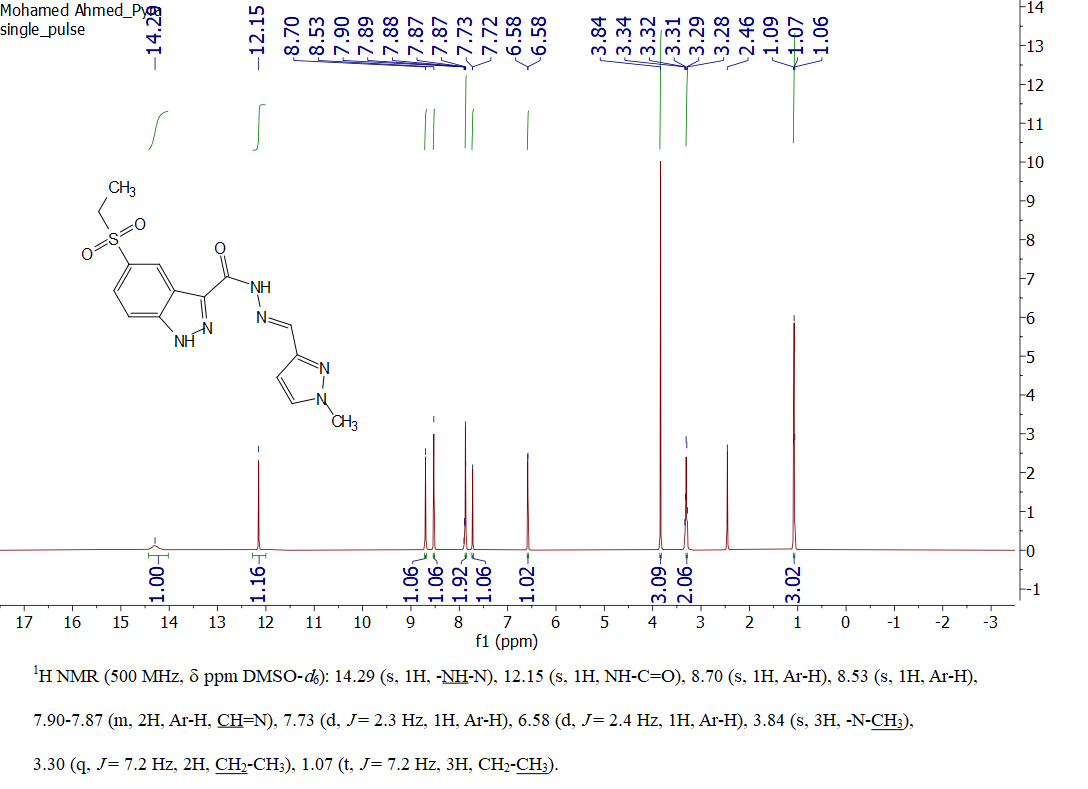
**

**Figure S39**: ^13^C NMR spectrum (126 MHz, DMSO-*d*_6_) of compound **7o**

**
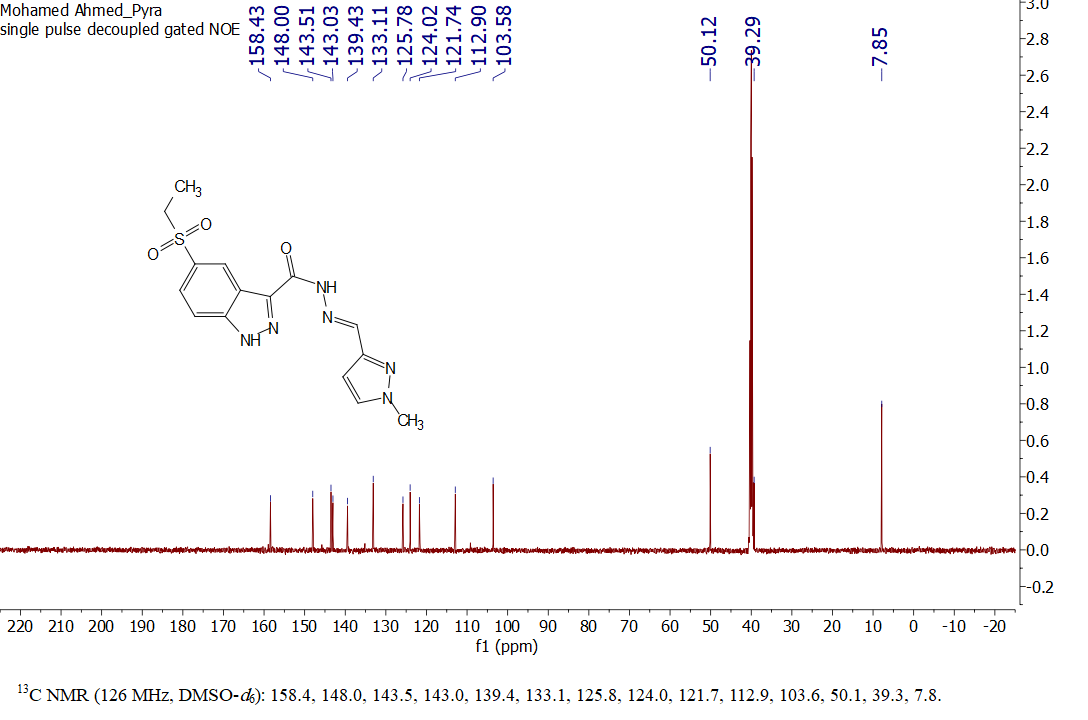
**

**Appendix A**

**4. EXPERIMENTAL**

**4.1. Chemistry**

**Materials and methods**

All reagents and solvents were of general purpose or analytical grade and purchased from Sigma Aldrich Ltd, Fisher Scientific, Fluka and Acros. ^1^H and ^13^C NMR spectra were recorded with a Bruker Avance III spectrometer operating at 400, 100 MHz respectively, with Me4Si as internal standard and DMSO-d6 as a solvent. Elemental analysis was performed by the regional center for mycology and biotechnology (Cairo, Egypt). TLC was carried out on precoated silica plates (Keisel gel 60 F254, BDH) using Hexane: Ethyl acetate, 1 : 2, v/v. Compounds were visualized by illumination under UV light (254 nm). Melting points were determined on an electrothermal instrument and are uncorrected. All solvents were dried prior to use and stored over 4 Å molecular sieves, under nitrogen. All the compounds were ≥ 95% pure.

**4.2. Biological evaluation**

**4.2.1 Cell Viability assay (MTT assay)**

MTT assay was performed to investigate the effect of the synthesized compounds on mammary epithelial cells (MCF-10A). The cells were propagated in medium consisting of Ham's F-12 medium/ Dulbecco's modified Eagle's medium (DMEM) (1:1) supplemented with 10% foetal calf serum (GIBCO, UK), 2 mM glutamine, insulin (10 μg/mL), hydrocortisone (500 ng/mL) and epidermal growth factor (20 ng/mL). Trypsin ethylenediamine tetra acetic acid (EDTA) was used to passage the cells after every 2-3 days. 96-well flat-bottomed cell culture plates were used to seed the cells at a density of 104 cells mL^-1^. The medium was aspirated from all the wells of culture plates after 24 h followed by the addition of synthesized compounds (in 200 μL medium to yield a final concentration of 0.1% (v/v) dimethyl sulfoxide) into individual wells of the plates. Four wells were designated to a single compound. The plates were allowed to incubate at 37°C for 96 h. Afterwards, the medium was aspirated and 3-[4,5-dimethylthiazol-2-yl]-2,5-diphenyltetrazolium bromide (MTT) (0.4 mg/mL) in medium was added to each well and subsequently incubated for 3 h. The medium was aspirated and 150 μL dimethyl sulfoxide (DMSO) was added to each well. The plates were vortexed followed by the measurement of absorbance at 540 nm on a microplate reader. The results were presented as inhibition (%) of proliferation in contrast to controls comprising 0.1% DMSO.

**4.2.2. Assay for antiproliferative effect**

To explore the antiproliferative potential of compounds propidium iodide fluorescence assay was performed using different cell lines such as Panc-1 (pancreas cancer cell line) [Panc-1-CTC (RRID:CVCL_VQ69)], MCF-7 (breast cancer cell line) [MCF-7 (RRID:CVCL_0031)], HT-29 (colon cancer cell line) [HT-29 (RRID:CVCL_0320)] and A-549 (epithelial cancer cell line)[A-549 (RRID:CVCL_0023)], respectively. All cell lines were obtained from ATCC (American Type Cell Culture).

To calculate the total nuclear DNA, a fluorescent dye (propidium iodide, PI) is used which can attach to the DNA, thus offering a quick and precise technique. PI cannot pass through the cell membrane and its signal intensity can be considered as directly proportional to quantity of cellular DNA. Cells whose cell membranes are damaged or have changed permeability are counted as dead ones. The assay was performed by seeding the cells of different cell lines at a density of 3000-7500 cells/well (in 200 μl medium) in culture plates followed by incubation for 24 h at 37 °C in humidified 5% CO2 /95% air atmospheric conditions. The medium was removed; the compounds were added to the plates at 10 μM concentrations (in 0.1% DMSO) in triplicates, followed by incubation for 48 h. DMSO (0.1%) was used as control. After incubation, medium was removed followed by the addition of PI (25 μl, 50 μg/mL in water/medium) to each well of the plates. At -80 °C, the plates were allowed to freeze for 24 h, followed by thawing at 25 ^o^C. A fluorometer (Polar-Star BMG Tech) was used to record the readings at excitation and emission wavelengths of 530 and 620 nm for each well. The percentage cytotoxicity of compounds was calculated using the following formula:

Where A*TC*= Absorbance of treated cells and AC= Absorbance of control. Erlotinib was used as positive control in the assay.

**4.2.3. EGFR inhibitory assay**

Baculoviral expression vectors including pBlueBacHis2B and pFASTBacHTc were used separately to clone 1.6 kb cDNA coding for EGFR cytoplasmic domain (EGFR-CD, amino acids 645–1186). 5ʹ upstream to the EGFR sequence comprised a sequence that encoded (His)6. Sf-9 cells were infected for 72h for protein expression. The pellets of Sf-9 cells were solubilized in a buffer containing sodium vanadate (100 μM), aprotinin (10 μg/mL), triton (1%), HEPES buffer(50mM), ammonium molybdate (10 μM), benzamidine HCl (16 μg/mL), NaCl (10 mM),leupeptin (10 μg/mL) and pepstatin (10 μg/mL) at 0°C for 20 min at pH 7.4, followed by centrifugation for 20 min. To eliminate the nonspecifically bound material, a Ni-NTA super flow packed column was used to pass through and wash the crude extract supernatant first with 10mM and then with 100 mM imidazole. Histidine-linked proteins were first eluted with 250 and then with 500 mM imidazole subsequent to dialysis against NaCl (50 mM), HEPES (20 mM), glycerol (10%) and 1 μg/mL each of aprotinin, leupeptin and pepstatin for 120 min. The purification was performed either at 4 °C or on ice. To record autophosphorylation level, EGFR kinase assay was carried out on the basis of DELFIA/Time-Resolved Fluorometry. The compounds were first dissolved in DMSO absolute, subsequent to dilution to appropriate concentration using HEPES (25 mM) at pH 7.4. Each compound (10 μL) was incubated with recombinant enzyme (10 μL, 5 ng for EGFR, 1:80 dilution in 100 mM HEPES) for 10 min at 25^o^C, subsequent to the addition of 5X buffer (10 μL, containing 2 mM MnCl2, 100 μM Na3VO4, 20 mM HEPES and 1 mM DTT) and ATP-MgCl2 (20 μL, containing 0.1 mM ATP and 50 mM MgCl2) and incubation for 1h. The negative and positive controls were included in each plate by the incubation of enzyme either with or without ATP-MgCl2. The liquid was removed after incubation and the plates were washed thrice using a wash buffer. The Europium-tagged antiphosphotyrosine antibody (75 μL, 400 ng) was added to each well followed by incubation of 1h and then washing of the plates using buffer. The enhancement solution was added to each well and the signal was recorded at excitation and emission wavelengths of 340 at 615 nm. The autophosphorylation percentage inhibition by compounds was calculated using the following equation:

Using the curves of percentage inhibition of eight concentrations of each compound, IC50 was calculated. The majority of signals detected by antiphosphotyrosine antibody were from EGFR because the enzyme preparation contained low impurities.

**4.2.4. VEGFR-2 inhibitory assay**

Prepare all the solutions using autoclaved, deionized water and analytical grade reagents. Prepare and store all the reagents at room temperature (unless indicated otherwise). EDTA solution (0.5 M, pH 8.0): add 95 mL ultra-pure water to 14.612 g EDTA, adjust the pH to 8.0 using NaOH solution, add 5 mL ultra-pure water; 1×Kinase Assay Buffer: add 25 mL HEPES solution (1 M), 190.175 mg EGTA, 5 mL MgCl2 solution (1 M), 1 mL DTT, 50 μL tween-20, 450 mL ultrapure water, adjust pH to 7.5 and constant volume to 500 mL using ultrapure water; 4×Stop solution (40 mM): Mix 0.8 mL of the foregoing EDTA solution, 1 mL 10×Detection Buffer and 8.2 mL ultra-pure water; 1×Detection Buffer: Mix 1 mL 10×Detection Buffer with 9 mL water; 4×VEGFR Kinase solution (1.74 nM, 521 times diluted, stored on ice): 1.2 μL VEGFR mother liquor (0.909 μM) was added to 624 μL 1×Kinase Assay Buffer and mixed; 4×ULight™-labeled JAK1 (substrate) (200 nM, 25 times diluted): 24 μL ULight™-labeled JAK1 (mother liquor concentration 5 μM) was added to 576 μL 1×Kinase Assay Buffer and mixed; 4×ATP Solution (40 μM, 250 times diluted): add 3 μL ATP solution (10 mM) to 747 μL 1×Kinase Assay Buffer and mixed; 4×Detection Mix (8 nM, 390.6 times diluted): 3 μL Europium- antiphospho-tyrosine antibody (PT66) (3.125 μM) was added to 1169 μL1×Detection Buffer and mixed; 2×substrate/ATP Mix: 560 μL foregoing 4×ULight™-labeled JAK1 and 560 μL 4×ATP solution and mixed (prepared before use). The assays used an ULight-labeled peptide substrate and a Europium-W1024-labeled antiphosphotyrosine antibody. The VEGFR-2 was purchased from Carna Biosciences, Inc. (New York, USA). The 384-well plates were obtained from PerkinElmer. Compounds were dissolved in DMSO and diluted to 11 concentrations at a tripling rate from 2.500 μM to 0.042 nM and added 2.5 μL to 384-well plates. 5 μL 2×VEGFR-2 kinase solution (0.5 nM) was added to 384-well plates homogeneous mixing and pre-reaction at room temperature for 30 min. Next, 2.5 μL 4×Ultra ULightTM-JAK-1(Tyr1023) Peptide (200 nM)/ATP (40 μM) was added to the corresponding wells of a 384-well plate. Negative control: 2.5 μL/well 4×substrate/ATP mixture and 7.5 μL 1×kinase assay buffer in 384-well plate well. Positive control: 2.5 μL/well 4×substrate/ATP mixture, 2.5 μL/well 1×kinase assay buffer with 16% DMSO, 5 μL/well 2×VEGFR-2 kinase solution was added to the 384-well plate, The final concentration of DMSO in the mixing system was 4%. After incubation at room temperature and dark for 60 min, 5 μL 4×stop solution was added to corresponding wells to react for 5 min and then 5 μL 4×detection mix was added to the corresponding wells of a 384-well plate. The mixture was centrifugally mixed and stayed for 60 min at room temperature for color development. The plate was read using an Envision plate reader. The inhibition rate (%) = (positive well reading-compound well reading)/(positive well reading-negative well reading)×100. The corresponding IC_50_ values were calculated using GraphPad Prism 5.0.

**4.2.5. Bax activation assay**

Bring all reagents, except the human Bax-α Standard, to room temperature for at least 30 minutes prior to opening. The human Bax-α Standard solution should not be left at room temperature for more than 10 minutes. All standards, controls and samples should be run in duplicate. Refer to the Assay Layout Sheet to determine the number of wells to be used and put any remaining wells with the desiccant back into the pouch and seal the ziploc. Store unused wells at 4 °C. Pipet 100 μL of Assay Buffer into the S0 (0 pg/mL standard) wells. Pipet 100 μL of Standards #1 through #6 into the appropriate wells. Pipet 100 μL of the Samples into the appropriate wells. Tap the plate gently to mix the contents. Seal the plate and incubate at room temperature on a plate shaker for 1 hour at ~500 rpm. Empty the contents of the wells and wash by adding 400 μL of wash solution to every well. Repeat the wash 4 more times for a total of **5 washes**. After the final wash, empty or aspirate the wells and firmly tap the plate on a lint free paper towel to remove any remaining wash buffer. Pipet 100 μL of yellow Antibody into each well, except the Blank. Seal the plate and incubate at room temperature on a plate shaker for 1 hour at ~500 rpm. Empty the contents of the wells and wash by adding 400 μL of wash solution to every well. Repeat the wash 4 more times for a total of **5** washes. After the final wash, empty or aspirate the wells and firmly tap the plate on a lint free paper towel to remove any remaining wash buffer. Add 100 μL of blue Conjugate to each well, except the Blank. Seal the plate and incubate at room temperature on a plate shaker for 30 minutes at ~500 rpm. Empty the contents of the wells and wash by adding 400 μL of wash solution to every well. Repeat the wash 4 more times for a total of **5 washes**. After the final wash, empty or aspirate the wells and firmly tap the plate on a lint free paper towel to remove any remaining wash buffer. Pipet 100 μL of Substrate Solution into each well. Incubate for 30 minutes at room temperature on a plate shaker at ~500 rpm. Pipet 100 μL Stop Solution to each well. Blank the plate reader against the Blank wells, read the optical density at 450 nm. Calculate the average net Optical Density (OD) bound for each standard and sample by subtracting the average Blank OD from the average OD for each standard and sample. Using linear graph paper, plot the Average Net OD for each standard versus Bax concentration in each standard. Approximate a straight line through the points. The concentration of Bax in the unknowns can be determined by interpolation.

**4.2.6. Bcl-2 inhibition assay**

Mix all the reagents thoroughly without foaming before use. Wash the microwells twice with approximately 300 μL Wash Buffer per well with thorough aspiration of microwell contents between washes. Take caution not to scratch the surface of the microwells. After the last wash, empty the wells and tap microwell strips on absorbent pad or paper towel to remove excess Wash Buffer. Use the microwell strips immediately after washing or place upside down on a wet absorbent paper for not longer than 15 minutes. Do not allow wells to dry. Add 100 μL of Sample Diluent in duplicate to all standard wells and to the blank wells. Prepare standard (1:2 dilution) in duplicate ranging from 32 ng/mL to 0.5 ng/mL. Add 100 μL of Sample Diluent, in duplicate, to the blank wells. Add 80 μL of Sample Diluent, in duplicate, to the sample wells. Add 20 μL of each Sample, in duplicate, to the designated wells. Add 50 μL of diluted biotin-conjugate to all wells, including the blank wells. Cover with a plate cover and incubate at room temperature, on a microplate shaker at 100 rpm if available, for 2 hours. Remove plate cover and empty the wells. Wash microwell strips 3 times as described in step 2. Add 100 μL of diluted Streptavidin-HRP to all wells, including the blank wells. Cover with a plate cover and incubate at room temperature, on a microplate shaker at 100 rpm if available, for 1 hour. Remove the plate cover and empty the wells. Wash microwell strips 3 times as described in step 2. Proceed to the next step. Pipette 100 μl of mixed TMB Substrate Solution to all wells, including the blanks. Incubate the microwell strips at room temperature (18° to 25°C) for about 15 minutes, if available on a rotator set at 100 rpm. Avoid direct exposure to intense light. The point, at which the substrate reaction is stopped, is often determined by the ELISA reader. Many ELISA readers record absorbance only up to 2.0 O.D. Therefore, the color development within individual microwells must be watched by the person running the assay and the substrate reaction stopped before positive wells are no longer properly detectable. Stop the enzyme reaction by quickly pipetting 100 μL of Stop Solution into each well, including the blank wells. It is important that the Stop Solution is spread quickly and uniformly throughout the microwells to completely inactivate the enzyme. Results must be read immediately after the Stop Solution is added or within one hour if the microwell strips are stored at 2 - 8°C in the dark. Read the absorbance of each microwell on a spectrophotometer using 450 nm as the primary wavelength.

**4.3. Docking Studies** Molecular docking simulations were performed to investigate the interactions between the synthesized quinazoline/1,3,4-oxadiazole derivatives and key cancer-related proteins, including EGFR and HER2, using Discovery Studio software. The protein structures were prepared and optimized for docking analysis, retaining only the essential chains for accurate binding studies. **4.4. Molecular dynamic simulations** A molecular dynamics (MD) simulation was conducted using GROMACS 2023 to validate the docking results and assess the stability of the interactions between the synthesized quinazoline/1,3,4-oxadiazole derivatives and the target HER2. The protein structures were prepared using UCSF Chimera, which included the addition of hydrogen atoms to ensure accurate geometry. The CHARMM36 force field was applied to proteins, while the CGenFF force field was used for the ligands. The protein-ligand complexes were immersed in a TIP3P water box with a 1 nm buffer zone to ensure proper hydration. Sodium chloride ions were added to neutralize the system, adjusting the concentration to 150 mM. Energy minimization was performed using the steepest descent method, followed by a two-phase equilibration process: a 100 ps NVT (constant number of particles, volume, and temperature) phase and a 100 ps NPT (constant number of particles, pressure, and temperature) phase at 300 K and 1.0 bar, with position restraints applied to the protein-ligand complex. A 100-ns production run was then conducted without restraints, during which trajectories were recorded every 10 ps for subsequent analysis. Key parameters, including root mean square deviation (RMSD) and binding energy, were calculated to evaluate the stability and dynamics of the protein-ligand interactions.

**4.5. Density functional theory (DFT) calculations** Density Functional Theory (DFT) calculations were conducted using the Becke three-parameter hybrid functional combined with the Lee-Yang-Parr correlation functional (B3LYP) and the 6-311+G(2d,p) basis set. In this phase of the study, geometry optimizations, frequency analyses, and molecular electrostatic potential (MEP) map calculations were performed. A frequency analysis was also conducted for the optimized structure to ensure that the geometry corresponded to true energy minima. All computational work in this section was performed using Gaussian 09 Rev.D01, and the results were visualized using GaussView 5.

**4.6. Statistical analysis**

Computerized Prism 5 program was used to statistically analyzed data using one-way ANOVA test followed by Tukey’s as post ANOVA for multiple comparison at P ≤.05. Data were presented as mean ± SEM.
